# Dynamical analysis of a general delayed HBV infection model with capsids and adaptive immune response in presence of exposed infected hepatocytes

**DOI:** 10.1007/s00285-024-02096-7

**Published:** 2024-04-30

**Authors:** Severin Foko

**Affiliations:** 1 Committed Mathematics Team, Research Unit in Mathematics and Applications, Department of Mathematics and Computer Science, Faculty of Science, University of Dschang, P.O. Box: 67, Dschang, Cameroon; 2 School of Computer Science and Applied Mathematics, University of the Witwatersrand, 1 Jan Smuts Avenue, Braamfontein, Johannesburg, Gauteng 2000 South Africa

**Keywords:** Hepatitis B virus infection, Adaptive immune response, General incidence function, Delays, Global stability, Sensitivity analysis, 92D25, 34D23

## Abstract

The aim of this paper is to develop and investigate a novel mathematical model of the dynamical behaviors of chronic hepatitis B virus infection. The model includes exposed infected hepatocytes, intracellular HBV DNA-containing capsids, uses a general incidence function for viral infection covering a variety of special cases available in the literature, and describes the interaction of cytotoxic T lymphocytes that kill the infected hepatocytes and the magnitude of B-cells that send antibody immune defense to neutralize free virions. Further, one time delay is incorporated to account for actual capsids production. The other time delays are used to account for maturation of capsids and free viruses. We start with the analysis of the proposed model by establishing the local and global existence, uniqueness, non-negativity and boundedness of solutions. After defined the threshold parameters, we discuss the stability properties of all possible steady state constants by using the crafty Lyapunov functionals, the LaSalle’s invariance principle and linearization methods. The impacts of the three time delays on the HBV infection transmission are discussed through local and global sensitivity analysis of the basic reproduction number and of the classes of infected states. Finally, an application is provided and numerical simulations are performed to illustrate and interpret the theoretical results obtained. It is suggested that, a good strategy to eradicate or to control HBV infection within a host should concentrate on any drugs that may prolong the values of the three delays.

## Introduction

As one of the most deadly and devastating recognized diseases worldwide, hepatitis B is a potentially life-threatening liver infection (Lok and McMahon 2007). It is an acute and chronic infection caused by the hepatitis B virus (HBV) a member of the DNA viruses in the family Hepadnaviridae (Chen et al. 2015), which is contracted through mainly contact with blood or other bodily fluids. HBV infection is related to the major global health problems as it can cause chronic infection and lead to high risk of death from primary hepatocellular carcinoma, liver failure, liver cancers or cirrhosis and acute renal failure, which is an additional new complication attributable to HBV (Chen et al. 2015; Kishi 2013). In 2010, chronic HBV was ranked as the fifteenth cause of mortality through the world, with about 800 000 deaths of the total (Lavanchy and Kane 2016).

It is widely admitted that the parenchymal cell of the liver, called hepatocyte, is the primary site of HBV infection (Guidotti and Chisari 2006). HBV is an enveloped hepatotropic virus containing a relaxed circular partially double-stranded DNA genome with is 3.2 kb in length (Guo et al. 2018; Guo 2007; Ribeirom et al. 2002; Tu 2021). The life cycle of HBV can be detailed as follows. First, during hepatocytes infection, viral genomic DNA is converted into an episomal covalently closed circular DNA (cccDNA) inside the nucleus of the infected hepatocyte and serve as the template for mRNA production (Guo 2007; Lewin et al. 2002; Ribeirom et al. 2002; Tu 2021). Thenceforth, several copies of cccDNA create the pregenomic and subgenomic mRNA and can be transcribed into at least four major viral RNAs (Guo et al. 2018; Guo 2007; Manna 2017; Ribeirom et al. 2002; Tu 2021). Afterwards, the polymerase and pgRNA are encapsidated into the viral nucleocapsid, indicating the genome replication initiation (Lentz and Loeb 2010). Next, pgRNA follows reverse transcription procedure to transform into a double-stranded HBV DNA (Guo et al. 2018; Lewin et al. 2002; Manna 2017; Murray et al. 2006). Finally, this leads to the production of HBV DNA-containing capsid and then a part of freshly produced HBV DNA-containing capsid is transmitted to plasma under HBV core particle form and packed by HBsAg to create the complete free virions (Guo et al. 2018; Manna 2017), another part of HBV core particle is reemployed for the next replication cycle (Guo et al. 2018). Therefore, capsid represents the protein coat surrounding the nucleic acid of a virus. It plays an important role in virus formation and replication during the maturation phase of the free virions (Bruss 2004; Grimm et al. 2011; Pairan and Bruss 2009). Free HBV particles can lead to develop viral persistence in the patients when strong cytotoxic T lymphocytes (CTL) and antibody immune defense are absents. But, it is worth mentioning that HBV can replicate and duplicate within target cells without causing direct cell damage (Tan et al. 2015). The defense against HBV infection pathogen is a major function of the adaptive immunity (Bertoletti and Ferrari 2016). The adaptive immunity, which is constituted of CTLs and antibody B cells, has been recognized as a main crucial player in the clearance of HBV infection (Tan et al. 2015). When CTLs attack and kill infected hepatocytes to reduce HBV load, B cells attack and neutralize free virions to prevent reinfection process. Hence, focusing on the adaptive immunity activation states, may provide new strategies for evaluating immune status of HBV infection, policing progression of hepatitis B and predicting efficacy of antiviral treatment (Li et al. 2014).

Over the past few decades, several mathematical models have been developed and studied to explore mechanisms and within-host viral infection dynamics process by employing ordinary differential equations (ODEs), delay differential equations (DDEs) and partial differential equations (PDEs). These models provide insights into in vivo viral load dynamics and play a significant role in the development of a better understanding of HBV infection. Furthermore, as samples cannot always be taken frequently from patients, or detection techniques of the free virion may not be faithful to the truth, testing specific hypotheses based on clinical experimental data remains a worthwhile challenge, justifying the role played by mathematical models in this area. The history of mathematical modeling of the dynamics of HBV infection transmission begins with the pioneering work of Nowak and his co-workers (Nowak et al. 1996). Their model consists of ODE and investigates the relation between uninfected hepatocytes, infected hepatocytes and free viruses. They gave a quantitative understanding of HBV replication dynamics in vivo. After that, many other models have been designed to improve the shortcomings observed and extend this basic model by including other forms of infection rates or additional components. We can cite the work of Nowak and Bangham, which extended this baseline model by incorporating cytotoxic T lymphocyte immune responses in Nowak and Bangham (1996). By building Lyapunov functions, Korobeinikov (2004) established the global stability of system proposed in Nowak et al. (1996). Min and co-workers (Min et al. (2008)) amended this basic viral infection model by replacing the mass action term by a standard incidence function for the infection process. Wang and his collaborators (Wang et al. 2010) extended the model in Nowak et al. (1996) by taking into account the cytokine-mediated cure of infected liver cells and investigated a global stability analysis. Hews et al. (2010) extended the basic model in Nowak et al. (1996) by replacing the constant infusion of healthy hepatocytes with a logistic growth term and the mass action term by a standard incidence function. A similar type of model for HBV infection with logistic hepatocyte growth and mass action term was formulated and analyzed in Li et al. (2011). Manna and Chakrabarty (2015a) were the first to model HBV infection by incorporating both uninfected hepatocytes and HBV DNA-containing capsids. Meskaf et al. (2023) investigated an ODE model of hepatitis B with capsids by considering the proliferating of its dynamics following logistic growth function and saturated incidence rate. The dynamics of a viral infection model with Crowley-Martin type functional response was studied in Xu (2012). The models including the role of the adaptive immunity in fighting the free virions and reducing the infected hepatocytes were investigated in Harroudi et al. (2020), Jiang and Wang (2014), Yousf et al. (2011). It is worthy noting that the above models do not take into account the time delay. As a matter of fact, for HBV infection and many other infectious diseases, it is important to consider the influences of delays on the dynamics transmission of the disease. This is justified by the fact that in epidemiological models, delay can be caused by a variety of factors (Geng et al. 2018). Also, from the life cycle of HBV, it can be seen that the different stages of the evolution of HBV in the hepatocytes do not take place at the same time. In order to take into account the effect of time delay, in the literature (Eikenberry et al. 2009), the authors proposed a delayed HBV infection model with logistic hepatocyte growth. From the obtained model, they demonstrated the existence of sustained oscillations aside from the stability of the biological relevant equilibria and their bifurcation behavior. Wang and Tian (2013) discussed the global stability properties of a delayed HBV infection model with CTL immune response. By using a simple ODE version of the HBV infection model, Murray and co-workers (Murray et al. 2006) found that the half-life of HBV virions is approximately 4 h. Manna and Chakrabarty (2017) presented and analyzed the dynamical behaviours of an HBV infection model with capsids and two discrete delays. Dixit and Perelson (2004) estimated that the time delay for virus production is approximately one day.

By using the assumption, made in Wang and Wang (2007), that the motion of virus follows the Fickian diffusion, many authors incorporated the spatial dependence in the modeling of HBV infection process in vivo. Tadmon and Foko (2019) extended the work in Wang and Wang (2007) by incorporating logistic growth term and by replacing the mass action term by a standard incidence function. Afterward, in Tadmon and Foko (2020), they considered the spatiotemporal model in Tadmon and Foko (2019) and constructed two different discrete models by using the nonstandard finite difference method. Manna (2018) made an extension of the reaction-diffusion HBV infection model developed in Manna (2017) which studied the role of CTL immune response. Geng et al. (2018) extended the model presented in Manna and Chakrabarty (2015b) by considering the mobility of capsids and free viruses. They used the nonstandard finite difference scheme to obtained a discrete model of the corresponding continuous HBV infection model with capsids. In Guo et al. (2018), the authors formulated a three delays spatiotemporal HBV infection model with general incidence functional and capsids, where the third delay is taken in to account in the production of matured free viruses. In Miao et al. (2018), the global stability of a two-time-delayed reaction-diffusion model with general incidence rate and adaptive immunity was investigated by employing appropriate Lyapunov functionals and LaSalle’s invariance principle. Recently, Manna and Hattaf (2019), Miao et al. (2018), Danane and Allali (2018) and Elaiw and Agha (2019), in this order, were the first to contain both capsids and adaptive immunity aside from uninfected cells, infected cells and free viruses. However, In Manna and Hattaf (2019), Miao et al. (2018) and Elaiw and Agha (2019), the authors considered the spatial mobility of capsids and free viruses and investigated the global stability of the homogeneous equilibria by using suitable Lyapunov functionals. But, in Danane and Allali (2018), the authors ignored the random mobility of capsids and free virus particles and did not also discussed the global stability of the equilibrium points. We first note that the threshold parameters of these models are independent to the diffusion coefficients of capsids and free viruses. This indicates that diffusion of capsids and free viruses have no effect on the global dynamical behavior of their models. Next, we note that all the models developed in the above aforementioned works have ignored an explicit equation for exposed infected hepatocytes and then assumed that the uninfected hepatocytes which are exposed to free virions immediately become infected. Gourley et al. (2008) proposed a global dynamics of a simple ODE model of HBV with time delay and exposed infected cells, and used the method of step to investigate the positivity of solutions. In Elaiw (2015), the author developed a mathematical virus dynamics model with Beddington-DeAngelis functional response and humoral immunity including latently infected cells. He ignored the spatial diffusion of free viruses and said that the proposed model may describe the dynamics of HBV infection. In this case, the latently infected cells become the exposed infected hepatocytes. Very recently, Tadmon et al. (2021) investigated a delayed spatiotemporal HBV infection model in presence of humoral immune response and exposed infected hepatocytes. Foko and Tadmon (2022) proposed and analyzed a general diffusive within-host HBV dynamics model with capsids, adaptive immunity and two categories of infected hepatocytes: exposed infected hepatocytes and productively infected hepatocytes. The model assumed that the different stages of the evolution of intracellular HBV replication in the hepatocytes and the maturation process of the capsids as well as free virus particles are instantaneous despite the fact that time delay actually exists at each stage (Guo et al. 2018; Manna and Hattaf 2019). By employing the nonstandard finite difference method, they studied the dynamics of fundamental properties of both discrete and continuous models and shown that the discrete system is dynamically consistent with the continuous model. To the best knowledge of ours, there does not exit any work in the literature which incorporates at once capsids, exposed infected hepatocytes, adaptive immunity and delay for the modeling of HBV infection process in vivo.

Therefore, the objective of this work is threefold. Firstly, to propose a general mathematical model extending the work in Elaiw (2015), Elaiw and Agha (2019), Foko and Tadmon (2022), Gourley et al. (2008), Manna and Hattaf (2019) by the incorporation of two categories of infected hepatocytes: exposed infected hepatocytes and productively infected hepatocytes; secondly, to rigorously study the coming model by addressing the global stability properties of the model; finally, to provide an application of the model generalized and through this, carry out local and global sensitivity analysis of the basic reproduction number and of the classes of infected states, and perform numerical simulations through which, we present the impacts of the time delays and mortalities during these time delays.

For the reason stated above, we will neglect the spatial mobility of capsids and free viruses.

The model is built base on the model developed in Foko and Tadmon (2022). Assuming that the different stages of the evolution of HBV in the hepatocytes do not take place at the same time, we extend this model by incorporating three time delays and mortalities during the three time delays. We derive five threshold parameters, and show, employing the method of global Lyapunov function, that the global dynamics of the model is completely determined by the range of the five threshold parameters. From a sensitivity analysis of the basic reproduction number and of the classes of infected states, we are able to discuss the impact of parameters that significantly affect the basic reproduction number and the classes of infected states. The Lyapunov functionals employed in this paper to prove the global stability of all possible equilibria have the same form as those used in Elaiw and Agha (2019), Manna and Hattaf (2019) when the diffusion of capsids and free viruses is neglected.

The work is organized as follows. In Sect. 2, we present the relevant biological assumptions for the construction of the model of HBV infection in vivo. The mathematical model for the within-host dynamics of HBV infection presenting the interactions between intracellular HBV DNA-containing capsids, free viruses, adaptive immunity and hepatocytes is also proposed. In Sect. 3, we investigate the mathematical analysis of the established model. We prove global existence, uniqueness, non-negativity and boundedness of the solution to the obtained model. In Sect. 4, we define the threshold parameters and discuss the existence of all possible homogeneous equilibria. Section 5 is devoted to the stability properties of all possible equilibrium points by using the crafty Lyapunov functionals, the LaSalle’s invariance principle and linearization methods. In Sect. 6, an application is given to confirm the theoretical results obtained. Finally, a conclusion and discussion are drawn in Sect. 7.

## Model construction

We begin this section by describing the process of HBV infection. We note that after entering the body, the HBV is driven into the liver through the bloodstream. Then it binds to receptors situated at the surface of a susceptible hepatocyte. Thereafter, these hepatocytes go through an exposed stage, during which they change to produce immature viral capsids after $\tau _1$ units of time, where $\tau _1$ denotes the time necessary to construct, transcribe and translate the episomal viral DNA genome, fabricate and then release the first new immature capsids. In addition, at the same $\tau _1$ units of time, exposed infected hepatocytes convert to productively infected hepatocytes, which in turn contributes to the production of matured intracellular HBV DNA-containing capsids after $\tau _2$ units of time, where $\tau _2$ means the time spend needed for that production. More specifically, the intracellular delay $\tau _1$ describes the exposure period between the time when target cells are exposed and the time when exposed infected hepatocytes become actively infected and the immature viral capsids are fabricated. The newly activated infected target cells at time *t* are such that a quantity is the survival rate of virion-infected hepatocytes at time *t* and become activated at $\tau _1$ time later. The intracellular delay $\tau _2$ describes the time between viral capsids release and maturation. Then, the number of mature capsids produced at time *t* is such that a fraction is the survival rate of hepatocytes that start budding from activated infected hepatocytes at time *t* and become mature capsids at $\tau _2$ time later. Now, after maturation, capsids are released and become new virions after $\tau _3$ units of time, where $\tau _3$ denotes the time needed for the newly produced virions to become mature. On the other words, the virus replication delay $\tau _3$ describes the time between viral release and maturation. Then, the number of mature viral particles generated at time *t* is such that a quantity is the survival rate of capsids that will be, in life at time *t* and become free mature viruses at $\tau _3$ time later. Finally, a general incidence rate may help us to obtain the unification theory by omitting unessential details. Inspired by the aforementioned process, we formulate the following HBV infection model with capsids, adaptive immunity, three time delays and a general incidence rate, and including both the number of exposed infected hepatocytes and productively infected hepatocytes:

**Fig. 1:**
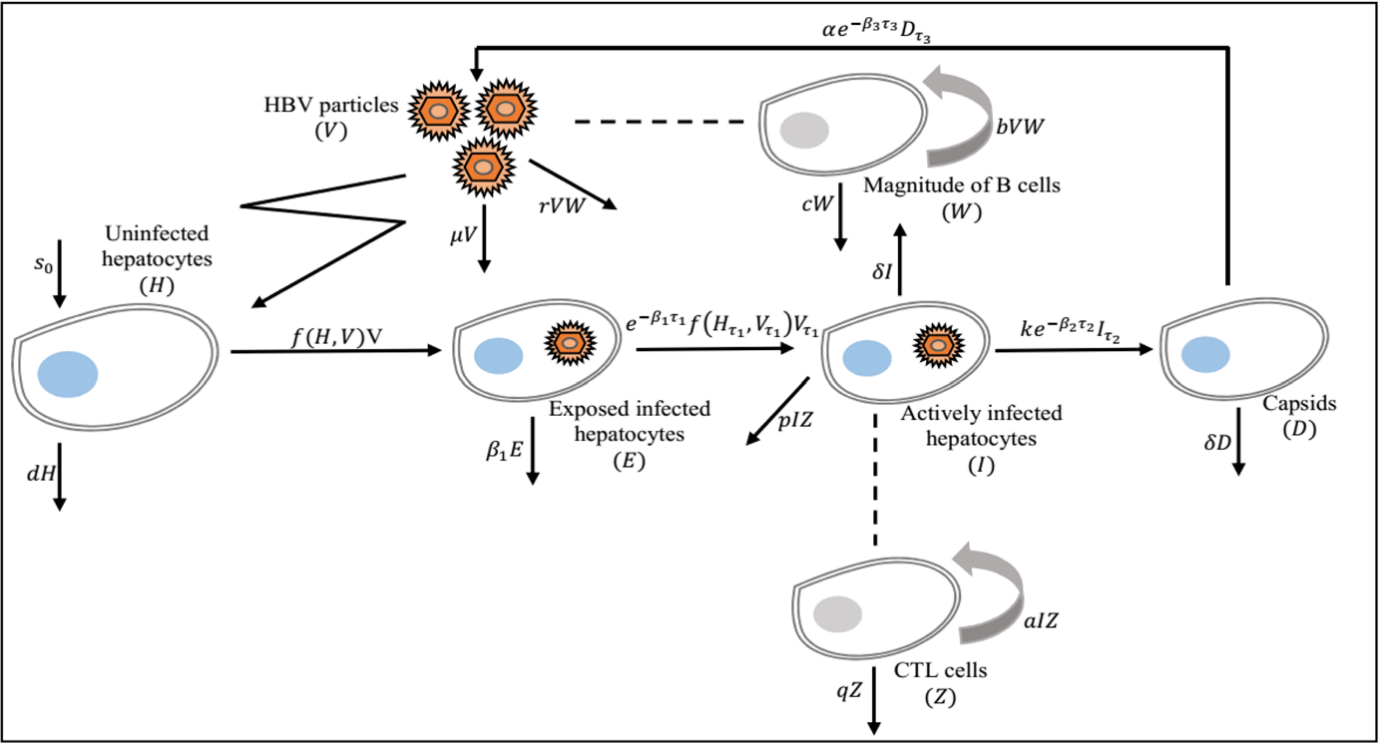
A schematic diagram of the model (2.1)

2.1 $$\begin{aligned} {\left\{ \begin{array}{ll} \frac{d H}{d t}=s_0-dH(t)-f(H(t),V(t))V(t),\\ \frac{d E}{d t}=f(H(t),V(t))V(t)- e^{-\beta _1\tau _1}f(H(t-\tau _1),V(t-\tau _1))V(t-\tau _1)-\beta _1E(t),\\ \frac{d I}{d t}= e^{-\beta _1\tau _1}f(H(t-\tau _1),V(t-\tau _1))V(t-\tau _1)-\delta I(t)-pI(t)Z(t),\\ \frac{d D}{d t}=k e^{-\beta _2\tau _2}I(t-\tau _2)-(\alpha +\delta )D(t),\\ \frac{d V}{d t}=\alpha e^{-\beta _3\tau _3}D(t-\tau _3)-\mu V(t)-rV(t)W(t),\\ \frac{d W}{d t}=bV(t)W(t)-cW(t),\\ \frac{d Z}{d t}=aI(t)Z(t)-qZ(t). \end{array}\right. } \end{aligned}$$

For biological reasons, the initial conditions for model (2.1) take the form

2.2 $$\begin{aligned} \left. \begin{array}{ll} H(\theta )=\phi _1(\theta ), \qquad E(\theta )=\phi _2(\theta ), \qquad I(\theta )=\phi _3(\theta ),\\ D(\theta )=\phi _4(\theta ),\qquad V(\theta )=\phi _5(\theta ),\qquad W(\theta )=\phi _6(\theta ),\qquad \\ Z(\theta )=\phi _7(\theta ),\qquad \theta \in [-\tau , 0],\; \tau =\max \{\tau _1,\tau _2,\tau _3\}, \; \phi _i(0)>0,\; i=1,\ldots ,7. \end{array}\right. \end{aligned}$$

where $\phi _1(\theta ),$ $\phi _2(\theta ),$ $\phi _3(\theta ),$ $\phi _4(\theta ),$ $\phi _5(\theta ),$ $\phi _6(\theta )$ and $\phi _7(\theta )\in {\mathcal {C}}_+$ and $\phi =(\phi _1,\phi _2,\phi _3,\phi _4,\phi _5,\phi _6,\phi _7)\in {\mathcal {C}}_+\times {\mathcal {C}}_+\times {\mathcal {C}}_+\times {\mathcal {C}}_+\times {\mathcal {C}}_+\times {\mathcal {C}}_+\times {\mathcal {C}}_+$

The biological terms *H*(*t*),  *E*(*t*),  *I*(*t*),  *D*(*t*),  *V*(*t*),  *W*(*t*) and *Z*(*t*) represent the densities of uninfected hepatocytes, exposed infected hepatocytes, productively infected hepatocytes, HBV DNA-containing capsids, free viruses, the magnitude of B cells and CTL cells at time *t*. The uninfected hepatocytes are created at a constant rate $s_0,$ either from differentiation of progenitor cells or by direct proliferation of mature hepatocytes (Ribeirom et al. 2002). Moreover, uninfected hepatocytes die at rate *dH* and become exposed infected through free virus at rate *f*(*H*, *V*)*V*. The second equation of model (2.1) describes the behavior of exposed infected hepatocytes, that is, hepatocytes that have been infected but are not yet producing new capsids, and shows that they die at rate $\beta _1E.$ We assume that all infected cells initially enter a period of exposure to infection that last exactly $\tau _1$ days. That is, the delay parameter $\tau _1$ represents the time necessary for exposed cells to convert to productively infected and then produce immature viral capsids. This implicitly assumes that exposed infected hepatocytes are not targeted by the CTL immune defense. Recall that after $\tau _1$ day, exposed infected hepatocytes become actively infected. Therefore, all hepatocytes infected $t-\tau _1$ days ago, where *t* stands for the current time, will either transition to the active class at the proportion $1-e^{-\beta _1\tau _1}$ or die in the meantime at the proportion $e^{-\beta _1\tau _1}.$ Thus, the quantity $e^{-\beta _1\tau _1}$ denotes the probability of surviving of hepatocytes infected from $t-\tau _1$ to *t*,  where $\beta _1$ denotes the death rate for exposed infected hepatocytes that are not yet actively infected. The third equation of system (2.1) describes the behavior of hepatocytes that are actively producing capsids. The transition from the exposure to active infection has already been described. The parameter *k* denotes the production rate of HBV DNA-containing capsids from actively infected hepatocytes, while $\alpha$ indicates the rate at which these capsids are transmitted to blood with a view to convert into free virions. In Murray et al. (2005), the authors by considering that clearance of infection proceeds through death of infected cells, they assumed that the amount of death per day of total infected hepatocytes and HBV DNA-containing capsids is proportional to some maximum related to alanine transaminase levels. But in Murray et al. (2006), the authors, based on this assumption, developed a simplified version of the HBV infection model described in Murray et al. (2005), in which they take the same death rate for both infected hepatocytes and HBV DNA-containing capsids. In the same vein, in this formulation, $\delta$ denotes the per capita death rate of productively infected hepatocytes as well as HBV DNA-containing capsids. The biological parameter $\mu$ denotes the clearance rate of virions in plasma. Productively infected hepatocytes are removed by CTL cells at rate *pIZ* whereas free virions are neutralized by antibodies at a rate *rVW*. Besides, B cells expand in response to free virions at rate *bVW* and decreased at rate *cW*. CTL cells are activated by infected hepatocytes at rate *aIZ* and decreased in the absence of antigenic stimulation at rate *qZ*. Delay parameter $\tau _2$ is the time for capsids to become mature before produce free virions. From the literatures (Bruss 2004; Ganem and Prince 2004), we note that the productively infected hepatocytes release the HBV DNA-containing capsids under the form of mature virions after being enveloped by cellular membrane lipids and viral envelope proteins. The term $e^{-\beta _2\tau _2}$ denotes the survival probability of immature capsids and ${1}/{\beta _2}$ represents the average lifetime of an immature capsids, where $\beta _2$ is the decay rate of immature capsids newly produced during the time period $[t-\tau _2,t]$. Delay parameter $\tau _3$ represents the time needed for newly produced HBV DNA-containing capsids to become free virions. The quotient $e^{-\beta _3\tau _3}$ is the probability of survival of immature virions over the interval time period $[t-\tau _3,t]$ and ${1}/{\beta _3}$ denotes the average lifetime of immature virions, where $\beta _3$ is the decreasing rate of new immature virions produced during the delay period. Now, taking in to account the CTL and antibodies immune response time delays, system (2.1) become

2.3 $$\begin{aligned} {\left\{ \begin{array}{ll} \frac{d H}{d t}=s_0-dH(t)-f(H(t),V(t))V(t),\\ \frac{d E}{d t}=f(H(t),V(t))V(t)- e^{-\beta _1\tau _1}f(H(t-\tau _1),V(t-\tau _1)) V(t-\tau _1)-\beta _1E(t),\\ \frac{d I}{d t}= e^{-\beta _1\tau _1}f(H(t-\tau _1),V(t-\tau _1))V(t-\tau _1) -\delta I(t)-pI(t)Z(t),\\ \frac{d D}{d t}=k e^{-\beta _2\tau _2}I(t-\tau _2)-(\alpha +\delta )D(t),\\ \frac{d V}{d t}=\alpha e^{-\beta _3\tau _3}D(t-\tau _3)-\mu V(t)-rV(t)W(t),\\ \frac{d W}{d t}=be^{-\beta _4\tau _4}V(t-\tau _4)W(t-\tau _4)-cW(t),\\ \frac{d Z}{d t}=ae^{-\beta _5\tau _5}I(t-\tau _5)Z(t-\tau _5)-qZ(t). \end{array}\right. }\end{aligned}$$

Here, since the antigenic activation generating CTL cells may require a period of time lag, it can be assume that CTL produced at time *t* depends on the number of CTL and productively infected hepatocytes at time $t-\tau _5,$ for a time lag $\tau _5>0.$ Thus, the newly proliferated CTL cells at time *t* are given by a delayed term $ae^{-\beta _5\tau _5}I(t-\tau _5) Z(t-\tau _5),$ where the quantity $e^{-\beta _5\tau _5}$ stands for the proportion of CTL cells that can survive from time $t-\tau _5$ to time *t*. Similarly, antigenic stimulation generating B cells may also need a period of time $\tau _4$ i.e., the *B* cells response at time *t* may depend on the population of antigen at a previous time $t-\tau _4.$ Then, we propose the form $be^{-\beta _4\tau _4}V(t-\tau _4)W(t-\tau _4)$ to model the antibodies immune response in system (2.3), where the fraction $e^{-\beta _4\tau _4}$ denotes the proportion of B cells that can survive from time $t-\tau _4$ to time *t*. Now, in Pang and Cui (2017) by analyzing a HBV infection model with immune response delay, the authors concluded that majority of hepatitis B infection would eventually become a chronic infection due to the immune response time delay which is fairly long. In Yosyingyong and Viriyapong (2023), the authors arrived to the same conclusion for a six compartmental HBV infection model with capsids and adaptive immune response, and only the delay in the productively infected hepatocytes and in an antigenic stimulation generating CTL. Therefore, we first neglect the CTL and antibodies immune response time delays and explore the dynamical behavior of the model (2.1) with only the above three time delays. Investigation of the model (2.3) taking into account the CTL and antibodies immune response time delays will be the concern of a forthcoming work via an in-depth analysis of the system obtained. Figure 1 exhibits the connection between seven compartments and model parameters. The general incidence function *f*(*H*, *V*) is assumed to be continuously differentiable in the interior of ${\mathbb {R}}^4_+.$ Furthermore, we assume that *f* satisfies the following hypotheses.

### Hypothesis 2.1

We assume that:

$$(B_1)$$
$$f(0,V)=0,$$ for all $$V\ge 0,$$
$$(B_2)$$
*f*(*H*, *V*) is a strictly monotonically nondecreasing function with respect to *H*,  that is, $\frac{df(H,V)}{dH}>0$, for any fixed $H>0$ and $V\ge 0,$
$$(B_3)$$
*f*(*H*, *V*) is a monotonically nonincreasing function with respect to *V*,  that is, $\frac{df(H,V)}{dV}\le 0,$ for $H\ge 0,$ and $V\ge 0,$

Biologically, the three hypotheses above are reasonable and accordant with the reality. More precisely, assumption $(B_1)$ indicates that HBV infection cannot spread if there are no new exposed infected hepatocytes and actively infected hepatocytes (that is $f(H,V)=0$) without healthy hepatocytes ($H=0$) or virus particles ($V=0$). Requirement $(B_2)$ implies that the incidence function *f*(*H*, *V*)*V* becomes faster as the densities of free viruses are constant and the density of healthy hepatocytes increases. Thus, if the total number of free virion is constant, then the more the amount of healthy hepatocyte is, the more the average number of hepatocytes which are exposed through each virus and then converted to productively infected in the unit time will be. Assumption $(B_3)$ indicates that the per capita infection rate by free virions will slow down due to inhibition influence. Thus, if the total number of healthy hepatocytes is constant, then the more the amount of free virions is, the less the average number of hepatocytes which are infected by each free virion in the unit time will be. Furthermore, for $\beta _0, a_0, b_0, \kappa _0, c_1>0,$ we can check that class of general incidence function *f*(*H*, *V*) satisfying Hypothesis 2.1$(B_1)$–$(B_3)$ include several incidence function forms such as $f(H,V)=\beta _0H$ in Manna (2018), $f(H,V)=\frac{\beta _0H}{H+V}$ in Hattaf and Yousfi (2016), Zhuo (2012), $f(H,V)=\frac{\beta _0H}{1+b_0V}$ in Xu and Ma (2009), $f(H,V)=\frac{\beta _0H}{1+a_0H+b_0V}$ in Beddington (1975), DeAngelis et al. (1975), Zhang and Xu (2014), $f(H,V)=\frac{\beta _0H}{1+a_0H+b_0V+a_0b_0HV}$ in Kang et al. (2017) and $f(H,V)= \frac{\beta _0H}{\kappa _0+a_0H+b_0V+c_1HV}$ in Hattaf and Yousfi (2016).

We aim in this paper to study the dynamical properties of model (2.1). Specifically, the stability of all possible homogeneous equilibria, which induces the behaviors of the proposed model, will be investigated.

## Basic properties results

Consider the Banach space ${\mathcal {C}}=C([-\tau ,0],{\mathbb {R}}),$ of continuous functions from $[-\tau , 0]$ to ${\mathbb {R}}$ endowed with the usual supremum norm. The nonnegative cone of ${\mathcal {C}}$ is defined by ${\mathcal {C}}_+=C([-\tau ,0],{\mathbb {R}}_+).$ By the fundamental theory of functional differential equations (Hale and Verduyn Lunel (1993)), it is known that there exists a unique solution (*H*(*t*), *E*(*t*), *I*(*t*), *D*(*t*), *V*(*t*), *W*(*t*), *Z*(*t*)) of model (2.1) satisfying initial conditions (2.2).

From Theorem 2.1 in Cooke and van den Driessche (1996), we have the following result.

### Theorem 3.1

Let (*H*(*t*), *E*(*t*), *I*(*t*), *D*(*t*), *V*(*t*), *W*(*t*), *Z*(*t*)) be any arbitrary solution of model (2.1), with initial conditions given in (2.2). If in addition we have the following compatibility condition

3.1 $$\begin{aligned} \phi _2(0)=E(0)=\int _{-\tau _1}^{0}e^{\beta _1\theta }f(\phi _1(\theta ),\phi _4(\theta ))\phi _4(\theta )d\theta , \end{aligned}$$

then this solution satisfies the following integro-differential equation system

3.2 $$\begin{aligned} {\left\{ \begin{array}{ll} \frac{d H}{d t}=s_0-dH(t)-f(H(t),V(t))V(t),\\ E(t)=\displaystyle \int _{t-\tau _1}^{t}e^{-\beta _1(t-\theta )}f(H(\theta ),V(\theta ))V(\theta )d\theta ,\\ \frac{d I}{d t}= e^{-\beta _1\tau _1}f(H(t-\tau _1),V(t-\tau _1))V(t-\tau _1)-\delta I(t)-pI(t)Z(t),\\ \frac{d D}{d t}=k e^{-\beta _2\tau _2}I(t-\tau _2)-(\alpha +\delta )D(t),\\ \frac{d V}{d t}=\alpha e^{-\beta _3\tau _3}D(t-\tau _3)-\mu V(t)-rV(t)W(t),\\ \frac{d W}{d t}=bV(t)W(t)-cW(t),\\ \frac{d Z}{d t}=aI(t)Z(t)-qZ(t). \end{array}\right. } \end{aligned}$$

Conversely, any arbitrary solution of the integro-differential equation system (3.2) satisfies the second equation of system (2.1).

### Proof

The converse is obvious. Now, we prove the first assertion. From the second equation of system (2.1), we get

$$\begin{aligned} \dfrac{d}{dt} \left( E(t)e^{\beta _1t}\right) =e^{\beta _1t}(f(H(t),V(t))V(t)- e^{-\beta _1\tau _1}f(H(t-\tau _1),V(t-\tau _1))V(t-\tau _1)), \end{aligned}$$

which is equivalent to

$$\begin{aligned}{} & {} \dfrac{d}{dt} \left( E(t)e^{\beta _1t}\right) =\dfrac{d}{dt}\left\{ \displaystyle \int _{t-\tau _1}^{t}e^{\beta _1\theta }f(H(\theta ),V(\theta ))V(\theta )d\theta \right\} . \end{aligned}$$

Integrating this over [0, *t*],  gives

$$\begin{aligned} E(t)= & {} e^{-\beta _1t}\left( E(0)-\int _{-\tau _1}^{0}e^{\beta _1\theta }f(H(x,\theta ),V(\theta ))V(\theta )d\theta \right) \\{} & {} +\displaystyle \int _{t-\tau _1}^{t}e^{-\beta _1(t-\theta )}f(H(x,\theta ),V(\theta ))V(\theta )d\theta . \end{aligned}$$

Using (2.2) and (3.1), the second equation of system (3.2) is established. This achieves the proof. $\square$

The following result establishes the well-posedness of solutions of model (2.1) with initial conditions (2.2).

### Theorem 3.2

Let Hypotheses $(B_1)$–$(B_3)$ hold. Then, solutions of model (2.1) with initial conditions (2.2) are positive and ultimately uniformly bounded for $t>0.$

### Proof

From the last two equations in (2.1), we have

$$\begin{aligned} W(t)= & {} \phi _6(0)\exp \left\{ -ct+b\int _0^tV(s)ds\right\}>0, \quad \text {for}\quad t\ge 0 \quad \text {and}\\ Z(t)= & {} \phi _7(0)\exp \left\{ -qt+a\int _0^tI(s)ds\right\} >0,\quad \text {for}\quad t\ge 0. \end{aligned}$$

Now, we prove in this order that *I*,  *D*,  *V* *H* and *E* are positive.

By the third, fourth and fifth equations in (2.1), we get

3.3 $$\begin{aligned} I(t)= & {} \phi _3(0)\exp \left\{ -\delta t-p\int _0^tZ(s)ds\right\} \nonumber \\{} & {} +\int _0^t\Big \{e^{-\beta _1\tau _1}f(H(\phi -\tau _1),V(\phi -\tau _1))V(\phi -\tau _1)\Big \}\nonumber \\{} & {} \times e^{\delta (\phi -t)}\exp \left\{ -p\int _\phi ^{t}Z(s)ds\right\} d\phi , \end{aligned}$$

3.4 $$\begin{aligned} D(t)= & {} \left[ \phi _4(0)+k e^{-\beta _2\tau _2} \int _0^tI(\phi -\tau _2)e^{(\alpha +\delta )\phi }d\phi \right] e^{-(\alpha +\delta )t}, \end{aligned}$$

3.5 $$\begin{aligned} V(t)= & {} \phi _5(0)e^{-\int _0^tz(s)ds}+\alpha e^{-\beta _3\tau _3}\int _0^tD(\phi -\tau _3)e^{-\int _\phi ^tz(s)ds}d\phi , \end{aligned}$$

where $z(t)=\mu +rW(t).$ Let $t\in [0,\tau ],$ where $\tau =\max \{\tau _1,\tau _2,\tau _3\}.$ Then one has $\phi -\tau \in [-\tau ,0]$ for all $\phi \in [0,\tau ].$ $H(t)\ge 0,$ $I(t)\ge 0,$ $D(t)\ge 0,$ $V(t)\ge 0$ for $t\in [-\tau ,0]$ and $H(0)>0,$ $I(0)>0,$ $D(0)>0,$ $V(0)>0.$ If $t\in [0,\tau ],$ then the second term of (3.3) is non-negative, therefore $I(t)>0.$ Accordingly, the second terms of (3.4) is positive, implying $D(t)>0.$ This in turn implies that the second terms of (3.5) is positive, which means that $V(t)>0.$

Next, let $t_1$ be the first value of *t* such that $H(t_1)=0.$ If $t_1\le \tau ,$ then from the first equation of system (2.1) and hypothesis $(B_1),$ we obtain that

$$\begin{aligned} \frac{d H(t)}{d t}\bigg |_{t=t_1}=s_0>0, \end{aligned}$$

which give us a contradiction, because this implies that there exists an $\varepsilon >0$ such that $H(t)<0$ for $t\in (t_1-\varepsilon , t_1).$ Thus $H(t)>0$ for all $t\in [0,\tau ].$

Now, let $t_2$ be the first value of *t* such that $E(t_2)=0.$ If $t_2\le \tau ,$ then, since $E(t)=\int _{t-\tau _1}^{t}e^{-\beta _1(t-s)}f(H(s)$, *V*(*s*))*V*(*s*)*ds* by Theorem 3.1, we have

$$\begin{aligned} E(t_2)=\int _{t_2-\tau _1}^{t_2}e^{-\beta _1(t_2-s)}f(H(s),V(s))V(s)ds>0, \end{aligned}$$

a contradiction. Thus $E(t)>0$ for all $t\in [0,\tau ].$ Therefore, we have demonstrated that $H(t)>0,$ $E(t)>0,$ $I(t)>0,$ $D(t)>0,$ $V(t)>0$ for all $t\in [0,\tau ].$

By repeating the above arguments, it can be shown that the variables *H*,  *E* *I*,  *D* and *V* are positive on successive interval $[n\tau ,(n+1)\tau ], n=1,2,\ldots ,$ where all include times are positive.

Now, it remains to prove the boundedness of solutions. For this, consider the following functional

3.6 $$\begin{aligned} Y(t)= & {} H(t)+E(t)+I(t)+\dfrac{\delta }{kn}D(t)+\dfrac{\delta (\alpha +\delta )}{k\alpha m}V(t)+\dfrac{p}{a}Z(t)+\dfrac{r\delta (\alpha +\delta )}{k\alpha bm}W(t)\nonumber \\{} & {} +\dfrac{\delta }{n}\int _{t-\tau _2}^t e^{-\beta _2(t-\theta )}I(\theta )d\theta + \dfrac{\delta (\alpha +\delta )}{km}\int _{t-\tau _3}^t e^{-\beta _3(t-\theta )}D(\theta )d\theta ,\quad m\gg n\gg 1.\nonumber \\ \end{aligned}$$

Taking the time derivative of *Y*(*t*) yields

$$\begin{aligned} \dfrac{dY(t)}{dt}= & {} s_0-dH(t)-\beta _1 E(t)-\dfrac{\delta (n-1)}{n} I(t)-\dfrac{\delta (\alpha +\delta )(m-n)}{knm} D(t)\\{} & {} -\dfrac{\delta \mu (\alpha +\delta )}{k\alpha m}V(t)-\dfrac{pq}{a}Z(t)\\{} & {} -\dfrac{rc\delta (\alpha +\delta )}{k\alpha bm}W(t)-\dfrac{\beta _2\delta }{n}\int _{t-\tau _2}^t e^{-\beta _2(t-\theta )}I(\theta )d\theta - \dfrac{\beta _3\delta (\alpha +\delta )}{km}\\{} & {} \int _{t-\tau _3}^t e^{-\beta _3(t-\theta )}D(\theta )d\theta \\\le & {} s_0-\gamma _0Y(t), \end{aligned}$$

where $$\gamma _0=\min \left\{ d,\beta _1,\dfrac{\delta (n-1)}{n},\dfrac{(\alpha +\delta )(m-n)}{m},\mu ,q,c,\beta _2,\beta _3\right\} .$$

Hence, $\displaystyle \limsup _{t\rightarrow \infty }Y(t)\le \dfrac{s_0}{\gamma _0}:=M_1,$ implying that $\displaystyle \limsup _{t\rightarrow \infty }H(t)\le M_1,$ $\displaystyle \limsup _{t\rightarrow \infty }E(t)\le M_1,$ $\displaystyle \limsup _{t\rightarrow \infty }I(t)\le M_1,$ $\displaystyle \limsup _{t\rightarrow \infty }D(t)\le \frac{knM_1}{\delta }:=M_2,$ $\displaystyle \limsup _{t\rightarrow \infty }V(t)\le \frac{k\alpha mM_1}{\delta (\alpha +\delta )}:=M_3,$ $\displaystyle \limsup _{t\rightarrow \infty }W(t)\le \frac{k\alpha bmM_1}{r\delta (\alpha +\delta )}:=M_4,$ $\displaystyle \limsup _{t\rightarrow \infty }Z(t)\le \frac{aM_1}{p}:=M_5.$ This shows that the variables *H*, *E*,  *I*,  *D*,  *V*,  *W* and *Z* are uniformly bounded. This completes the proof. $\square$

Theorem 3.2 implies that omega limit sets of model (2.1) are contained in the following bounded feasible region:

$$\begin{aligned}{} & {} \Omega = \{(H,E,I,D,V,W,Z)\in {\mathbb {R}}_+^7: \Vert H\Vert _\infty ,\;\Vert E\Vert _\infty ,\; \Vert I\Vert _\infty \\{} & {} \quad \le M_1,\; \Vert D\Vert _\infty \le M_2,\; \Vert V\Vert _\infty \le M_3,\\{} & {} \qquad \qquad \qquad \qquad \Vert W\Vert _\infty \le M_4,\; \Vert Z\Vert _\infty \le M_5\}. \end{aligned}$$

It can be verified that the region $\Omega$ is positively invariant with respect model (2.1) and the system is well posed.

## Equilibria and threshold parameters

In this section, the biological feasible steady states of system (2.1) is investigated. Further, the virus basic reproduction number, the antibody immune response reproduction number, the CTL immune response reproduction number, the competitive CTL immune response reproduction number and the competitive antibody immune response reproduction number are also discussed. Let $P=({\check{H}},{\check{E}},{\check{I}},{\check{D}},{\check{V}},{\check{W}},{\check{Z}})$ be any feasible steady state of system (2.1). Then *P* satisfies the following algebraic equations system:

4.1 $$\begin{aligned} {\left\{ \begin{array}{ll} s_0-d{\check{H}}-f({\check{H}},{\check{V}}){\check{V}}=0,\\ (1-e^{-\beta _1\tau _1})f({\check{H}},{\check{V}}){\check{V}}-\beta _1{\check{E}}=0,\\ e^{-\beta _1\tau _1}f({\check{H}},{\check{V}}){\check{V}}-\delta {\check{I}}-p{\check{I}}{\check{Z}}=0,\\ ke^{-\beta _2\tau _2}{\check{I}} -(\alpha +\delta ){\check{D}}=0,\\ \alpha e^{-\beta _3\tau _3}{\check{D}}-\mu {\check{V}}-r{\check{V}}{\check{W}}=0,\\ b{\check{V}}{\check{W}}-c{\check{W}}=0,\\ a{\check{I}}{\check{Z}}-q{\check{Z}}=0. \end{array}\right. } \end{aligned}$$

Then, from system (4.1), it can be easily shown that the unique HBV-free equilibrium of system (2.1) is given by $P_0=(H_0,0,0,0,0,0,0),$ where $H_0=\dfrac{s_0}{d}.$ Further, $P_0$ always exists. It can be proven that the virus basic reproduction number of system (2.1) is given by

4.2 $$\begin{aligned} {\mathcal {R}}_0= \dfrac{k\alpha f\left( \dfrac{s_0}{d},0\right) }{\delta \mu (\alpha +\delta )}e^{-\beta _1\tau _1-\beta _2\tau _2 -\beta _3\tau _3}.\end{aligned}$$

Biologically speaking, ${\mathcal {R}}_0$ measures the average number of newly infected hepatocytes generated by a single virion at the beginning of the infection process in a completely susceptible population. Now, by writing ${\mathcal {R}}_0$ as

4.3 $$\begin{aligned} {\mathcal {R}}_0=\dfrac{\alpha e^{-\beta _3\tau _3}}{\mu }\cdot \dfrac{ke^{-\beta _2\tau _2}}{\alpha +\delta } \cdot \dfrac{e^{-\beta _1\tau _1}f\left( \dfrac{s_0}{d},0\right) }{\delta },\end{aligned}$$

we obtain the following significance interpretations. The average survival time of an infectious cell in the compartment *I* is $\frac{1}{\delta }.$ During this period, a virus-producing cell generates *k* HBV DNA-containing capsids per unit time. $\frac{\alpha }{\alpha +\delta }$ gives the amount of virions created from an intracellular HBV DNA-containing capsid during its survival duration. The fraction $e^{-\beta _1\tau _1}$ is the probability of surviving of hepatocytes infected from $t-\tau _1$ to *t*,  whereas the quotients $e^{-\beta _2\tau _2}$ and $e^{-\beta _3\tau _3}$ represent the probabilities of surviving the immature capsids from time $t-\tau _2$ to time *t*,  as well as the immature free virus particles from time $t-\tau _3$ to time *t*,  respectively. The average life expectancy of a free virus is given by $\frac{1}{\mu }$ and $f\left( \dfrac{s_0}{d},0 \right)$ denotes the value of the function *f* at the beginning of the HBV infection process in vivo. These relevant arguments infer that ${\mathcal {R}}_0$ represents the expected number of newly actively infected cells generated either by one exposed infected cell or one actively infected cell.

Now, from the sixth equation in (4.1), we have $(b{\check{V}}-c){\check{W}}=0.$ This gives two possible options, namely

4.4 $$\begin{aligned} {\check{V}}=\dfrac{c}{b} \quad \text {or}\quad {\check{W}}=0. \end{aligned}$$

Also, by the seventh equation in (4.1), we get $(a{\check{I}}-q){\check{Z}}=0.$ This also gives two possible options, namely

4.5 $$\begin{aligned} {\check{I}}=\dfrac{q}{a} \quad \text {or}\quad {\check{Z}}=0. \end{aligned}$$

Owing to (4.4) and (4.5), there are four cases.

First, consider that ${\check{W}}=0$ and ${\check{Z}}=0.$ In this case, equilibrium conditions in (4.1) are reduced to the following system

4.6 $$\begin{aligned} {\left\{ \begin{array}{ll} s_0-d{\check{H}}-f({\check{H}},{\check{V}}){\check{V}}=0,\\ (1-e^{-\beta _1\tau _1})f({\check{H}},{\check{V}}){\check{V}}-\beta _1{\check{E}}=0,\\ e^{-\beta _1\tau _1}f({\check{H}},{\check{V}}){\check{V}}-\delta {\check{I}}=0,\\ ke^{-\beta _2\tau _2}{\check{I}} -(\alpha +\delta ){\check{D}}=0,\\ \alpha e^{-\beta _3\tau _3}{\check{D}}-\mu {\check{V}}=0. \end{array}\right. } \end{aligned}$$

Then, from (4.6), we establish the following relationships

4.7 $$\begin{aligned}{} & {} f({\check{H}},{\check{V}}){\check{V}}=s_0-d{\check{H}},\quad {\check{E}}=\dfrac{(s_0-d{\check{H}})(1-e^{-\beta _1\tau _1})}{\beta _1},\quad {\check{I}}=\dfrac{(s_0-d{\check{H}})e^{-\beta _1\tau _1}}{\delta },\nonumber \\{} & {} {\check{D}}=\dfrac{k(s_0-d{\check{H}})e^{-\beta _1\tau _1-\beta _2\tau _2}}{\delta (\alpha +\delta )}\quad \text {and}\quad {\check{V}}=\dfrac{k\alpha (s_0-d{\check{H}})}{\delta \mu (\alpha +\delta )}e^{-\beta _1\tau _1-\beta _2\tau _2-\beta _3\tau _3}.\end{aligned}$$

Equations in (4.7) yield

4.8 $$\begin{aligned} f\left( {\check{H}},\dfrac{k\alpha (s_0-d{\check{H}})}{\delta \mu (\alpha +\delta )}e^{-\beta _1\tau _1-\beta _2\tau _2-\beta _3\tau _3}\right) =\dfrac{\delta \mu (\alpha +\delta )}{k\alpha }e^{\beta _1\tau _1+\beta _2\tau _2+\beta _3\tau _3}.\end{aligned}$$

Since ${\check{I}}$ denotes the number of infected hepatocytes, it is required that ${\check{I}}\ge 0.$ So, with this condition, it follows from the third term in (4.7) that ${\check{H}}\le \dfrac{s_0}{d}.$ Define the following function on $[0,s_0/d]$ as

4.9 $$\begin{aligned} F_1(H)=f\left( H,\dfrac{k\alpha (s_0-dH)}{\delta \mu (\alpha +\delta )}e^{-\beta _1\tau _1-\beta _2\tau _2-\beta _3\tau _3}\right) -\dfrac{\delta \mu (\alpha +\delta )}{k\alpha }e^{\beta _1\tau _1+\beta _2\tau _2+\beta _3\tau _3}.\end{aligned}$$

Based on the assumptions $(B_1)$-$(B_3),$ we find

$$\begin{aligned}{} & {} F_1(0)=-\dfrac{\delta \mu (\alpha +\delta )}{k\alpha }e^{\beta _1\tau _1+\beta _2\tau _2+\beta _3\tau _3}<0, \\{} & {} \quad F_1\left( \dfrac{s_0}{d}\right) =\dfrac{\delta \mu (\alpha +\delta )}{k\alpha }e^{\beta _1\tau _1 +\beta _2\tau _2+\beta _3\tau _3}({\mathcal {R}}_0-1), \end{aligned}$$

and

$$\begin{aligned} F'_1(H)=\dfrac{\partial f}{\partial H}-\dfrac{k\alpha d}{\delta \mu (\alpha +\delta )}e^{-\beta _1\tau _1-\beta _2\tau _2-\beta _3\tau _3}\dfrac{\partial f}{\partial V}>0. \end{aligned}$$

Clearly, we have $F_1\left( \dfrac{s_0}{d}\right) >0$ whenever ${\mathcal {R}}_0>1.$ Owing to $(B_2)$-$(B_3),$ we know that $F_1(H)$ is strictly monotonically nondecreasing function with respect to *H*. Thus, if ${\mathcal {R}}_0>1,$ there exists a unique root $H_1\in (0,s_0/d)$ such that $F_1(H_1)=0.$ Accordingly, we get a unique immune-free equilibrium $P_1=(H_1,E_1,I_1,D_1,V_1,0,0),$ where

$$\begin{aligned}{} & {} H_1\in (0,s_0/d),\quad E_1=\dfrac{(s_0-dH_1)(1-e^{-\beta _1\tau _1})}{\beta _1},\quad I_1=\dfrac{(s_0-dH_1)e^{-\beta _1\tau _1}}{\delta },\\{} & {} D_1=\dfrac{k(s_0-dH_1)e^{-\beta _1\tau _1-\beta _2\tau _2}}{\delta (\alpha +\delta )}\quad \text {and}\quad V_1=\dfrac{k\alpha (s_0-dH_1)}{\delta \mu (\alpha +\delta )}e^{-\beta _1\tau _1-\beta _2\tau _2-\beta _3\tau _3}. \end{aligned}$$

This means that in the absence of immune response, the equilibrium infection point $P_1$ would exist whenever ${\mathcal {R}}_0>1.$

Next, if ${\check{W}}\ne 0$ and ${\check{Z}}=0,$ then from (4.4), we have ${\check{V}}=\dfrac{c}{b}.$ By the first three equations in (4.6), we get

$$\begin{aligned} f\left( {\check{H}},\dfrac{c}{b}\right) =\dfrac{b(s_0-d{\check{H}})}{c}. \end{aligned}$$

Since, ${\check{W}}=\dfrac{kb\alpha (s_0-d{\check{H}})}{\delta (\alpha +\delta )rc}e^{-\beta _1\tau _1-\beta _2\tau _2-\beta _3\tau _3}-\dfrac{\mu }{r}\ge 0$ in term of biology, we have

$$\begin{aligned} {\check{H}}\le \dfrac{s_0}{d}-\dfrac{\delta \mu (\alpha +\delta )c}{dkb\alpha }e^{\beta _1\tau _1+\beta _2\tau _2+\beta _3\tau _3}. \end{aligned}$$

Now, we define the following function on the closed interval $\left[ 0,\dfrac{s_0}{d}-\dfrac{\delta \mu c(\alpha +\delta )}{dkb\alpha }e^{\beta _1\tau _1+\beta _2\tau _2+\beta _3\tau _3}\right]$ as

$$\begin{aligned} F_2(H)=f\left( H,\dfrac{c}{b}\right) -\dfrac{b(s_0-dH)}{c}. \end{aligned}$$

Then, with the aid of $(B_1)$-$(B_3),$ one has

$$\begin{aligned} F_2(0)=-\dfrac{bs_0}{c}<0 \quad \text {and}\quad F'_2(H)=\dfrac{\partial f}{\partial H}+\dfrac{bd}{c}>0. \end{aligned}$$

This implies that the function $F_2$ is strictly monotonically increasing with respect to *H*. Define the antibody immune response reproduction number for system (2.1) by

4.10 $$\begin{aligned} {\mathcal {R}}_1=\dfrac{b}{c}V_1=\dfrac{k\alpha b(s_0-dH_1)}{\delta \mu c(\alpha +\delta )}e^{-\beta _1\tau _1-\beta _2\tau _2-\beta _3\tau _3}.\end{aligned}$$

${\mathcal {R}}_1$ is the expected average number of antibody immune cells activated by virus when HBV infection is successful and CTL immune response is not yet established (Manna 2018; Miao et al. 2018). In the expression of ${\mathcal {R}}_1,$ *b* represents the activation rate of antibody immune response, and $\frac{1}{c}$ denotes the average life span of antibody immune cells and $V_1$ is the number of free virions at equilibrium point $P_1.$

Now, if ${\mathcal {R}}_1>1,$ then $V_1>\dfrac{c}{b}$ and $H_1<\dfrac{s_0}{d}-\dfrac{\delta \mu c(\alpha +\delta )}{dkb\alpha }e^{\beta _1\tau _1+\beta _2\tau _2+\beta _3\tau _3}.$ So, we have

$$\begin{aligned}{} & {} F_2\left( \dfrac{s_0}{d}-\dfrac{\delta \mu c(\alpha +\delta )}{dkb\alpha }e^{\beta _1\tau _1+\beta _2\tau _2+\beta _3\tau _3}\right) \\{} & {} =f\left( \dfrac{s_0}{d}-\dfrac{\delta \mu c(\alpha +\delta )}{dkb\alpha }e^{\beta _1\tau _1+\beta _2\tau _2+\beta _3\tau _3},\dfrac{c}{b}\right) -\dfrac{\delta \mu (\alpha +\delta )}{k\alpha }e^{\beta _1\tau _1+\beta _2\tau _2+\beta _3\tau _3},\\{} & {} =F_1\left( \dfrac{s_0}{d}-\dfrac{\delta \mu c(\alpha +\delta )}{dkb\alpha }e^{\beta _1\tau _1+\beta _2\tau _2+\beta _3\tau _3}\right) >F_1\left( H_1\right) =0\end{aligned}$$

This implies that when ${\mathcal {R}}_1>1,$ system (2.1) has a unique infection equilibrium with only antibody immune response $P_2=(H_2,E_2,I_2,D_2,V_2,W_2,0),$ where

$$\begin{aligned}{} & {} H_2\in \left( 0,\dfrac{s_0}{d}-\dfrac{\delta \mu c(\alpha +\delta )}{dkb\alpha }e^{\beta _1\tau _1+\beta _2\tau _2+\beta _3\tau _3}\right) ,\quad E_2=\dfrac{(s_0-d{H}_2)(1-e^{-\beta _1\tau _1})}{\beta _1},\\ {}{} & {} I_2=\dfrac{(s_0-dH_2)e^{-\beta _1\tau _1}}{\delta },\quad D_2=\dfrac{k(s_0-dH_2)}{\delta (\alpha +\delta )}e^{-\beta _1\tau _1-\beta _2\tau _2},\\{} & {} V_2=\dfrac{c}{b} \quad \text {and}\quad W_2=\dfrac{kb\alpha (s_0-dH_2)}{\delta (\alpha +\delta )rc}e^{-\beta _1\tau _1-\beta _2\tau _2 -\beta _3\tau _3}-\dfrac{\mu }{r}. \end{aligned}$$

Now, if ${\check{W}}=0$ and ${\check{Z}}\ne 0,$ then from (4.5), we have ${\check{I}}=\dfrac{q}{a},$ ${\check{E}}=\dfrac{(s_0-d{\check{H}})(1-e^{-\beta _1\tau _1})}{\beta _1},$

${\check{D}}=\dfrac{kqe^{-\beta _2\tau _2}}{a(\alpha +\delta )}$ and ${\check{V}}=\dfrac{k\alpha q}{a\mu (\alpha +\delta )}e^{-\beta _2\tau _2-\beta _3\tau _3}.$ By the first equation in (4.1), we get

4.11 $$\begin{aligned} f\left( {\check{H}},\dfrac{k\alpha q}{a\mu (\alpha +\delta )}e^{-\beta _2\tau _2 -\beta _3\tau _3}\right) =\dfrac{a\mu (\alpha +\delta )(s_0-d{\check{H}})}{k\alpha q}e^{\beta _2\tau _2+\beta _3\tau _3}.\end{aligned}$$

But, by the third equation in (4.6), the number of CTL immune cells gives

$$\begin{aligned} {\check{Z}}=\dfrac{a(s_0-d{\check{H}})e^{-\beta _1\tau _1}}{pq}-\dfrac{\delta }{p}\ge 0, \end{aligned}$$

in term of biology, which reads as ${\check{H}}\le \dfrac{s_0}{d}-\dfrac{\delta qe^{\beta _1\tau _1}}{ad}.$ Define the following function on $\left[ 0,\dfrac{s_0}{d}-\dfrac{\delta qe^{\beta _1\tau _1}}{ad}\right]$ as

$$\begin{aligned} F_3(H)= f\left( {H},\dfrac{k\alpha q}{a\mu (\alpha +\delta )}e^{-\beta _2\tau _2-\beta _3\tau _3}\right) -\dfrac{a\mu (\alpha +\delta )(s_0-d{H})}{k\alpha q}e^{\beta _2\tau _2+\beta _3\tau _3}. \end{aligned}$$

Then, thanks to $(B_1)$-$(B_3),$ we have

$$\begin{aligned}{} & {} F_3(0)=-\dfrac{a\mu (\alpha +\delta )s_0}{k\alpha q}e^{\beta _2\tau _2+\beta _3\tau _3}<0\quad \text {and}\quad \\{} & {} \quad F'_3(H)=\dfrac{\partial f}{\partial H}+\dfrac{ad\mu (\alpha +\delta )}{k\alpha q}e^{\beta _2\tau _2+\beta _3\tau _3}>0. \end{aligned}$$

Now, we define the CTL immune defense reproduction number as

4.12 $$\begin{aligned} {\mathcal {R}}_2=\dfrac{a}{q}I_1=\dfrac{a(s_0-dH_1)e^{-\beta _1\tau _1}}{\delta q}. \end{aligned}$$

Then, ${\mathcal {R}}_2$ denotes the expected average number of CTL immune defense activated by both exposed cells and infected hepatocytes when HBV infection is successful and antibody immune response is not yet established. In the expression of ${\mathcal {R}}_2,$ *a* represents the activation rate of CTL immune response, $\frac{1}{q}$ denotes the average life span of CTL immune cells and $I_1$ is the number of infected hepatocytes at equilibrium $P_1.$

It is obvious that if ${\mathcal {R}}_2>1,$ then $I_1>\dfrac{q}{a}$ and $H_1<\dfrac{s_0}{d}-\dfrac{\delta qe^{\beta _1\tau _1}}{ad}.$ So, we have

$$\begin{aligned} F_3\left( \dfrac{s_0}{d}-\dfrac{\delta qe^{\beta _1\tau _1}}{ad}\right)= & {} f\left( \dfrac{s_0}{d}-\dfrac{\delta qe^{\beta _1\tau _1}}{ad},\dfrac{k\alpha q}{a\mu (\alpha +\delta )}e^{-\beta _2\tau _2 -\beta _3\tau _3}\right) \\{} & {} -\dfrac{\delta \mu (\alpha +\delta )}{k\alpha }e^{\beta _1\tau _1+\beta _2\tau _2+\beta _3\tau _3},\\{} & {} >f(H_1,V_1)-\dfrac{\delta \mu (\alpha +\delta )}{k\alpha }e^{\beta _1\tau _1+\beta _2\tau _2 +\beta _3\tau _3}=0. \end{aligned}$$

Accordingly, when ${\mathcal {R}}_2>1,$ system (2.1) has a unique infection equilibrium point with only CTL immune defense $P_3=(H_3,E_3,I_3,D_3,V_3,0,Z_3),$ where

$$\begin{aligned}{} & {} H_3\in \left( 0,\dfrac{s_0}{d}-\dfrac{\delta qe^{\beta _1\tau _1}}{ad}\right) ,\quad E_3=\dfrac{(s_0-d{H}_3)(1-e^{-\beta _1\tau _1})}{\beta _1},\quad I_3=\dfrac{q}{a},\\{} & {} D_3=\dfrac{kq}{a(\alpha +\delta )}e^{-\beta _2\tau _2},\; V_3=\dfrac{k\alpha q}{a\mu (\alpha +\delta )}e^{-\beta _2\tau _2-\beta _3\tau _3}\; \text {and}\; \\{} & {} Z_3=\dfrac{a(s_0-dH_3)(\beta _1e^{-\beta _1\tau _1}}{pq}-\dfrac{\delta }{p}. \end{aligned}$$

Finally, if ${\check{W}}\ne 0$ and ${\check{Z}}\ne 0,$ then from (4.6), (4.4) and (4.5), we have ${\check{E}}=\dfrac{(s_0-d{\check{H}})(1-e^{-\beta _1\tau _1})}{\beta _1},$ ${\check{D}}=\dfrac{kq}{a(\alpha +\delta )}e^{-\beta _2\tau _2},$ ${\check{I}}=\dfrac{q}{a},$ ${\check{V}}=\dfrac{c}{b}$ and

$$\begin{aligned} f\left( {\check{H}},\dfrac{c}{b}\right) =\dfrac{b}{c}(s_0-d{\check{H}}). \end{aligned}$$

Again, from the third equation in (4.6), the number of CTL immune cells is

$$\begin{aligned} {\check{Z}}=\dfrac{a(s_0-d{\check{H}})e^{-\beta _1\tau _1}}{pq}-\dfrac{\delta }{p}\ge 0 \end{aligned}$$

in term of biology, which leads to ${\check{H}}\le \dfrac{s_0}{d}-\dfrac{\delta qe^{\beta _1\tau _1}}{ad}.$ Define the following function on $\left[ 0,\dfrac{s_0}{d}-\dfrac{\delta qe^{\beta _1\tau _1}}{ad}\right]$ as

$$\begin{aligned} F_4(H)= f\left( {H},\dfrac{c}{b}\right) -\dfrac{b}{c}(s_0-d{H}). \end{aligned}$$

Then, according to $(B_1)$-$(B_3),$ we have $F_4(0)=-\dfrac{bs_0}{c}<0$ and $F'_4(H)=\dfrac{\partial f}{\partial H}+\dfrac{bd}{c}>0.$

Defined the competitive CTL immune response reproduction number for system (2.1) by

4.13 $$\begin{aligned} {\mathcal {R}}_3=\dfrac{aI_2}{q}=\dfrac{a(s_0-dH_2)e^{-\beta _1\tau _1}}{\delta q}. \end{aligned}$$

This threshold number ${\mathcal {R}}_3$ denotes the expected average number of CTL immune cells activated by both exposed cells and infected hepatocytes when antibody immune defense has already been established. Consequently, if ${\mathcal {R}}_3>1,$ then we get $I>\dfrac{q}{a},$ $H_2<\dfrac{s_0}{d}-\dfrac{\delta qe^{\beta _1\tau _1}}{ad}$ and

$$\begin{aligned} F_4\left( \dfrac{s_0}{d}-\dfrac{\delta qe^{\beta _1\tau _1}}{ad}\right)= & {} f\left( \dfrac{s_0}{d}-\dfrac{\delta qe^{\beta _1\tau _1}}{ad},\dfrac{c}{b}\right) -\dfrac{\delta bq}{ac}e^{\beta _1\tau _1}\\= & {} F_2\left( \dfrac{s_0}{d}-\dfrac{\delta qe^{\beta _1\tau _1}}{ad}\right) >F_2(H_2)=0.\end{aligned}$$

Accordingly, there exists a unique root $H_4\in \left( 0,\dfrac{s_0}{d}-\dfrac{\delta qe^{\beta _1\tau _1}}{ad}\right) ,$ such that $F_4(H_4)=0.$ From the fifth equation in (4.1), we get $W_4=\dfrac{k\alpha bq}{arc(\alpha +\delta )}e^{-\beta _2\tau _2-\beta _3\tau _3}-\dfrac{\mu }{r}=\dfrac{\mu }{r}({\mathcal {R}}_4-1),$ where ${\mathcal {R}}_4$ represents the competitive antibody immune response reproduction number and defined by

4.14 $$\begin{aligned} {\mathcal {R}}_4=\dfrac{bV_3}{c}=\dfrac{k\alpha bq}{a\mu c(\alpha +\delta )}e^{-\beta _2\tau _2-\beta _3\tau _3}. \end{aligned}$$

This threshold number ${\mathcal {R}}_4$ denotes the expected average number of B cells activated by free virions whenever the CTL immune defense has already been established (Manna 2018; Miao et al. 2018).

If $${\mathcal {R}}_3>1$$ and $${\mathcal {R}}_4>1,$$ system (2.1) admits a unique infection equilibrium point in presence of adaptive immune responses characterized by antibody and CTL immune responses $$P_4=(H_4,$$ $$E_4,$$ $$I_4,$$ $$D_4,$$ $$V_4,$$ $$W_4,$$ $$Z_4),$$ where

$$\begin{aligned}{} & {} H_4\in \left( 0,\dfrac{s_0}{d}-\dfrac{\delta qe^{\beta _1\tau _1}}{ad}\right) ,\quad E_4=\dfrac{(s_0-d{H}_4)(1-e^{-\beta _1\tau _1})}{\beta _1},\quad I_4=\dfrac{q}{a},\\{} & {} D_4=\dfrac{kq}{a(\alpha +\delta )}e^{-\beta _2\tau _2}, \quad V_4=\dfrac{c}{b},\quad W_4=\dfrac{\mu }{r}({\mathcal {R}}_4-1)\quad \text {and} \\{} & {} \quad Z_4=\dfrac{a(s_0-dH_4)e^{-\beta _1\tau _1}}{pq}-\dfrac{\delta }{p}. \end{aligned}$$

The above investigations can be summarized in the following result.

### Theorem 4.1

System (2.1) has a unique infection-free equilibrium $P_0 = (s_0/d,0, 0, 0, 0, 0, 0)$ whenever ${\mathcal {R}}_0 \le 1.$ When ${\mathcal {R}}_0>1,$ the system (2.1) admits five equilibria including the equilibrium point $P_0.$ Moreover, for ${\mathcal {R}}_0>1,$

($$\hbox {i}_1$$)
the unique immune-free equilibrium $P_1=(H_1,E_1,I_1,D_1,V_1,0,0),$ where $H_1\in (0,s_0/d)$ and $H_1,$ $E_1,$ $I_1,$ $D_1,$ $V_1>0,$ always exists;
($$\hbox {i}_2$$)
the infection equilibrium with only antibody immune defense $P_2=(H_2,E_2,I_2,D_2,V_2,W_2,0),$ where $H_2\in \left( 0,\dfrac{s_0}{d}-\dfrac{\delta \mu c(\alpha +\delta )}{dkb\alpha }e^{\beta _1\tau _1+\beta _2\tau _2+\beta _3\tau _3}\right)$ and $E_2,I_2,D_2,V_2,W_2>0,$ exists and is unique when ${\mathcal {R}}_1>1;$
($$\hbox {i}_3$$)
the infection equilibrium with only CTL immune response $P_3=(H_3,E_3,I_3,D_3,V_3,0,Z_3),$ where $H_3\in \left( 0,\dfrac{s_0}{d}-\dfrac{\delta qe^{\beta _1\tau _1}}{ad}\right)$ and $E_3,I_3,D_3,V_3,Z_3>0,$ exists and is unique when ${\mathcal {R}}_2>1;$
($$\hbox {i}_4$$)
the interior infection equilibrium with both antibody and CTL immune response $$P_4=(H_4,$$ $$E_4,$$ $$I_4,$$ $$D_4,$$ $$V_4,$$ $$W_4,$$ $$Z_4),$$ where $$H_4\in \left( 0,\dfrac{s_0}{d}-\dfrac{\delta qe^{\beta _1\tau _1}}{ad}\right) $$ and $$E_4,I_4,D_4,V_4,W_4,Z_4>0,$$ exists and is unique when $${\mathcal {R}}_1>1,$$ $${\mathcal {R}}_2>1,$$ $${\mathcal {R}}_3>1$$ and $${\mathcal {R}}_4>1.$$

## Stability analysis of constants equilibria

In this section, we discuss the stability properties of the five constants equilibria $P_0,$ $P_1,$ $P_2,$ $P_3$ and $P_4,$ secured by Theorem 4.1, of the proposed model (2.1), by analyzing the corresponding characteristic equation and by using the Lyapunov method. To do this, we introduce the following assumption:

$$\begin{aligned} (B_4)\qquad \qquad \left( 1-\dfrac{f(H,V)}{f(H,V_i)}\right) \left( \dfrac{f(H,V_i)}{f(H,V)}-\dfrac{V}{V_i}\right) \le 0, \; \text {for all}\; H, V>0, \end{aligned}$$

where $V_i$ is the free virion components of the steady state $P_i,$ $i=1,2,3,4.$

Now, let $P^*=(H^*,E^*,I^*,D^*,V^*,W^*,Z^*)$ be any steady state of system (2.1) and consider the perturbation related to the components of the steady state $P^*$ as follows

$$\begin{aligned}{} & {} y_1(t)=H(t)-H^*,\quad y_2(t)=E(t)-E^*,\quad y_3(t)=I(t)-I^*,\;\;\,\quad \\{} & {} y_4(t)=D(t)-D^*,\quad y_5(t)=V(t)-V^*,\quad y_6(t)=W(t)-W^*,\quad y_7(t)=Z(t)-Z^*. \end{aligned}$$

Linearizing system (2.1) at the equilibrium point $P^*,$ we get the following linearized system:

5.1 $$\begin{aligned} \dfrac{dy}{dt}={\mathcal {G}}_{1}y(t)+{\mathcal {G}}_{2}y(t-\tau _1) +{\mathcal {G}}_{3}y(t-\tau _2)+{\mathcal {G}}_{4}y(t-\tau _3), \end{aligned}$$

where

$$\begin{aligned}{} & {} {\mathcal {G}}_{1}=\left( \begin{array}{ccccccc} -d-\frac{\partial f}{\partial H}V^*&{}0&{}0&{}0&{}-\frac{\partial f}{\partial V}V^*-f(H^*,V^*)&{}0&{}0\\ \frac{\partial f}{\partial H}V^*&{}-\beta _1&{}0&{}0&{}\frac{\partial f}{\partial V}V^*+f(H^*,V^*)&{}0&{}0\\ 0&{}0&{}-\delta -pZ^*&{}0&{}0&{}0&{}-pI^*\\ 0&{}0&{}0&{}-\alpha -\delta &{}0&{}0&{}0\\ 0&{}0&{}0&{}0&{}-\mu -rW^*&{}-rV^*&{}0\\ 0&{}0&{}0&{}0&{}bW^*&{}bV^*-c&{}0\\ 0&{}0&{}aZ^*&{}0&{}0&{}0&{}aI^*-q \end{array}\right) ,\\{} & {} {\mathcal {G}}_{2}=\left( \begin{array}{ccccccc} 0&{}0&{}0&{}0&{}0&{}0&{}0\\ -e^{-\beta _1\tau _1}\frac{\partial f}{\partial H}V^*&{}0&{}0&{}0&{}-e^{-\beta _1\tau _1}\left( \frac{\partial f}{\partial V}V^*+f(H^*,V^*)\right) &{}0&{}0\\ e^{-\beta _1\tau _1}\frac{\partial f}{\partial H}V^*&{}0&{}0&{}0&{}e^{-\beta _1\tau _1}\left( \frac{\partial f}{\partial V}V^*+f(H^*,V^*)\right) &{}0&{}0\\ 0&{}0&{}0&{}0&{}0&{}0&{}0\\ 0&{}0&{}0&{}0&{}0&{}0&{}0\\ 0&{}0&{}0&{}0&{}0&{}0&{}0\\ 0&{}0&{}0&{}0&{}0&{}0&{}0 \end{array}\right) ,\\{} & {} {\mathcal {G}}_{3}=\left( \begin{array}{ccccccc} 0&{}0&{}0&{}0&{}0&{}0&{}0\\ 0&{}0&{}0&{}0&{}0&{}0&{}0\\ 0&{}0&{}0&{}0&{}0&{}0&{}0\\ 0&{}0&{}ke^{-\beta _2\tau _2}&{}0&{}0&{}0&{}0\\ 0&{}0&{}0&{}0&{}0&{}0&{}0\\ 0&{}0&{}0&{}0&{}0&{}0&{}0\\ 0&{}0&{}0&{}0&{}0&{}0&{}0 \end{array}\right) ,\qquad {\mathcal {G}}_{4}=\left( \begin{array}{ccccccc} 0&{}0&{}0&{}0&{}0&{}0&{}0\\ 0&{}0&{}0&{}0&{}0&{}0&{}0\\ 0&{}0&{}0&{}0&{}0&{}0&{}0\\ 0&{}0&{}0&{}0&{}0&{}0&{}0\\ 0&{}0&{}0&{}\alpha e^{-\beta _3\tau _3}&{}0&{}0&{}0\\ 0&{}0&{}0&{}0&{}0&{}0&{}0\\ 0&{}0&{}0&{}0&{}0&{}0&{}0 \end{array}\right) .\\{} & {} \text {and}\quad y=(y_1,y_2,y_3,y_4,y_5,y_6,y_7)^T. \end{aligned}$$

It is worth mentioning that the partial derivatives $\frac{\partial f}{\partial H}$ and $\frac{\partial f}{\partial V}$ in the first two matrices ${\mathcal {G}}_{1}$ and ${\mathcal {G}}_{2}$ are evaluated at the steady state $P^*.$ Hence, the characteristic equation of model (2.1) at the equilibrium $P^*$ is

$$\begin{aligned} \text {det}(-\lambda {\mathbb {I}}_7+{\mathcal {G}}_{1}+{\mathcal {G}}_{2}e^{-\lambda \tau _1}+{\mathcal {G}}_{3}e^{-\lambda \tau _2}+{\mathcal {G}}_{4}e^{-\lambda \tau _3})=0. \end{aligned}$$

### Stability of infection-free equilibrium $P_0$

#### Local stability of infection-free equilibrium

In this section, we investigate the local stability of infection-free equilibrium, $P_0,$ of model (2.1), by analyzing the corresponding characteristic equation. We have the following result.

##### Lemma 5.1

The infection-free equilibrium $P_0 =(s_0/d, 0, 0, 0, 0, 0, 0)$ of model (2.1) is locally asymptotically stable for any time delays $\tau _1,\tau _2,\tau _3\ge 0$ whenever ${\mathcal {R}}_0 <1$ and unstable when ${\mathcal {R}}_0>1,$

##### Proof

From system (5.1), by calculating, we see that, the stability of the equilibrium point, $P_0,$ is investigated via the roots of the following characteristic equation representing the corresponding linearized system of model (2.1) at the steady state $P_0$

5.2 $$\begin{aligned} (\lambda +d)(\lambda +c)(\lambda +q)(\lambda +\beta _1)\left[ \lambda ^3+a_2\lambda ^2+a_1\lambda +a_0+b_0e^{{-\lambda (\tau _1+\tau _2+\tau _3)}}\right] =0, \end{aligned}$$

where $$a_2=2\delta +\alpha +\mu ,$$ $$a_1=(\alpha +\delta )(\mu +\delta )+\delta \mu ,$$ $$a_0=\delta \mu (\alpha +\delta ),$$ $$b_0=-k\alpha f\left( \frac{s_0}{d},0\right) e^{-\beta _1\tau _1-\beta _2\tau _2-\beta _3\tau _3}.$$

It is obvious that, the characteristic equation (5.2) always admits the reals roots $\lambda _1=-d<0,$ $\lambda _2=-c<0,$ $\lambda _3=-q<0,$ $\lambda _4=-\beta _1<0,$ and all other roots of that equation (5.2) are given by the following equation:

5.3 $$\begin{aligned} h(\lambda )=: \lambda ^3+a_2\lambda ^2+a_1\lambda +a_0+b_0e^{{-\lambda (\tau _1+\tau _2+\tau _3)}}=0. \end{aligned}$$

If ${\mathcal {R}}_0 > 1,$ then, it can be shown that for $\lambda$ real,

$$\begin{aligned} h(0)=\delta \mu (\alpha +\delta )(1-{\mathcal {R}}_0)<0\quad \text {and}\quad \displaystyle \lim _{\lambda \rightarrow +\infty }h(\lambda )=+\infty . \end{aligned}$$

Thus, equation (5.3) has a positive real root. Consequently, there exists a characteristic root $\lambda$ with positive real part of (5.3) and therefore, if ${\mathcal {R}}_0 > 1,$ the infection-free equilibrium $P_0=\left( {s_0}/{d},0,0,0,0,0,0\right)$ is unstable.

Now, assume that ${\mathcal {R}}_0<1$ and let $\tau _0=\tau _1+\tau _2+\tau _3.$ Then if $i\omega$ (with $\omega >0$) is a solution to (5.3), separating real and imaginary parts yields

5.4 $$\begin{aligned} {\left\{ \begin{array}{ll} a_2\omega ^2-a_0=b_0\cos \omega \tau _0,\\ -\omega ^3+a_1\omega =b_0\sin \omega \tau _0. \end{array}\right. } \end{aligned}$$

From (5.4), we get the following equation

5.5 $$\begin{aligned} \omega ^6+(a_2^2-2a_1)\omega ^4+(a_1^2-2a_0a_2)\omega ^2+a_0^2-b_0^2=0.\end{aligned}$$

Setting $z=\omega ^2$ then equation (5.5) becomes

5.6 $$\begin{aligned} z^3+c_2z^2+c_1z+c_0=0, \end{aligned}$$

where

$$c_2=a_2^2-2a_1=(\alpha +\delta )^2+\mu ^2+\delta ^2>0,$$   

$$c_1=a_1^2-2a_0a_2=(\mu ^2+\delta ^2)(\alpha +\delta )^2+\delta ^2\mu ^2>0,$$

$$c_0=a_0^2-b_0^2=\delta ^2\mu ^2(\alpha +\delta )^2(1-{\mathcal {R}}_0^2)>0,$$

$$c_1c_2-c_0=(\mu ^2+\delta ^2)(\alpha +\delta )^4+(\mu ^2+\delta ^2)\left[ (\mu ^2+\delta ^2)(\alpha +\delta )^2+\delta ^2\mu ^2\right] +\delta ^2\mu ^2(\alpha +\delta )^2{\mathcal {R}}_0^2>0.$$

Hence, by the Routh-Hurwitz Theorem (Gradshteyn and Ryzhik 2000), if ${\mathcal {R}}_0<1,$ equation (5.6) has no positive roots. It is easy to show that $P_0$ is locally asymptotically stable when $\tau _1=\tau _2=\tau _3=0,$ Consequently, if ${\mathcal {R}}_0<1,$ the infection-free equilibrium $P_0$ is locally asymptotically stable for all $\tau _1,\tau _2,\tau _3\ge 0.$ $\square$

##### Remark 5.2

We note that the local asymptotic stability of the infection-free equilibrium $P_0$ could also be obtained by using a contradiction argument.

Indeed, for $\tau _1,\tau _2,\tau _3\ge 0,$ by computation, equation (5.3) becomes

$$\begin{aligned} 1={\mathcal {R}}_0\dfrac{\delta }{\lambda +\delta }\dfrac{\mu }{\lambda +\mu }\dfrac{\alpha +\delta }{\lambda +\alpha +\delta }e^{-\lambda (\tau _1+\tau _2+\tau _3)}. \end{aligned}$$

If $\lambda$ is a root of equation (5.3) with $\text {Re}\lambda \ge 0$ and ${\mathcal {R}}_0<1,$ then, observe

$$\begin{aligned} \left| \dfrac{\delta }{\lambda +\delta }\right|<1,\quad \left| \dfrac{\mu }{\lambda +\mu }\right|<1,\quad \left| \dfrac{\alpha +\delta }{\lambda +\alpha +\delta }\right|<1,\quad \left| e^{-\lambda (\tau _1+\tau _2+\tau _3)}\right| <1, \end{aligned}$$

which infers that

$$\begin{aligned} \left| {\mathcal {R}}_0\dfrac{\delta }{\lambda +\delta }\dfrac{\mu }{\lambda +\mu }\dfrac{\alpha +\delta }{\lambda +\alpha +\delta }e^{-\lambda (\tau _1+\tau _2+\tau _3)}\right| <1. \end{aligned}$$

This is a contradiction. Thus, all roots of equation (5.3) have no positive real parts. Accordingly, if ${\mathcal {R}}_0<1,$ the infection-free equilibrium $P_0$ is locally asymptotically stable for all $\tau _1,\tau _2,\tau _3\ge 0.$ $\square$

#### Global asymptotic stability of the equilibrium $P_0$

In this section, by constructing a suitable Lyapunov functional, we discuss the global stability of the infection-free equilibrium $P_0.$

##### Theorem 5.3

The infection-free equilibrium $P_0$ of model (2.1) is globally asymptotically stable in $\Omega$ if ${\mathcal {R}}_0\le 1.$

##### Proof

Let *H*(*t*), *E*(*t*), *I*(*t*), *D*(*t*), *V*(*t*), *W*(*t*), *Z*(*t*) be any arbitrary positive solution of system (2.1). Recall that $H_0={s_0}/{d}.$ We define a Lyapunov functional ${\widetilde{L}}_0(t)$ as

$$\begin{aligned}{} & {} {\widetilde{L}}_0(t)=H(t)-H_0-\int _{H_0}^{H(t)}\dfrac{f(H_0,0)}{f(s,0)}ds+e^{\beta _1\tau _1}I(t)+ \dfrac{\delta }{k}e^{\beta _1\tau _1+\beta _2\tau _2}D(t) \\{} & {} \qquad \qquad + \dfrac{\delta (\alpha +\delta )}{k\alpha }e^{\beta _1\tau _1+\beta _2\tau _2+\beta _3\tau _3}V(t)+ \dfrac{r\delta (\alpha +\delta )}{kb\alpha }e^{\beta _1\tau _1+\beta _2\tau _2+\beta _3\tau _3}W(t) \\{} & {} \qquad \qquad + \dfrac{p}{a}e^{\beta _1\tau _1}Z(t)+ \displaystyle \delta e^{\beta _1\tau _1}\int _{t-\tau _2}^tI(\theta )d\theta + \dfrac{\delta (\alpha +\delta )}{k}e^{\beta _1\tau _1+\beta _2\tau _2}\displaystyle \int _{t-\tau _3}^tD(\theta )d\theta \\{} & {} \qquad \qquad + \displaystyle \int _{t-\tau _1}^tf(H(\theta ), V(\theta ))V (\theta )d\theta . \end{aligned}$$

For the sake of notational convenience, we represent $\chi =\chi (t)$ and $\chi _{\tau _i}=\chi (t-\tau _i),$ for $i=1,2,3$ and $\chi \in \{H,E,I,D,V,W,Z\}.$ Let ${\mathbb {G}}_0(y)=\displaystyle y-H_0-\int _{H_0}^{y}\dfrac{f(H_0,0)}{f(s,0)}ds.$ Then, the function ${\mathbb {G}}_0$ is nonnegative for all $y>0.$ Indeed, if $y\le H_0,$ then with the aid of assumption $(B_2),$ we obtain

$$\begin{aligned} \displaystyle \int _{H_0}^{y}\dfrac{f(H_0,0)}{f(s,0)}ds\le \int _{H_0}^{y}\dfrac{f(H_0,0)}{f(H_0,0)}ds=y-H_0. \end{aligned}$$

If $y\ge H_0$ is valid, we again obtain the same above inequality. Further, ${\mathbb {G}}_0(y)=0$ if and only if $y=H_0.$ Therefore, ${\mathbb {G}}_0(y)\ge 0$ for all $y>0.$ Thus, it is clear that the functional ${\widetilde{L}}_0(t)$ is nonnegative definite with respect to $P_0.$ Now, by computing the time derivative of ${\widetilde{L}}_0(t)$ along the solution of system (2.1), we have

$$\begin{aligned} \dfrac{d{\widetilde{L}}_0(t)}{dt}= & {} \displaystyle dH_0\left( 1-\dfrac{H}{H_0}\right) \left( 1-\dfrac{f(H_0,0)}{f(H,0)}\right) + \dfrac{\delta (\alpha +\delta )}{k\alpha }e^{\beta _1\tau _1+\beta _2\tau _2+\beta _3\tau _3}\\{} & {} \times V\left( \dfrac{f(H,V)}{f(H,0)}{\mathcal {R}}_0-1\right) - \dfrac{rc\delta (\alpha +\delta )}{kb\alpha }e^{\beta _1\tau _1+\beta _2\tau _2+\beta _3\tau _3}W- \dfrac{pq}{a}e^{\beta _1\tau _1}Z. \end{aligned}$$

With the aid of $(B_2)$ and $(B_3),$ one has

5.7 $$\begin{aligned} f(H,V)\le f(H,0),\quad \text {for all}\quad H\ge 0, V\ge 0.\end{aligned}$$

Thus

$$\begin{aligned} \dfrac{d{\widetilde{L}}_0(t)}{dt}\le & {} \displaystyle dH_0\left( 1-\dfrac{H}{H_0}\right) \left( 1-\dfrac{f(H_0,0)}{f(H,0)}\right) + \dfrac{\delta (\alpha +\delta )}{k\alpha }e^{\beta _1\tau _1+\beta _2\tau _2+\beta _3\tau _3}V\left( {\mathcal {R}}_0-1\right) \\{} & {} - \dfrac{rc\delta (\alpha +\delta )}{kb\alpha }e^{\beta _1\tau _1+\beta _2\tau _2+\beta _3\tau _3}W- \dfrac{pq}{a}e^{\beta _1\tau _1}Z \end{aligned}$$

Since *f*(*H*, *V*) is a strictly monotonically nondecreasing function with respect to *H*,  by assumption $(B_2),$ it is easy to show that assumption $(B_2)$ ultimately gives rise to the following inequality:

$$\begin{aligned} \left( 1-\dfrac{H}{H_0}\right) \left( 1-\dfrac{f(H_0,0)}{f(H,0)}\right) \le 0. \end{aligned}$$

Clearly, condition ${\mathcal {R}}_0\le 1$ underwrites $\frac{d {\widetilde{L}}_0(t)}{dt}\le 0,$ for all $H, I, D, V, W, Z \ge 0,$ and $\frac{d {\widetilde{L}}_0(t)}{dt}=0$ is satisfied if and only if $H=H_0,$ $I=0,$ $D= 0,$ $V=0,$ $W=0$ and $Z=0.$ Hence, ${\widetilde{L}}_0(t)$ is a Lyapunov function on $\Omega .$ Accordingly, by LaSalle’s invariance principle (Hale and Verduyn Lunel 1993, Theorem 5.3.1), it follows that

5.8 $$\begin{aligned} \displaystyle \lim _{t\rightarrow \infty }(H,I,D,V,W,Z)=\left( H_0,0,0,0,0,0\right) . \end{aligned}$$

From (5.8), we have $\limsup _{t\rightarrow \infty }H=H_0$ and $\limsup _{t\rightarrow \infty }V=0.$ This means that for sufficiently small $\epsilon >0,$ there exist constants $N_1>0$ and $N_2>0$ such that $\limsup _{t\rightarrow \infty }H\le H_0+\epsilon ,$ for all $t>N_1$ and $\limsup _{t\rightarrow \infty }V\le \epsilon ,$ for all $t>N_2.$ So, from the second equation of system (2.1) and assumption $(B_2)$, it follows that for $t>\max \{N_1,N_2\},$

5.9 $$\begin{aligned} E^\infty =\limsup _{t\rightarrow \infty }E\le \dfrac{f(H_0+\epsilon ,\epsilon )\epsilon }{\beta _1}, \end{aligned}$$

so that, by setting $\epsilon \rightarrow 0$ in (5.9), we obtain

5.10 $$\begin{aligned} E^\infty =\limsup _{t\rightarrow \infty }E\le 0. \end{aligned}$$

Also, from (5.8), we have $\liminf _{t\rightarrow \infty }H=H_0$ and $\liminf _{t\rightarrow \infty }V=0.$ Hence, by employing a similar argument as above, it can be shown that

5.11 $$\begin{aligned} E_\infty =\liminf _{t\rightarrow \infty }E\ge 0. \end{aligned}$$

Then, from (5.10) and (5.11), we get

$$\begin{aligned} E^\infty \le 0\le E_\infty , \end{aligned}$$

implying that

5.12 $$\begin{aligned} \lim _{t\rightarrow \infty }E=0. \end{aligned}$$

Hence, we obtain from (5.8) and (5.12) that

$$\begin{aligned} \lim _{t\rightarrow \infty }(H,E,I,D,V,W,Z)=\left( H_0,0,0,0,0,0,0\right) . \end{aligned}$$

Furthermore, $\Omega$ is an invariant and attracting set of ${\mathbb {R}}^7_+.$ It follows that the largest compact invariant subset in $\left\{ (H,E,I,D,V,W,Z)\in \Omega :\; \frac{d{\widetilde{L}}_0}{dt}=0\right\}$ is the singleton $\{{\mathcal {P}}_0\}.$ So, by LaSalle’s invariance Principle (Hale and Verduyn Lunel 1993, Theorem 5.3.1), it follows that every solution of system (2.1) approaches the infection-free equilibrium $P_0$ as $t\rightarrow \infty$ whenever ${\mathcal {R}}_0\le 1.$ That is, the infection-free equilibrium $P_0$ is globally asymptotically stable if ${\mathcal {R}}_0\le 1.$ This completes the proof. $\square$

The epidemiological implication of Theorem 5.3 is that if the threshold quantity ${\mathcal {R}}_0$ has a value less than unity, then the free virions will be cleared from a body even in the absence of adaptive immunity which is represented by antibodies and CTLs. Moreover, we expect that by adding multi-time delays and the mortalities during the three time delays could contribute to obtain the condition ${\mathcal {R}}_0\le 1.$ Thus, for the delayed model (2.1), the condition ${\mathcal {R}}_0\le 1$ is necessary and sufficient for infection elimination.

In the sequel we will need the following function defined on ${\mathbb {R}}_+^*$: $\widetilde{{\mathcal {Q}}}(\xi )=\xi -1-\ln \xi .$ Obviously, $\widetilde{{\mathcal {Q}}}(\xi )\ge 0$ for all $\xi \in {\mathbb {R}}_+^*$ and $\widetilde{{\mathcal {Q}}}(\xi )=0$ if and only if $\xi =1.$

### Global asymptotic stability of equilibrium $P_1$

In this section, by constructing a crafty Lyapunov functional, we investigate the global asymptotic stability of the immune-free equilibrium $P_1.$ The following result is established.

#### Theorem 5.4

Let requirement $(B_4)$ and condition ${\mathcal {R}}_0>1$ hold. Then if ${\mathcal {R}}_1\le 1$ and ${\mathcal {R}}_2\le 1,$ the immune-free equilibrium $P_1$ of the delayed model (2.1) is globally asymptotically stable and it is unstable whenever ${\mathcal {R}}_1>1$ or ${\mathcal {R}}_2>1.$

#### Proof

Let (*H*(*t*), *E*(*t*), *I*(*t*), *D*(*t*), *V*(*t*), *W*(*t*), *Z*(*t*)) be any arbitrary positive solution of system (2.1). Define the following Lyapunov function

$$\begin{aligned} {\widetilde{L}}_1(t)= & {} \displaystyle H-H_1-\int _{H_1}^{H}\dfrac{f(H_1,V_1)}{f(s,V_1)}ds +e^{-\beta _1\tau _1}I_1\widetilde{{\mathcal {Q}}} \left( \dfrac{I}{I_1}\right) \\{} & {} +\dfrac{\delta }{k}e^{\beta _1\tau _1+\beta _2\tau _2}D_1 \widetilde{{\mathcal {Q}}}\left( \dfrac{D}{D_1}\right) \\{} & {} + \dfrac{\delta (\alpha +\delta )}{k\alpha }e^{\beta _1\tau _1 +\beta _2\tau _2+\beta _3\tau _3}V_1\widetilde{{\mathcal {Q}}}\left( \dfrac{V(x,t)}{V_1}\right) \\{} & {} + \dfrac{r\delta (\alpha +\delta )}{kb\alpha }e^{\beta _1\tau _1+\beta _2\tau _2+\beta _3\tau _3}W + \dfrac{p}{a}e^{\beta _1\tau _1}Z\\{} & {} + \displaystyle f(H_1,E_1,I_1,V_1)V_1\int _{t-\tau _1}^t\widetilde{{\mathcal {Q}}} \left( \dfrac{f(H(\theta ),V(\theta ))V(\theta )}{f(H_1,V_1)V_1}\right) d\theta \\{} & {} +\delta e^{\beta _1\tau _1}I_1\int _{t-\tau _2}^t\widetilde{{\mathcal {Q}}}\left( \dfrac{I(\theta )}{I_1}\right) d\theta \\{} & {} + \dfrac{\delta (\alpha +\delta )}{k}e^{\beta _1\tau _1+\beta _2\tau _2}D_1\displaystyle \int _{t-\tau _3}^t \widetilde{{\mathcal {Q}}}\left( \dfrac{D(\theta )}{D_1}\right) d\theta . \end{aligned}$$

Recall that $H_1,$ $E_1,$ $I_1,$ $D_1$ and $V_1$ are the first five components of the immune-free equilibrium secured by Theorem 4.1. It is obvious that the function ${\widetilde{L}}_1(t)$ is nonnegative definite in $[-\tau , 0]$ with respect to $P_1.$ Taking the time derivative of ${\widetilde{L}}_1(t)$ along the positive solution of system (2.1) and using the equilibrium conditions for $P_1,$ we obtain

$$\begin{aligned} \dfrac{d{\widetilde{L}}_1(t)}{dt}= & {} \displaystyle dH_1\left( 1-\dfrac{H}{H_1}\right) \left( 1-\dfrac{f(H_1,V_1)}{f(H,V_1)}\right) -f(H_1,V_1)V_1\left[ \widetilde{{\mathcal {Q}}} \left( \dfrac{f(H_{1},V_{1})}{f(H,V_1)}\right) \right. \\{} & {} \left. +\widetilde{{\mathcal {Q}}}\left( \dfrac{f(H,V_{1})}{f(H,V)}\right) +\widetilde{{\mathcal {Q}}}\left( \dfrac{D_1I_{\tau _2}}{DI_1}\right) +\widetilde{{\mathcal {Q}}}\left( \dfrac{D_{\tau _3}V_1}{VD_1}\right) +\widetilde{{\mathcal {Q}}}\left( \dfrac{f(H_{\tau _1},V_{\tau _1})V_{\tau _1}I_{1}}{f(H_1,V_1)V_1I}\right) \right] \\{} & {} +f(H_1,V_1)V_1\left( -1+\dfrac{f(H,V_{1})}{f(H,V)} -\dfrac{V}{V_1}+ \dfrac{f(H,V)V}{f(H,V_{1})V_1}\right) \\{} & {} +\dfrac{\delta rc(\alpha +\delta )}{kb\alpha }e^{\beta _1\tau _1+\beta _2\tau _2+\beta _3\tau _3} ({\mathcal {R}}_1-1)W+\dfrac{pq}{a}e^{\beta _1\tau _1}({\mathcal {R}}_2-1)Z. \end{aligned}$$

With the aid of $(B_4),$ we obtain

$$\begin{aligned} -1+\dfrac{f(H,V_{1})}{f(H,V)}-\dfrac{V}{V_1}+ \dfrac{f(H,V)V}{f(H,V_{1})V_1}= \left( 1-\dfrac{f(H,V)}{f(H,V_1)}\right) \left( \dfrac{f(H,V_1)}{f(H,V)}-\dfrac{V}{V_1}\right) \le 0. \end{aligned}$$

Since *f*(*H*, *V*) is a strictly monotonically nondecreasing function with respect to *H*,  by assumption $(B_2),$ then it is easy to show that assumption $(B_2)$ ultimately gives rise to the following inequality:

$$\begin{aligned} \left( 1-\dfrac{H}{H_1}\right) \left( 1-\dfrac{f(H_1,V_1)}{f(H,V_1)}\right) \le 0. \end{aligned}$$

Thus, if ${\mathcal {R}}_1\le 1$ and ${\mathcal {R}}_2\le 1,$ we get $\dfrac{d{\widetilde{L}}_1(t)}{dt}\le 0$ for all $H,I,D,V,W,Z>0$ with $\dfrac{d{\widetilde{L}}_1(t)}{dt}=0$ if and only if $H=H_1,$ $I=I_1,$ $D=D_1,$ $V=V_1,$ $W=0$ and $Z=0.$ Accordingly, ${\widetilde{L}}_1$ is a Lyapunov function. So, by LaSalle’s invariance principle (Hale and Verduyn Lunel 1993, Theorem 5.3.1), it follows that

5.13 $$\begin{aligned} \displaystyle \lim _{t\rightarrow \infty }(H,I,D,V,W,Z)=(H_1,I_1,D_1,V_1,0,0).\end{aligned}$$

Again, combining (5.13) with system (2.1), gives $\displaystyle \lim _{t\rightarrow \infty } E(t)=E_1$ as described in the proof of Theorem 5.3. Thus, every solution of the model approaches the unique immune-free equilibrium $P_1$ of system (2.1) when *t* tends to $\infty$ for ${\mathcal {R}}_0>1,$ ${\mathcal {R}}_1\le 1$ and ${\mathcal {R}}_2\le 1.$

Next, we discuss the stability property of the unique immune-free equilibrium $P_1$ when one of the following conditions ${\mathcal {R}}_1>1$ and ${\mathcal {R}}_2>1$ holds. From (5.1), by calculating, we get the characteristic equation of the linearization system of model (2.1) at the immune-free equilibrium $P_1$ as follows:

5.14 $$\begin{aligned} (\lambda +q-aI_1)(\lambda +c-bV_1)\text {g}_i(\lambda )=0,\end{aligned}$$

where

$$\begin{aligned} \text {g}_i(\lambda )=\left| \begin{array}{ccccc} a_{11}&{}0&{}0&{}0&{}a_{15}\\ a_{21}&{}a_{22}&{}0&{}0&{}a_{25}\\ a_{31}&{}0&{}a_{33}&{}0&{}a_{35}\\ 0&{}0&{}a_{43}&{}a_{44}&{}0\\ 0&{}0&{}0&{}a_{54}&{}a_{55} \end{array}\right| , \end{aligned}$$

with

$$a_{11}=\lambda +d+\frac{\partial f}{\partial H}V_1,\quad a_{15}=\frac{\partial f}{\partial V}V_1+f(H_1,V_1),\quad a_{21}=-(1-e^{-\lambda \tau _1-\beta _1\tau _1})\frac{\partial f}{\partial H}V_1,$$ $$\quad a_{22}=\lambda +\beta _1,$$

$$a_{25}=-(1-e^{-\lambda \tau _1-\beta _1\tau _1})\left( \frac{\partial f}{\partial V}V_1+f(H_1,V_1)\right) ,\quad a_{31}=-\frac{\partial f}{\partial H}V_1e^{-\lambda \tau _1-\beta _1\tau _1},\quad a_{33}=\lambda +\delta ,$$

$$a_{35}=-f(H_1,V_1)e^{-\lambda \tau _1-\beta _1\tau _1}\quad a_{43}=-ke^{-\lambda \tau _2-\beta _2\tau _2}, \quad a_{44}=\lambda +\alpha +\delta ,\quad a_{54}=-\alpha e^{-\lambda \tau _3-\beta _3\tau _3}, \quad a_{55}=\lambda +\mu .$$

By equation (5.14), it clearly appears that $\lambda _1=bV_1-c=c({\mathcal {R}}_1-1)$ and $\lambda _2=aI_1-q=q({\mathcal {R}}_2-1)$ are two reals roots of the characteristic equation (5.14). Thus, it follows that if ${\mathcal {R}}_1=\frac{bV_1}{c}>1$ then we get $\lambda _1>0$ and if ${\mathcal {R}}_2=\frac{aI_1}{q}>1$ then we get $\lambda _2>0.$ This implies that when one of the following conditions ${\mathcal {R}}_1>1$ and ${\mathcal {R}}_2>1$ holds then there exists a real positive root of the characteristic equation (5.14). Thus, if ${\mathcal {R}}_2>1$ or ${\mathcal {R}}_2>1,$ the immune-free equilibrium $P_1$ is unstable. This completes the proof. $\square$

Biologically speaking, the result of Theorem 5.4 means that HBV infection could persist if the adaptive immunity, represented by antibodies and CTLs, is not yet activated. Thus, this result exhibit a patient’s suffering state when his adaptive immune defense is not yet activated.

### Global asymptotic stability of the equilibrium $P_2$

In this section, again, by shaping a suitable Lyapunov function, we study the global asymptotic stability of the infection equilibrium with only antibody immune defense $P_2.$ The following result can be obtained.

#### Theorem 5.5

Let requirement $(B_4)$ and conditions ${\mathcal {R}}_0>1$ and ${\mathcal {R}}_1>1$ hold. Then if ${\mathcal {R}}_3\le 1,$ the infection equilibrium with only antibody immune defense $P_2$ of the delayed system (2.1) is globally asymptotically stable and becomes unstable whenever ${\mathcal {R}}_3>1.$

#### Proof

Let *H*(*t*), *E*(*t*), *I*(*t*), *D*(*t*), *V*(*t*), *W*(*t*), *Z*(*t*) be any arbitrary positive solution of problem (2.1). Define the following Lyapunov function

$$\begin{aligned} {\widetilde{L}}_2(t)= & {} \displaystyle H(t)-H_2-\int _{H_2}^{H(t)}\dfrac{f(H_2,V_2)}{f(s,V_2)}ds+e^{-\beta _1\tau _1}I_2\widetilde{{\mathcal {Q}}}\left( \dfrac{I(t)}{I_2}\right) \\{} & {} +\dfrac{\delta }{k}e^{\beta _1\tau _1+\beta _2\tau _2}D_2\widetilde{{\mathcal {Q}}}\left( \dfrac{D(t)}{D_2}\right) \\{} & {} + \dfrac{\delta (\alpha +\delta )}{k\alpha }e^{\beta _1\tau _1+\beta _2\tau _2+\beta _3\tau _3}V_2\widetilde{{\mathcal {Q}}}\left( \dfrac{V(t)}{V_2}\right) \\ {}{} & {} + \dfrac{r\delta (\alpha +\delta )}{kb\alpha }e^{\beta _1\tau _1+\beta _2\tau _2+\beta _3\tau _3}W_2\widetilde{{\mathcal {Q}}}\left( \dfrac{W(t)}{W_2}\right) \\{} & {} + \dfrac{p}{a}e^{\beta _1\tau _1}Z(t)+f(H_2,V_2)V_2\int _{t-\tau _1}^t\widetilde{{\mathcal {Q}}}\left( \dfrac{f(H(\theta ),V(\theta ))V(\theta )}{f(H_2,E_2,I_2,V_2)V_2}\right) d\theta \\{} & {} +\delta e^{\beta _1\tau _1}I_2\int _{t-\tau _2}^t\widetilde{{\mathcal {Q}}}\left( \dfrac{I(\theta )}{I_2}\right) d\theta + \dfrac{\delta (\alpha +\delta )}{k}e^{\beta _1\tau _1+\beta _2\tau _2}D_2\displaystyle \int _{t-\tau _3}^t\widetilde{{\mathcal {Q}}}\left( \dfrac{D(\theta )}{D_2}\right) d\theta . \end{aligned}$$

Recall that $H_2,$ $E_2,$ $I_2,$ $D_2,$ $V_2$ and $W_2$ are the first six components of the infection equilibrium with only antibody immune defense guaranteed by Theorem 4.1. By employing the equilibrium conditions for $P_2$, after lengthy calculations, the derivative of the above Lyapunov function computed along the solutions of system (2.1) is given below:

$$\begin{aligned} \dfrac{d{\widetilde{L}}_2(t)}{dt}\le & {} \displaystyle dH_2\left( 1-\dfrac{H}{H_2}\right) \left( 1-\dfrac{f(H_2,V_2)}{f(H,V_2)}\right) \\{} & {} -f(H_2,V_2)V_2\left[ \widetilde{{\mathcal {Q}}}\left( \dfrac{f(H_{2},V_{2})}{f(H,V_2)}\right) +\widetilde{{\mathcal {Q}}}\left( \dfrac{f(H,V_{2})}{f(H,V)}\right) \right. \\{} & {} +\left. \widetilde{{\mathcal {Q}}}\left( \dfrac{D_2I_{\tau _2}}{DI_2}\right) +\widetilde{{\mathcal {Q}}}\left( \dfrac{D_{\tau _3}V_2}{VD_2}\right) +\widetilde{{\mathcal {Q}}}\left( \dfrac{f(H_{\tau _1},V_{\tau _1})V_{\tau _1}I_{2}}{f(H_2,V_2)V_2I}\right) \right] +\dfrac{pq}{a}e^{\beta _1\tau _1}({\mathcal {R}}_3-1)Z \\{} & {} +f(H_2,V_2)V_2\left( 1-\dfrac{f(H,V)}{f(H,V_2)}\right) \left( \dfrac{f(H,V_2)}{f(H,V)}-\dfrac{V}{V_2}\right) . \end{aligned}$$

Since *f*(*H*, *E*, *I*, *V*) is a strictly monotonically nondecreasing function with respect to *H*,  by assumption $(B_2),$ then it is easy to show that assumption $(B_2)$ ultimately gives rise to the following inequality:

$$\begin{aligned} \left( 1-\dfrac{H}{H_2}\right) \left( 1-\dfrac{f(H_2,V_2)}{f(H,V_2)}\right) \le 0. \end{aligned}$$

Thus, using assumption $(B_4),$ it follows that if ${\mathcal {R}}_3\le 1,$ we have $\frac{d{\widetilde{L}}_2(t)}{dt}\le 0$ for all *H*,  *I*,  *D*,  *V*,  *W*,  $Z>0,$ with $\frac{d{\widetilde{L}}_2(t)}{dt}=0$ if and only if $H=H_2,$ $I=I_2,$ $D=D_2,$ $V=V_2,$ $W=W_2$ and $Z_2=0.$ Combining this with the delayed model (2.1), we have $E=E_2.$ This indicates that the largest compact invariant subset in $\left\{ (H,E,I, D, V, W, Z)\in {\mathbb {R}}_+^7: \frac{d{\widetilde{L}}_2}{dt}=0\right\}$ is the singleton set $\{P_2\}.$ Hence, by LaSalle’s invariance principle (Hale and Verduyn Lunel 1993, Theorem 5.3.1), it follows that the unique infection equilibrium with only antibody immune defense $P_2$ is globally asymptotically stable when ${\mathcal {R}}_0>1,$ ${\mathcal {R}}_1>1$ and ${\mathcal {R}}_3\le 1.$

To end the proof, we investigate the stability property of the infection equilibrium with only antibody immune defense $P_2$ when the following condition ${\mathcal {R}}_3>1$ holds. Again from (5.1), by simple calculation, we get the characteristic equation of the linearization system of model (2.1) at the equilibrium steady state $P_2$ as follows:

5.15 $$\begin{aligned} (\lambda +q-aI_2)\text {g}^\natural _i(\lambda )=0,\end{aligned}$$

where

$$\begin{aligned} \text {g}^\natural _i(\lambda )=\left| \begin{array}{cccccc} a_{11}&{}0&{}0&{}0&{}a_{15}&{}0\\ a_{21}&{}a_{22}&{}0&{}0&{}a_{25}&{}0\\ a_{31}&{}0&{}a_{33}&{}0&{}a_{35}&{}0\\ 0&{}0&{}a_{43}&{}a_{44}&{}0&{}0\\ 0&{}0&{}0&{}a_{54}&{}a_{55}&{}a_{56}\\ 0&{}0&{}0&{}0&{}a_{65}&{} a_{66} \end{array}\right| , \end{aligned}$$

with

$$a_{11}=\lambda +d+\frac{\partial f}{\partial H}V_2,\quad a_{15}=\frac{\partial f}{\partial V}V_2+f(H_2,V_2),\quad a_{21}=-(1-e^{-\lambda \tau _1-\beta _1+\tau _1})\frac{\partial f}{\partial H}V_2,\quad a_{22}=\lambda +\beta _1$$

$$a_{25}=-(1-e^{-\lambda \tau _1-\beta _1\tau _1})\left( \frac{\partial f}{\partial H}V_2+f(H_2,V_2)\right) ,\quad a_{31}=-\frac{\partial f}{\partial H}V_2e^{-\lambda \tau _1-(\beta _1+\sigma )\tau _1},\quad a_{33}=\lambda +\delta ,$$

$$ a_{35}=-f(H_2,V_2)e^{-\lambda \tau _1-\beta _1\tau _1},\quad a_{43}=-ke^{-\lambda \tau _2-\beta _2\tau _2},\quad a_{44}=\lambda +\alpha +\delta ,\quad a_{54}=-\alpha e^{-\lambda \tau _3-\beta _3\tau _3}, $$

$$a_{55}=\lambda +\mu +rW_2,\quad a_{56}=rV_2,\quad a_{65}=-bW_2,\quad a_{66}=\lambda +c-bV_2.$$

From equation (5.15), it is seen that $\lambda _1=aI_2-q=q({\mathcal {R}}_3-1)$ denotes a real root of the characteristic equation (5.15). Therefore, it follows that if ${\mathcal {R}}_3=\frac{aI_2}{q}>1$ then we get $\lambda _1>0.$ This indicate that when condition ${\mathcal {R}}_3>1$ holds, there exists a real positive root of the characteristic equation (5.15). Hence, if ${\mathcal {R}}_3>1$ the infection equilibrium with only antibody immune defense $P_2$ is unstable. This achieves the proof. $\square$

Theorem 5.5 communicates that the infection could persist due to the absence of one component of adaptive immunity. In other words, the body with only antibody immune response activated cannot prevent the progression of the viral infection.

### Global asymptotic stability of the equilibrium $P_3$

In this section, again, by shaping a suitable Lyapunov function, we study the global asymptotic stability of the infection equilibrium with only CTL immune response $P_3.$ The following result can be obtained.

#### Theorem 5.6

Let assumption $(B_4)$ and conditions ${\mathcal {R}}_0>1$ and ${\mathcal {R}}_2>1$ be valid. Then if ${\mathcal {R}}_4\le 1,$ the infection equilibrium with only CTL immune response $P_3$ of the delayed diffusive problem (2.1) is globally asymptotically stable and becomes unstable whenever ${\mathcal {R}}_4>1.$

#### Proof

Let *H*(*t*), *E*(*t*), *I*(*t*), *D*(*t*), *V*(*t*), *W*(*t*), *Z*(*t*) be any arbitrary positive solution of problem (2.1). Define the following Lyapunov function

$$\begin{aligned} {\widetilde{L}}_3(t)= & {} \displaystyle H(x,t)-H_3-\int _{H_3}^{H(x,t)} \dfrac{f(H_3,V_3)}{f(s,V_3)}ds+e^{\beta _1\tau _1}I_3\widetilde{{\mathcal {Q}}}\left( \dfrac{I(t)}{I_3}\right) \\{} & {} + \dfrac{\delta +pZ_3}{k}e^{\beta _1\tau _1+\beta _2\tau _2}D_3\widetilde{{\mathcal {Q}}}\left( \dfrac{D(t)}{D_3}\right) \\{} & {} + \dfrac{(\delta +pZ_3)(\alpha +\delta )}{k\alpha }e^{\beta _1\tau _1+\beta _2\tau _2+\beta _3\tau _3}V_3\widetilde{{\mathcal {Q}}}\left( \dfrac{V(t)}{V_3}\right) \\ {}{} & {} + \dfrac{r(\delta +pZ_3)(\alpha +\delta )}{kb\alpha }e^{\beta _1\tau _1+\beta _2\tau _2+\beta _3\tau _3}W(t) \\{} & {} + \dfrac{p}{a}e^{\beta _1\tau _1}Z_3\widetilde{{\mathcal {Q}}}\left( \dfrac{Z(t)}{Z_3}\right) \\{} & {} + \displaystyle f(H_3,V_3)V_3\int _{t-\tau _1}^t\widetilde{{\mathcal {Q}}}\left( \dfrac{f(H(\theta ),V(\theta ))V(\theta )}{f(H_3,V_3)V_3}\right) d\theta \\{} & {} +(\delta +pZ_3)e^{\beta _1\tau _1}I_3\int _{t-\tau _2}^t\widetilde{{\mathcal {Q}}}\left( \dfrac{I(\theta )}{I_3}\right) d\theta \\{} & {} + \dfrac{(\delta +pZ_3)(\alpha +\delta )}{k}e^{\beta _1\tau _1+\beta _2\tau _2}D_3\displaystyle \int _{t-\tau _3}^t\widetilde{{\mathcal {Q}}}\left( \dfrac{D(\theta )}{D_3}\right) d\theta . \end{aligned}$$

Recall that $H_3,$ $E_3,$ $I_3,$ $D_3,$ $V_3$ and $Z_3$ are the first five and last components of the infection equilibrium with only CTL immune response $P_3$ guaranteed by Theorem 4.1. By employing the equilibrium conditions for $P_3$, after lengthy calculations, the derivative of the above Lyapunov function computed along the solutions of system (2.1) is given below:

$$\begin{aligned} \dfrac{d{\widetilde{L}}_3(t)}{dt}\le & {} \displaystyle dH_3\left( 1-\dfrac{H}{H_3}\right) \left( 1-\dfrac{f(H_3,V_3)}{f(H,V_3)}\right) -f(H_3,V_3)V_3\left[ \widetilde{{\mathcal {Q}}} \left( \dfrac{f(H_3,V_3)}{f(H,V_3)}\right) \right. \\{} & {} +\widetilde{{\mathcal {Q}}}\left( \dfrac{f(H,V_3)}{f(H,V)}\right) \\{} & {} \left. +\widetilde{{\mathcal {Q}}}\left( \dfrac{D_3I_{\tau _2}}{DI_3}\right) +\widetilde{{\mathcal {Q}}} \left( \dfrac{D_{\tau _3}V_3}{VD_3}\right) +\widetilde{{\mathcal {Q}}}\left( \dfrac{f(H_{\tau _1}, V_{\tau _1})V_{\tau _1}I_{3}}{f(H3,V_3)V_3I}\right) \right] +f(H_3,V_3)V_3\\{} & {} \times \left( 1-\dfrac{f(H,V)}{f(H,V_3)}\right) \left( \dfrac{f(H,V_3)}{f(H,V)}-\dfrac{V}{V_3}\right) \\{} & {} +\dfrac{(\delta +pZ_3) rc(\alpha +\delta )}{kb\alpha }e^{\beta _1\tau _1+\beta _2\tau _2+\beta _3\tau _3}({\mathcal {R}}_4-1)W. \end{aligned}$$

Since *f*(*H*, *E*, *I*, *V*) is a strictly monotonically nondecreasing function with respect to *H*,  by assumption $(B_2),$ then it is easy to show that assumption $(B_2)$ ultimately gives rise to the following inequality:

$$\begin{aligned} \left( 1-\dfrac{H}{H_3}\right) \left( 1-\dfrac{f(H_3,V_3)}{f(H,V_3)}\right) \le 0. \end{aligned}$$

Thus, using assumption $(B_4),$ it follows that if ${\mathcal {R}}_4\le 1,$ we get $\dfrac{d{\widetilde{L}}_3(t)}{dt}\le 0$ for all $H,E,I,D,V,W,Z>0,$ with $\dfrac{d{\widetilde{L}}_3(t)}{dt}=0$ if and only if $H=H_3,$ $I=I_3,$ $D=D_3,$ $V=V_3,$ $W=0$ and $Z=Z_3.$ Combining this with the system (2.1), we have $E=E_3.$ This indicates that the largest compact invariant subset in $\left\{ (H,I, D, V, W, Z)\in {\mathbb {R}}_+^7: \frac{d{\widetilde{L}}_3}{dt}=0\right\}$ is the singleton set $\{P_3\}.$ Therefore, by LaSalle’s invariance principle (Hale and Verduyn Lunel 1993, Theorem 5.3.1), it follows that the infection equilibrium with only CTL response $P_3$ is globally asymptotically stable when ${\mathcal {R}}_0>1,$ ${\mathcal {R}}_2>1$ and ${\mathcal {R}}_4\le 1.$

We now study the stability property of the infection equilibrium with only CTL response $P_3$ when the following condition ${\mathcal {R}}_4>1$ holds. Again from (5.1), by simple calculation, we get the characteristic equation of the linearization system of model (2.1) at the equilibrium steady state $P_3$ as follows:

5.16 $$\begin{aligned} (\lambda +c-bV_3)\text {g}^{\natural \natural }_i(\lambda )=0,\end{aligned}$$

where

$$\begin{aligned} \text {g}^{\natural \natural }_i(\lambda )=\left| \begin{array}{cccccc} a_{11}&{}0&{}0&{}0&{}a_{15}&{}0\\ a_{21}&{}a_{22}&{}0&{}0&{}a_{25}&{}0\\ a_{31}&{}0&{}a_{33}&{}0&{}a_{35}&{}a_{36}\\ 0&{}0&{}a_{43}&{}a_{44}&{}0&{}0\\ 0&{}0&{}0&{}a_{54}&{}a_{55}&{}0\\ 0&{}0&{}a_{63}&{}0&{}0&{} a_{66} \end{array}\right| , \end{aligned}$$

with

$$a_{11}=\lambda +d+\frac{\partial f}{\partial H}V_3,\quad a_{15}=\frac{\partial f}{\partial V}V_3+f(H_3,V_3),\quad a_{21}=-(1-e^{-\lambda \tau _1-\beta _1\tau _1})\frac{\partial f}{\partial H}V_3,\quad a_{22}=\lambda +\beta _1,$$

$$a_{25}=-(1-e^{-\lambda \tau _1-(\beta _1+\sigma )\tau _1})\left( \frac{\partial f}{\partial V}V_3+f(H_3,V_3)\right) ,\quad a_{31}=-\frac{\partial f}{\partial H}V_3e^{-\lambda \tau _1-(\beta _1+\sigma )\tau _1},\quad a_{33}=\lambda +\delta +pZ_3,$$

$$a_{35}=-f(H_3,V_3)e^{-\lambda \tau _1-\beta _1\tau _1},\quad a_{36}=pI_3,\quad a_{43}=-ke^{-\lambda \tau _2-\beta _2\tau _2},\quad \quad a_{44}=\lambda +\alpha +\delta ,$$

$$a_{54}=-\alpha e^{-\lambda \tau _3-\beta _3\tau _3}, \quad a_{55}=\lambda +\mu ,\quad a_{63}=-aZ_3,\quad a_{66}=\lambda +q-aI_3.$$

From equation (5.16), it is seen that $\lambda _1=bV_3-c=c({\mathcal {R}}_4-1)$ denotes a real root of the characteristic equation (5.16). Therefore, it follows that if ${\mathcal {R}}_4=\frac{bV_3}{c}>1$ then we get $\lambda _1>0.$ This indicate that when condition ${\mathcal {R}}_4>1$ holds, there exists a real positive root of the characteristic equation (5.16). Hence, if ${\mathcal {R}}_4>1$ the infection equilibrium with only CTL response $P_3$ is unstable. This achieves the proof. $\square$

Theorem 5.6 communicates that the infection could persist due to the absence of antibody immune response. In other words, the body with only CTL immune defense activated cannot prevent the progression of the viral infection. This conclusion and the one exhibited by Theorem 5.5, imply that an infected person may suffer from HBV infection symptoms if his total immune defense is not activated.

### Global asymptotic stability of the equilibrium $P_4$

In this section, again, by constructing a crafty Lyapunov function, we investigate the global asymptotic stability of the infection equilibrium with CTL and antibody immune defense $P_4.$ The following result can be obtained.

#### Theorem 5.7

Let assumption $(B_4)$ be valid. If ${\mathcal {R}}_0>1,$ ${\mathcal {R}}_1>1,$ ${\mathcal {R}}_2>1,$ ${\mathcal {R}}_3>1$ and ${\mathcal {R}}_4>1,$ then the infection equilibrium with CTL and antibody immune defense $P_4$ of the delayed diffusive problem (2.1) is globally asymptotically stable.

#### Proof

Let *H*(*t*), *E*(*t*), *I*(*t*), *D*(*t*), *V*(*t*), *W*(*t*), *Z*(*t*) be any arbitrary positive solution of problem (2.1). Define the following Lyapunov function

$$\begin{aligned} {\widetilde{L}}_4(t)= & {} \displaystyle H(t)-H_4-\int _{H_4}^{H(t)}\dfrac{f(H_4,V_4)}{f(s,V_4)}ds+e^{\beta _1\tau _1}I_4\widetilde{{\mathcal {Q}}}\left( \dfrac{I(t)}{I_4}\right) \\{} & {} + \dfrac{\delta +pZ_4}{k}e^{\beta _1\tau _1+\beta _2\tau _2}D_4\widetilde{{\mathcal {Q}}}\left( \dfrac{D(t)}{D_4}\right) \\{} & {} + \dfrac{(\delta +pZ_4)(\alpha +\delta )}{k\alpha }e^{\beta _1\tau _1+\beta _2\tau _2+\beta _3\tau _3}V_4\widetilde{{\mathcal {Q}}}\left( \dfrac{V(t)}{V_4}\right) \\{} & {} + \dfrac{r(\delta +pZ_4)(\alpha +\delta )}{kb\alpha }e^{\beta _1\tau _1+\beta _2\tau _2+\beta _3\tau _3}W_4\widetilde{{\mathcal {Q}}}\left( \dfrac{W(t)}{W_4}\right) \\{} & {} + \dfrac{p}{a}e^{\beta _1\tau _1}Z_4\widetilde{{\mathcal {Q}}}\left( \dfrac{Z(t)}{Z_4}\right) + f(H_4,V_4)V_4\int _{t-\tau _1}^t\widetilde{{\mathcal {Q}}}\left( \dfrac{f(H(\theta ),V(\theta ))V(\theta )}{f(H_4,V_4)V_4}\right) d\theta \\{} & {} +(\delta +pZ_4)e^{\beta _1\tau _1}I_4\int _{t-\tau _2}^t\widetilde{{\mathcal {Q}}}\left( \dfrac{I(\theta )}{I_4}\right) d\theta \\{} & {} + \dfrac{(\delta +pZ_4)(\alpha +\delta )}{k}e^{\beta _1\tau _1+\beta _2\tau _2}D_4\displaystyle \int _{t-\tau _3}^t\widetilde{{\mathcal {Q}}}\left( \dfrac{D(\theta )}{D_4}\right) d\theta . \end{aligned}$$

Recall that $H_4,$ $E_4,$ $I_4,$ $D_4,$ $V_4,$ $W_4$ and $Z_4$ denote the components of the infection equilibrium with both CTL and antibody immune defense $P_4$ guaranteed by Theorem 4.1. By employing the equilibrium conditions for $P_4$, after lengthy calculations, the derivative of the above Lyapunov function computed along the solutions of system (2.1) is given below:

$$\begin{aligned} \dfrac{d{\widetilde{L}}_4(t)}{dt}\le & {} \displaystyle dH_4\left( 1-\dfrac{H}{H_4}\right) \left( 1-\dfrac{f(H_4,V_4)}{f(H,V_4)}\right) \\{} & {} -f(H_4,V_4)V_4 \left[ \widetilde{{\mathcal {Q}}}\left( \dfrac{f(H_4,V_4)}{f(H,V_4)}\right) +\widetilde{{\mathcal {Q}}}\left( \dfrac{f(H,V_4)}{f(H,V)}\right) \right. \\{} & {} +\widetilde{{\mathcal {Q}}}\left( \dfrac{D_4I_{\tau _2}}{DI_4}\right) \\{} & {} +\widetilde{{\mathcal {Q}}}\left( \dfrac{D_{\tau _3}V_4}{VD_4}\right) \left. +\widetilde{{\mathcal {Q}}} \left( \dfrac{f(H_{\tau _1},V_{\tau _1})V_{\tau _1}I_{4}}{f(H_4,V_4)V_4I}\right) \right] \\{} & {} +f(H_4,V_4)V_4\left( 1-\dfrac{f(H,V)}{f(H,V_4)}\right) \left( \dfrac{f(H,V_4)}{f(H,V)}-\dfrac{V}{V_4} \right) . \end{aligned}$$

Since *f*(*H*, *E*, *I*, *V*) is a strictly monotonically nondecreasing function with respect to *H*,  by assumption $(B_2),$ then it is easy to show that assumption $(B_2)$ ultimately gives rise to the following inequality:

$$\begin{aligned} \left( 1-\dfrac{H}{H_4}\right) \left( 1-\dfrac{f(H_4,V_4)}{f(H,V_4)}\right) \le 0. \end{aligned}$$

Therefore, using assumption $(B_4),$ we get $\frac{d{\widetilde{L}}_4(t)}{dt}\le 0$ for all $H,E,I,D,V,W,Z>0$ with $\frac{d{\widetilde{L}}_4(t)}{dt}=0$ if and only if $H=H_4,$ $I=I_4,$ $D=D_4,$ $V=V_4,$ $W=W_4$ and $Z=Z_4.$ Combining this with the system (2.1), we have $E=E_4.$ This indicates that the largest compact invariant subset in $\left\{ (H,I, D, V, W, Z)\in {\mathbb {R}}_+^7: \frac{d{\widetilde{L}}_4}{dt}=0\right\}$ is the singleton set $\{P_4\}.$ Hence, by LaSalle’s invariance principle (Hale and Verduyn Lunel 1993, Theorem 5.3.1), it follows that the infection equilibrium with CTL and antibody immune defense $P_4$ is globally asymptotically stable when ${\mathcal {R}}_0>1,$ ${\mathcal {R}}_1>1,$ ${\mathcal {R}}_2>1,$ ${\mathcal {R}}_3>1$ and ${\mathcal {R}}_4>1.$ This completes the proof.$\square$

Theorem 5.7 communicates that HBV infection could persist from a body even in the presence of adaptive immunity which is represented by antibodies and CTLs.

## Application and numerical simulations

This section is devoted to the application of theoretical results obtained in the previous sections by performing some numerical simulations. For this purpose, we consider the following particular Crowley-Martin functional response $f(H,V)=\frac{\beta _0H}{1+a_0H+b_0V+a_0b_0HV}$ (Kang et al. 2017). In this case, the generalized model (2.1) turns into the following particular delayed model

6.1 $$\begin{aligned} {\left\{ \begin{array}{ll} \frac{dH}{dt}=s_0-dH(t)-\dfrac{\beta _0H(t)V(t)}{1+a_0H(t)+b_0V(t)+a_0b_0H(t)V(t)},\\ \frac{dE}{dt}=\dfrac{\beta _0H(t)V(t)}{1+a_0H(t)+b_0V(t)+a_0b_0H(t)V(t)}\\ \qquad - \dfrac{\beta _0e^{-\beta _1\tau _1}H(t-\tau _1)V(t-\tau _1)}{1+a_0H(t-\tau _1)+b_0V(t-\tau _1)+a_0b_0H(t-\tau _1)V(t-\tau _1)}-\beta _1E(t),\\ \frac{dI}{dt}= \dfrac{\beta _0e^{-\beta _1\tau _1}H(t-\tau _1)V(t-\tau _1)}{1+a_0H(t-\tau _1)+b_0V(t-\tau _1)+a_0b_0H(t-\tau _1)V(t-\tau _1)}-\delta I(t)-pI(t)Z(t),\\ \frac{dD}{dt}=k e^{-\beta _2\tau _2}I(t-\tau _2)-(\alpha +\delta )D(t),\\ \frac{dV}{dt}=\alpha e^{-\beta _3\tau _3}D(t-\tau _3)-\mu V(t)-rV(x,t)W(t),\\ \frac{dW}{dt}=bV(t)W(t)-cW(t),\\ \frac{dZ}{dt}=aI(t)Z(t)-qZ(t), \end{array}\right. } \end{aligned}$$

subjected to the nonnegative initial conditions (2.2). Clearly, it can be seen that for the specific form of functional response choosen, the hypotheses $(B_1)$-$(B_3)$ are satisfied. Moreover, it is straightforward to check that assumption $(B_4)$ is satisfied. We note that the choice of Crowley-Martin functional response here is that it: generalizes many common types existing in the literature, some of which are given in section 2; describes the infection of healthy target cells by the free virions; and considers the inhibitory or physiological effects of virus. Moreover, the nonnegative constants $a_0,$ $b_0$ and $c_0=a_0b_0$ are saturation factors measuring the inhibitory or physiological effect.

The biological description of the parameters as well as their values and units are summed up in Table 1.

The infection-free equilibrium of the particular system (6.1) is given by $P_0=({s_0}/{d},0,0,0,0,0,0)$ and the basic reproduction number ${\mathcal {R}}_0$ and other reproduction numbers ${\mathcal {R}}_1,$ ${\mathcal {R}}_2,$ ${\mathcal {R}}_3$ and ${\mathcal {R}}_4$ are given by

**Table 1:** Biological significance, estimated value and unit of the parameters of the model (6.1)

Parameters	Biological significance	Baseline value	Unit	Source
$s_0$	Production rate of healthy hepatocytes	$2.6\times 10^7$	cells $\hbox {mm}^{-3}$ $\hbox {day}^{-1}$	(Guo et al. 2018; Manna 2018; Manna and Hattaf 2019)
*d*	Death rate of healthy hepatocytes	0.01	$\hbox {day}^{-1}$	(Elaiw 2015; Elaiw and Agha 2019; Guo et al. 2018; Manna 2018; Manna and Hattaf 2019)
$\beta _0$	Transmission rate of infection	varied	$\hbox {virus}^{-1}$ $\hbox {mm}^3$ $\hbox {day}^{-1}$	Assumed
$\beta _1$	Death rate of exposed cells	0.01	$\hbox {day}^{-1}$	Assumed
$\delta$	Death rate of infected hepatocytes and Capsids	0.053	$\hbox {day}^{-1}$	(Guo et al. 2018; Manna 2018; Manna and Hattaf 2019)
*p*	CTL effectiveness	0.95	$\hbox {mm}^{3}$ $\hbox {cell}^{-1}$ $\hbox {day}^{-1}$	(Manna 2018; Manna and Hattaf 2019)
*k*	Capsids production rate	150	capsids $\hbox {cells}^{-1}$ $\hbox {day}^{-1}$	(Guo et al. 2018; Manna 2018; Manna and Hattaf 2019)
$\alpha$	Virion production rate	0.87	virion $\hbox {cells}^{-1}$ $\hbox {day}^{-1}$	(Manna 2018; Manna and Hattaf 2019)
$\mu$	Decay rate of HBV free virions	3.8	$\hbox {day}^{-1}$	(Guo et al. 2018; Manna 2018; Manna and Hattaf 2019)
*r*	Neutralizing rate of antibody	0.3	ml $\hbox {cells}^{-1}$ $\hbox {day}^{-1}$	(Manna and Hattaf 2019)
*b*	Antibody activation rate	varied	$\hbox {mm}^3$ $\hbox {virus}^{-1}$ $\hbox {day}^{-1}$	Assumed
*c*	Antibody death rate	0.05	$\hbox {day}^{-1}$	(Manna and Hattaf 2019)
*a*	CTL activation rate	0.2	$\hbox {mm}^3$ $\hbox {cell}^{-1}$ $\hbox {day}^{-1}$	(Manna and Hattaf 2019)
*q*	CTL death rate	varied	$\hbox {day}^{-1}$	Assumed
$a_0$	Crowley-Martin coefficient	varied	$\hbox {mm}^3$ $\hbox {cells}^{-1}$	Assumed
$b_0$	Crowley-Martin coefficient	1	$\hbox {mm}^3$ $\hbox {virus}^{-1}$	Assumed
$\beta _2$	Death rate for new capsids during $[t-\tau _2, t]$	0.01	$\hbox {day}^{-1}$	(Manna and Hattaf 2019)
$\beta _3$	Death rate for new virion during $[t-\tau _3, t]$	0.05	$\hbox {day}^{-1}$	(Manna and Hattaf 2019)

$$\begin{aligned}{} & {} {\mathcal {R}}_0= \dfrac{k\alpha s_0 \beta _0}{\delta \mu (d+a_0s_0)(\alpha +\delta )}e^{-\beta _1\tau _1-\beta _2\tau _2 -\beta _3\tau _3},\\{} & {} {\mathcal {R}}_1=\dfrac{kb\alpha \beta _0H_1V_1e^{-\beta _1\tau _1-\beta _2\tau _2 -\beta _3\tau _3}}{\delta \mu c(\alpha +\delta )(1+a_0H_1+b_0V_1+a_0b_0H_1V_1)},\\{} & {} {\mathcal {R}}_2=\dfrac{a\beta _0H_1V_1e^{-\beta _1\tau _1}}{\delta q(1+a_0H_1+b_0V_1+a_0b_0H_1V_1)},\\{} & {} {\mathcal {R}}_3=\dfrac{a\beta _0H_2V_2e^{-\beta _1\tau _1}}{\delta q(1+a_0H_2+b_0V_2+a_0b_0H_2V_2)},\quad {\mathcal {R}}_4=\dfrac{k\alpha bq}{a\mu c(\alpha +\delta )}e^{-\beta _2\tau _2-\beta _3\tau _3},\end{aligned}$$

respectively, where

$$\begin{aligned}{} & {} H_1=\dfrac{a_0b_0s_0-\beta _0-b_0d+a_0\zeta _1}{2da_0b_0} + \dfrac{\sqrt{(a_0b_0s_0-\beta _0-b_0d+a_0\zeta _1)^2+4da_0b_0(\zeta _1+b_0s_0)}}{2da_0b_0},\\{} & {} H_2=\dfrac{(s_0a_0-d)(1+b_0V_2)-\beta _0V_2}{2da_0(1+b_0V_2)} +\dfrac{\sqrt{[(s_0a_0-d)(1+b_0V_2)-\beta _0V_2]^2+4ds_0a_0(1+b_0V_2)^2}}{2da_0(1+b_0V_2)}, \\{} & {} V_1=\dfrac{\beta _0H_1-\zeta _1(1+a_0H_1)}{\zeta _1b_0(1+a_0H_1)}, \quad \zeta _1=\dfrac{\delta \mu (\alpha +\delta )}{k\alpha }e^{\beta _1\tau _1+\beta _2\tau _2+\beta _3\tau _3}, \quad V_2=\dfrac{c}{b}. \end{aligned}$$

Note that from the biological point of view, the basic reproduction number

${\mathcal {R}}_0=\frac{k\alpha e^{-\beta _1\tau _1-\beta _2\tau _2 -\beta _3\tau _3}}{\delta \mu (\alpha +\delta )} \frac{\beta _0s_0}{(d+a_0s_0)}$ of model (6.1) subjected to the nonnegative initial conditions (2.2), is not proportional to $\frac{s_0}{d}$ which represents the number of all cells in the liver of a patient. Thus the artifact stated in Gourley et al. (2008) when the mass action incidence function is used, is avoided. Therefore, the Crowley-Martin functional response considered makes our system more realistic for the dynamics of HBV infection.

For all numerical simulations, we take different initial conditions for each scenario and varied the values of the parameters $\beta _0,$ *b*,  $\tau _1,$ $a_0$ and *q*,  as they get the most important effects on the global stability of the steady state constants. From experimental data and literatures (Elaiw 2015; Elaiw and Agha 2019; Guo et al. 2018; Manna 2018; Manna and Hattaf 2019), we set the values of all other parameters in Table 1. For each equilibrium point, we take the values of $\tau _2$ and $\tau _3$ as in Manna and Hattaf (2019).

**Fig. 2:**
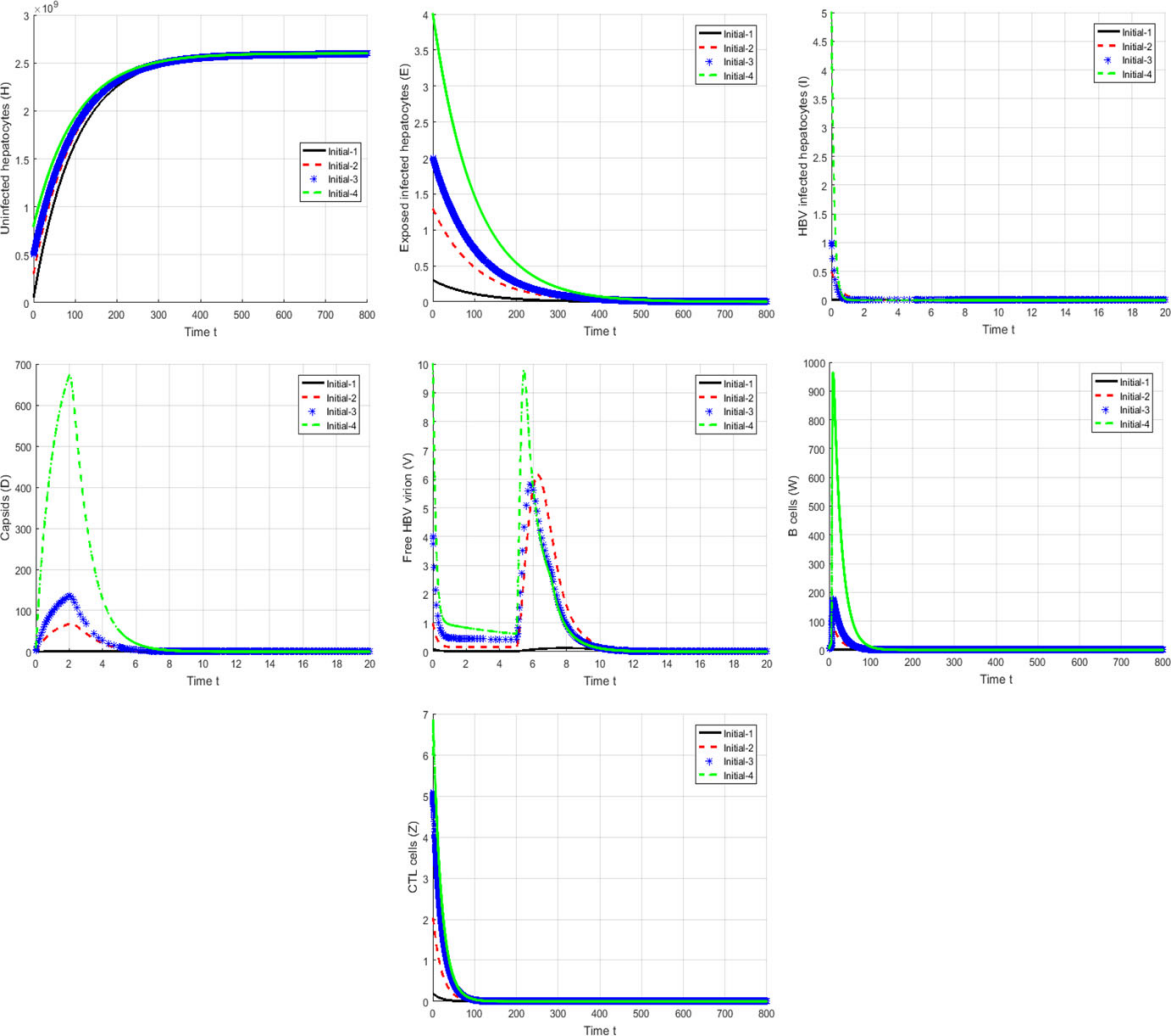
Simulations of model (4.1) using various initial conditions with $\beta _0=10^{-3},$ $b=0.3,$ $\tau _1=1,$ $\tau _2=2,$ $\tau _3=5,$ $a_0=1$ and $q=0.05$ so that ${\mathcal {R}}_0=0.5306<1$

Firstly, when $\beta _0=10^{-3},$ $b=0.3,$ $\tau _1=1,$ $\tau _2=2,$ $\tau _3=5,$ $a_0=1$ and $q=0.05,$ we obtain ${\mathcal {R}}_0=0.5306 <1,$ which means that the solution trajectories asymptotically approach towards the infection-free steady state $P_0=(2.6\times 10^9,0,0,0,0,0,0),$ as can be observed in Fig. 2. Here, the parameter $\beta _0$ was obtained from Elaiw and Agha (2019), *b* and *q* are from Manna and Hattaf (2019). We chose four sets of initial conditions as

Initial-1: $$(H(\theta ),E(\theta ),I(\theta ),D(\theta ),V(\theta ),W(\theta ),Z(\theta ))=(5\times 10^7,0.3,0.005,0.1,0.1,0.2,0.2),$$

Initial-2: $$(H(\theta ),E(\theta ),I(\theta ),D(\theta ),V(\theta ),W(\theta ),Z(\theta ))=(5\times 10^8,1.3,0.05,1,1,2,2),$$

Initial-3: $$(H(\theta ),E(\theta ),I(\theta ),D(\theta ),V(\theta ),W(\theta ),Z(\theta ))=(5\times 10^8,2,1,4,4,5,5),$$

Initial-4: $$(H(\theta ),E(\theta ),I(\theta ),D(\theta ),V(\theta ),W(\theta ),Z(\theta ))=(5\times 10^8,4,5,10,10,6,6),$$

for $\theta \in [-5,0].$ Here, we observe that healthy hepatocytes increase and attain their maximum level $2.6\times 10^9.$ Meanwhile, the other viral infection components converge toward zero. This confirms the result stated in Theorem 5.3 and it then follows that the infection is clear out.

**Fig. 3:**
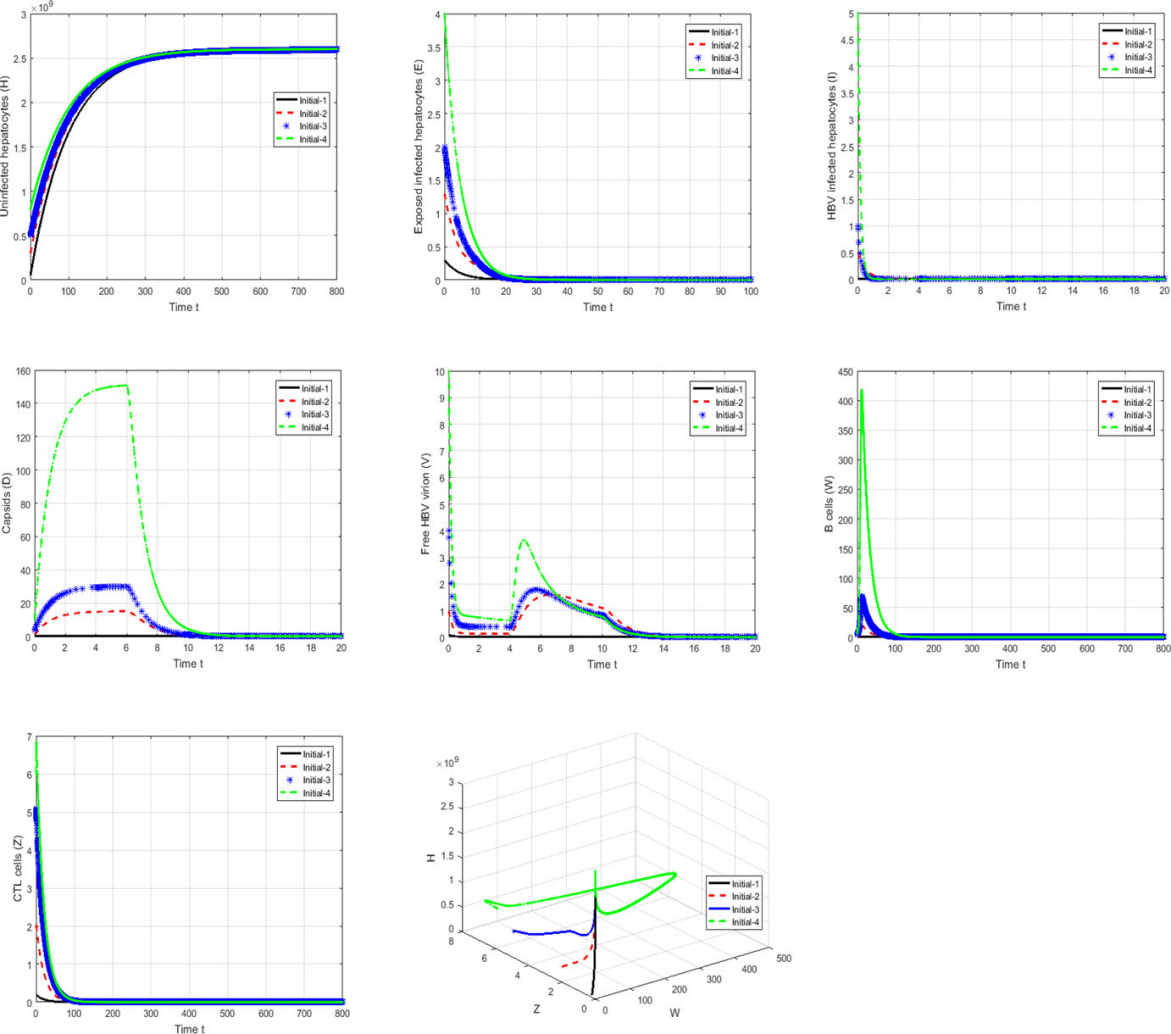
Simulations of model (4.1) using various initial conditions with $\beta _0=10^{-3},$ $\beta _1=0.2,$ $\beta _2=0.28,$ $\beta _3=0.1,$ $b=0.3,$ $\tau _1=5.8,$ $\tau _2=6,$ $\tau _3=4,$ $a_0=1$ and $q=0.05$ so that ${\mathcal {R}}_0=0.0275<1$. All other parameters as in Table 1. The infection-free equilibrium $P_0$ is globally asymptotically stable

Now, fixing the other parameters, and considering the values of the parameters $\beta _1=0.2,$ $\beta _2=0.28,$ $\beta _3=0.1,$ $\tau _1=5.8,$ $\tau _2=6$ and $\tau _3=4$ as in Guo et al. (2018), we obtain ${\mathcal {R}}_0=0.0275<1.$ This is illustrated in Fig. 3, where we see that exposed infected hepatocytes converge toward zero after 20 days, unlike the previous one where they converge after 400 days. Also, in Fig. 3, we observe that the peaks of compartments *D* and *V* are very low compared to those observed in Fig. 2. This means that the higher the values of the parameters $\beta _1,$ $\beta _2,$ $\beta _3,$ $\tau _1,$ $\tau _2$ and $\tau _3,$ the more significant eradication of the infection within-host is expected.

Secondly, when $\beta _0=3\times 10^{-3},$ $b=0.03,$ $\tau _1=15,$ $\tau _2=2,$ $\tau _3=5,$ $a_0=0.6$ and $q=0.05,$ then we get ${\mathcal {R}}_0=2.3063>1,$ ${\mathcal {R}}_1=0.7838<1$ and ${\mathcal {R}}_2=0.1840<1.$ In this case, the initial conditions are

Initial-1: $$(H(\theta ),E(\theta ),I(\theta ),D(\theta ),V(\theta ),W(\theta ),Z(\theta ))=(5\times 10^7,0.01,0.005,0.1,0.1,0.2,0.2),$$

Initial-2: $$(H(\theta ),E(\theta ),I(\theta ),D(\theta ),V(\theta ),W(\theta ),Z(\theta ))=(5\times 10^8,0.02,0.006,0.2,0.1,2,2),$$

Initial-3: $$(H(\theta ),E(\theta ),I(\theta ),D(\theta ),V(\theta ),W(\theta ),Z(\theta ))=(5\times 10^8,0.03,0.01,0.4,0.4,5,5),$$

**Fig. 4:**
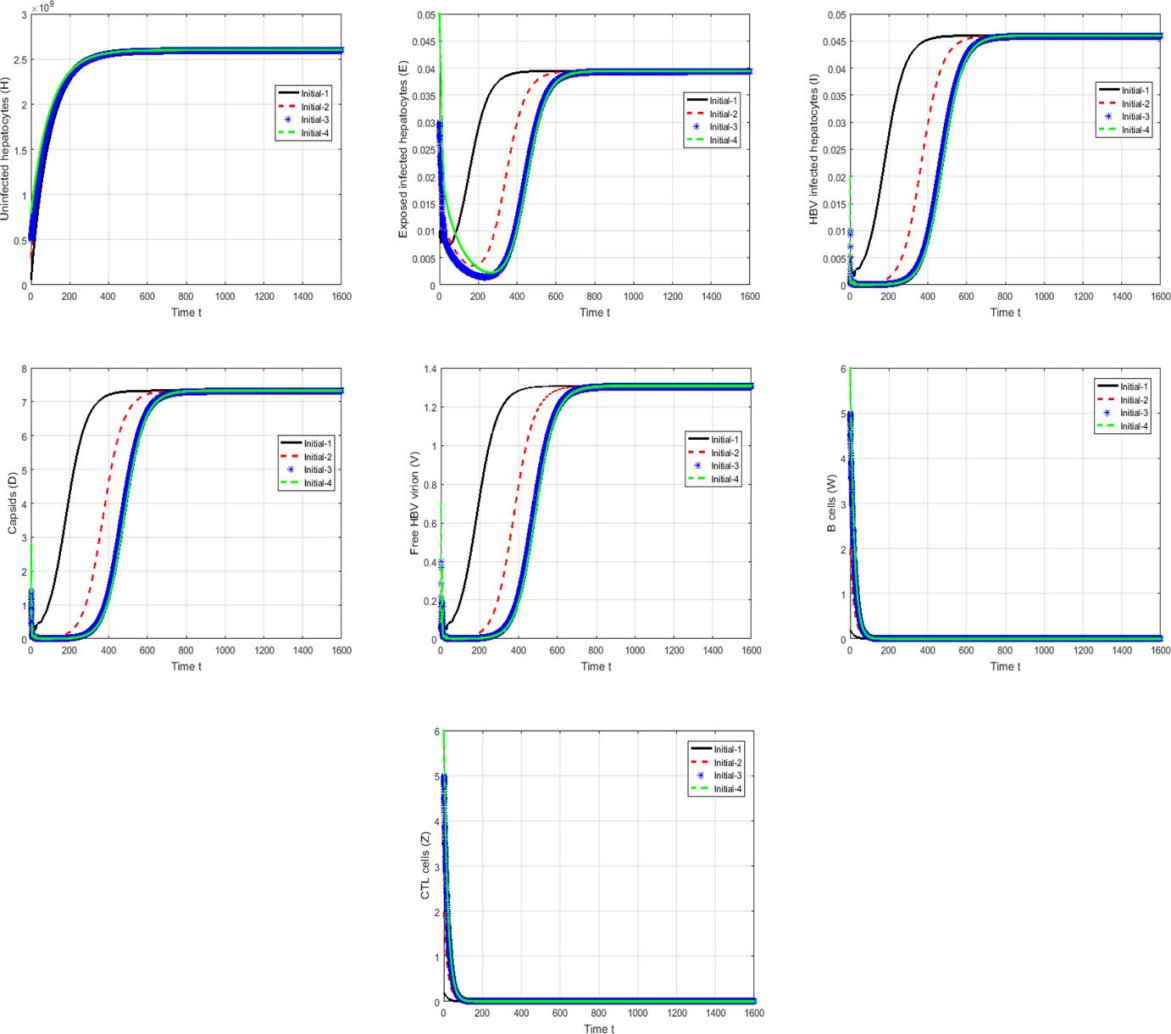
Simulations of model (4.1) using various initial conditions with $\beta _0=3\times 10^{-3},$ $b=0.03,$ $\tau _1=15,$ $\tau _2=2,$ $\tau _3=5,$ $a_0=0.6$ and $q=0.05$ so that ${\mathcal {R}}_0=2.3063>1,$ ${\mathcal {R}}_1=0.788<1$ and ${\mathcal {R}}_2=0.1840<1$

Initial-4: $$(H(\theta ),E(\theta ),I(\theta ),D(\theta ),V(\theta ),W(\theta ),Z(\theta )) =(5\times 10^8,0.05,0.02,0.7,0.7,6,6),$$

for $\theta \in [-15,0].$ This shows that the solution trajectories asymptotically tend towards the immune-free equilibrium $P_1=(2.6\times 10^9,0.0394,0.0460,7.3562,1.3063,0,0)$ as presented in Fig. 4, which support the result exhibited by Theorem 5.4. From this figure, we observe that uninfected hepatocytes increase and reach their maximum level $2.6\times 10^9,$ and that the total immune defense which is represented by the CTLs and antibodies vanish. Also, we can observe the persistence of the virus at a low level in the absence of the adaptive immunity.

**Fig. 5:**
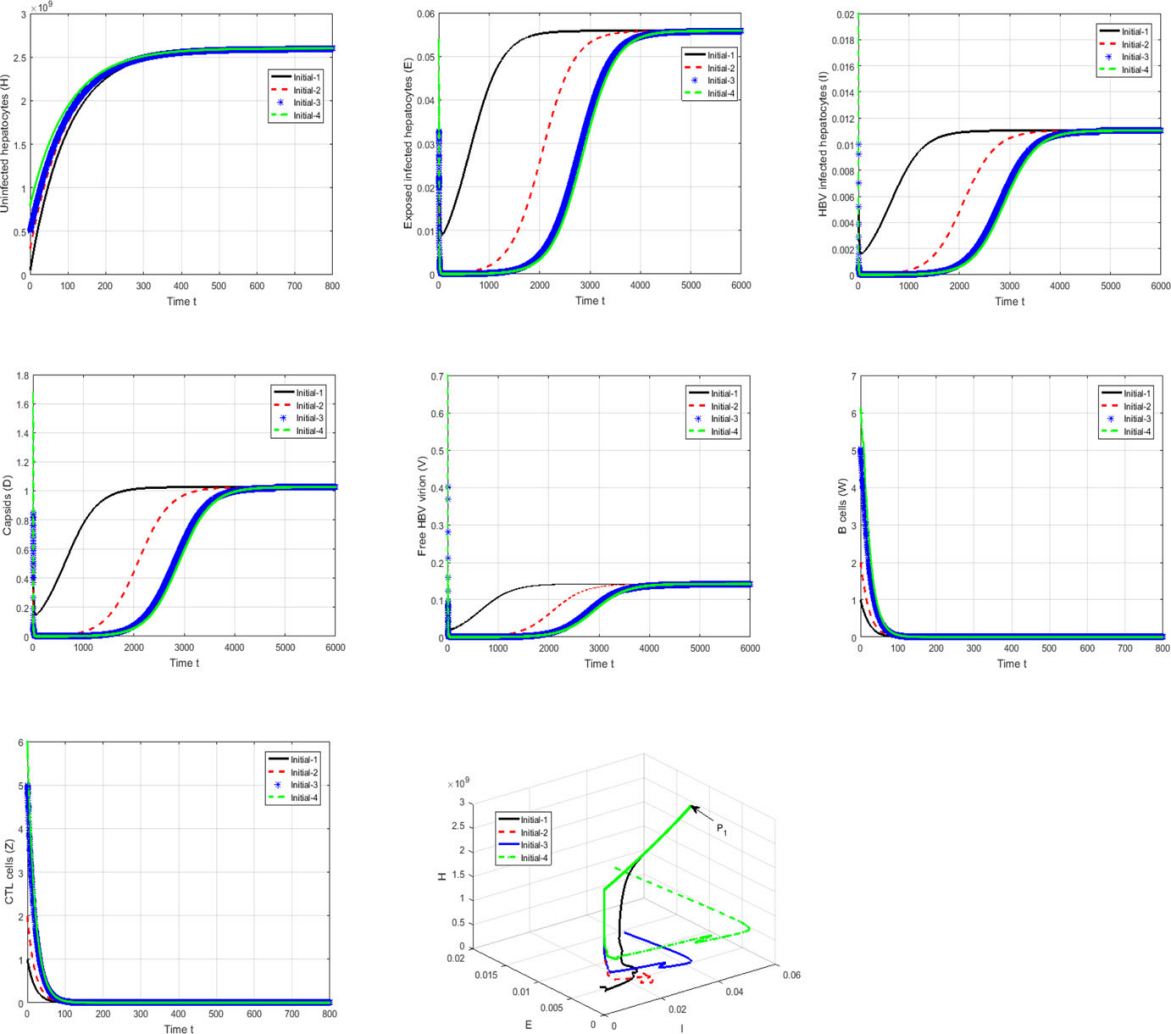
Simulations of model (4.1) using various initial conditions with $\beta _0=3\times 10^{-3},$ $\beta _1=0.2,$ $\beta _2=0.28,$ $\beta _3=0.1,$ $b=0.3,$ $b=0.03,$ $\tau _1=15,$ $\tau _2=2,$ $\tau _3=5,$ $a_0=0.6$ and $q=0.05$ so that ${\mathcal {R}}_0=1.1424>1,$ ${\mathcal {R}}_1=0.8524<1$ and ${\mathcal {R}}_2=0.0442<1$. All other parameters as in Table 1. The immune-free equilibrium $P_1=(2.6\times 10^9,0.0559,0.0110,1.0253,01424,0,0)$ is globally asymptotically stable

Here, we only consider the values of the parameters $\beta _1=0.2,$ $\beta _2=0.28,$ $\beta _3=0.1,$ and fix the other parameters as for Fig. 4. In this case we get ${\mathcal {R}}_0=1.1424>1,$ ${\mathcal {R}}_1=0.8524<1$ and ${\mathcal {R}}_2=0.0442<1.$ Fig. 5 displays the role of $\beta _1,$ $\beta _2$ and $\beta _3$ in the absence of CTL cells and B cells. It is seen from this figure that for large values of that parameters, the compartments of the infected classes remain zero up to nearly 200 days compared to those observed in Fig. 4, which are even zero for less than 250 days. This shows that, for large values of that parameters, the model studied is relevant and thus can be used to curtail the viral load within a host of an infected patient.

**Fig. 6:**
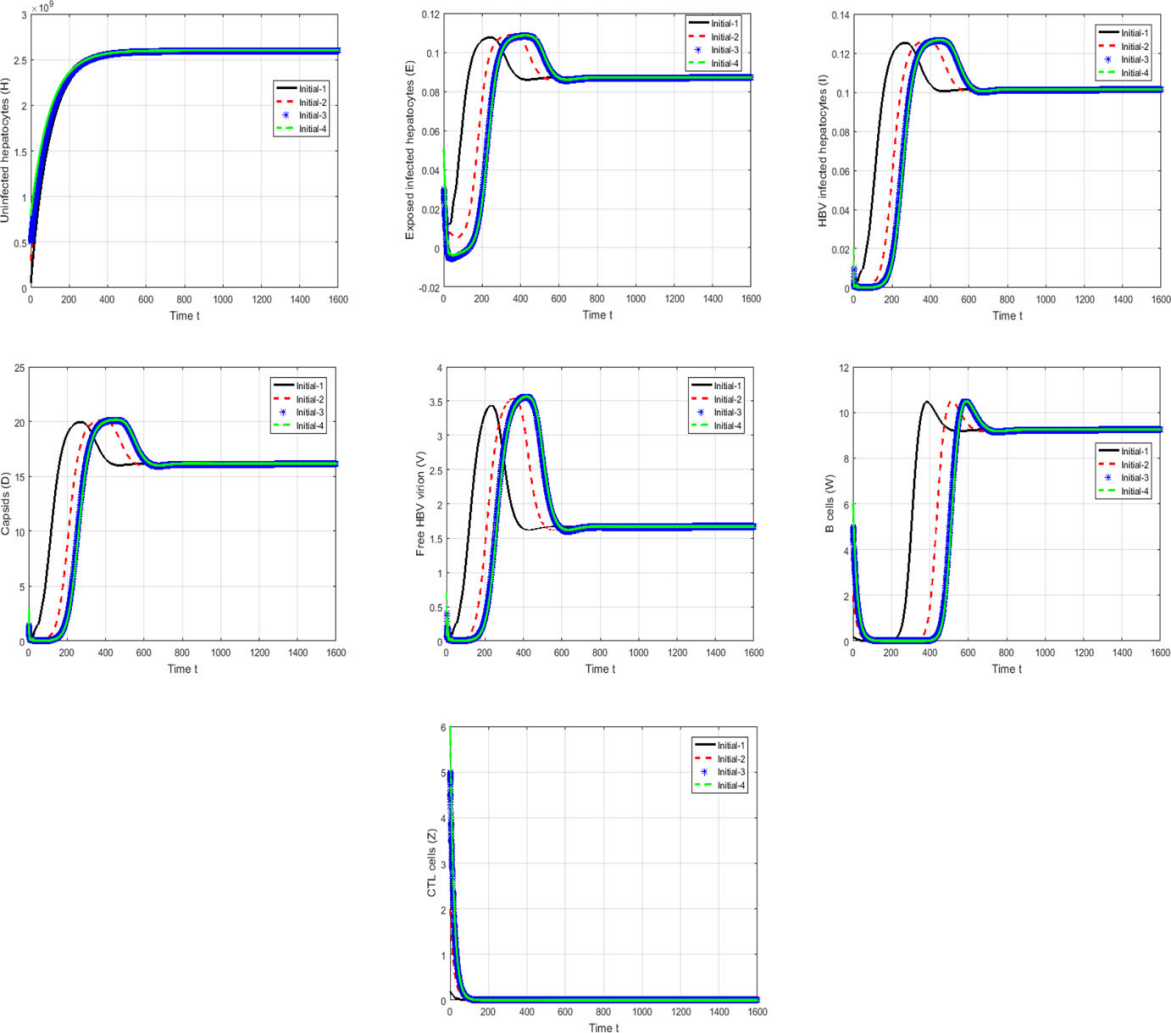
Simulations of model (4.1) using various initial conditions with $\beta _0=3\times 10^{-3},$ $b=0.03,$ $\tau _1=15,$ $\tau _2=2,$ $\tau _3=5,$ $a_0=0.3$ and $q=0.05$ so that ${\mathcal {R}}_0=4.6126>1,$ ${\mathcal {R}}_1=2.1676>1$ and ${\mathcal {R}}_3=0.4060<1$

**Fig. 7:**
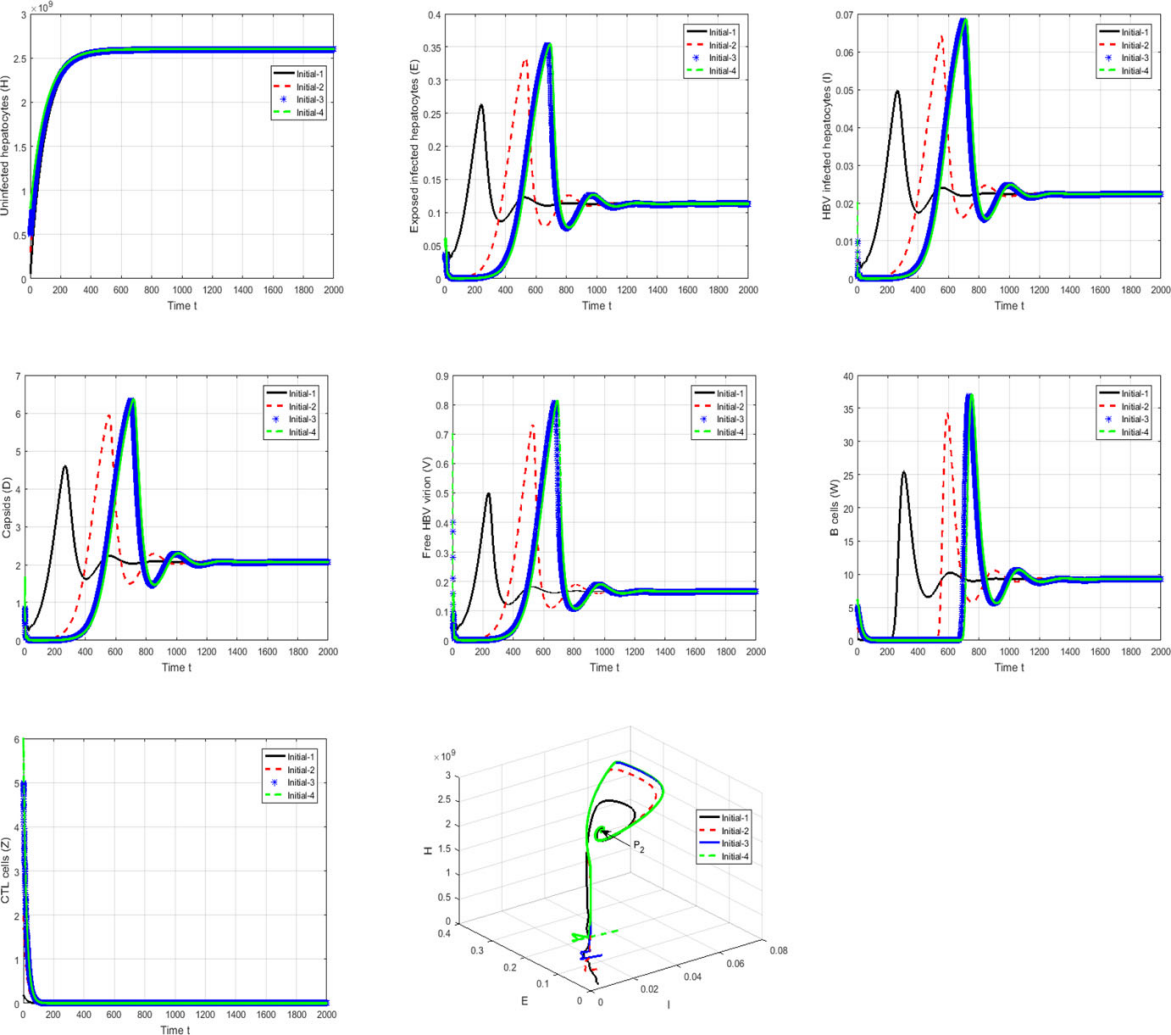
Simulations of model (4.1) using various initial conditions with $\beta _0=3\times 10^{-3},$ $\beta _1=0.2,$ $\beta _2=0.28,$ $\beta _3=0.1,$ $b=0.3,$ $\tau _1=15,$ $\tau _2=2,$ $\tau _3=5,$ $a_0=0.3$ and $q=0.05$ so that ${\mathcal {R}}_0=2.0182>1,$ ${\mathcal {R}}_1=6.1092>1$ and ${\mathcal {R}}_3=0.0895<1$. All other parameters as in Table 1. The infection equilibrium with only antibody immune defense $P_2=(2.6\times 10^9,0.1131,0.0224,2.0762,0.1667,9.2451,0)$ is globally asymptotically stable

Thirdly, when $\beta _0=3\times 10^{-3},$ $b=0.03,$ $\tau _1=15,$ $\tau _2=2,$ $\tau _3=5,$ $a_0=0.3$ and $q=0.05,$ then we find ${\mathcal {R}}_0=4.6126>1,$ ${\mathcal {R}}_1=2.1676>1$ and ${\mathcal {R}}_3=0.5088<1.$ Here, the initial conditions are as one in the second case. This set of parameters shows that the trajectories converge to the infection equilibrium with only antibody immune defense $P_2=(2.6\times 10^9,0.0871,0.1015,16.1683,1.667,9.2432,0),$ as can be seen from Fig. 6, which valid the result in Theorem 5.5. We can see from this figure that the uninfected hepatocytes always continue to reach their maximum level $2.6\times 10^9$ and we also observe that antibodies vanish during time. We remark that the free virus always persist at a low level in the absence of the B cells. Now, when $\beta _1=0.2,$ $\beta _2=0.28,$ $\beta _3=0.1,$ and the other parameters as for Fig. 6 are fixed, we see from Fig. 7 that the trajectories converge to an infection equilibrium with only antibody immune defense with a very reduced viral load. At this stage, the patient may suffer less from the symptoms.

Fourthly, when $\beta _0=3\times 10^{-3},$ $b=0.03,$ $\tau _1=4,$ $\tau _2=2,$ $\tau _3=5,$ $a_0=0.3$ and $q=0.009,$ then we have ${\mathcal {R}}_0=5.1489>1,$ ${\mathcal {R}}_2=3.2461>1$ and ${\mathcal {R}}_4=0.7669<1.$ In this case, the initial conditions are

Initial-1: $$(H(\theta ),E(\theta ),I(\theta ),D(\theta ),V(\theta ),W(\theta ),Z(\theta ))=(5\times 10^7,0.01,0.005,0.1,0.1,0.2,0.002),$$

Initial-2: $$(H(\theta ),E(\theta ),I(\theta ),D(\theta ),V(\theta ),W(\theta ),Z(\theta ))=(5\times 10^8,0.01,0.006,0.2,0.1,2,0.2),$$

Initial-3: $$(H(\theta ),E(\theta ),I(\theta ),D(\theta ),V(\theta ),W(\theta ),Z(\theta ))=(5\times 10^8,0.03,0.01,0.4,0.4,5,0.5),$$

**Fig. 8:**
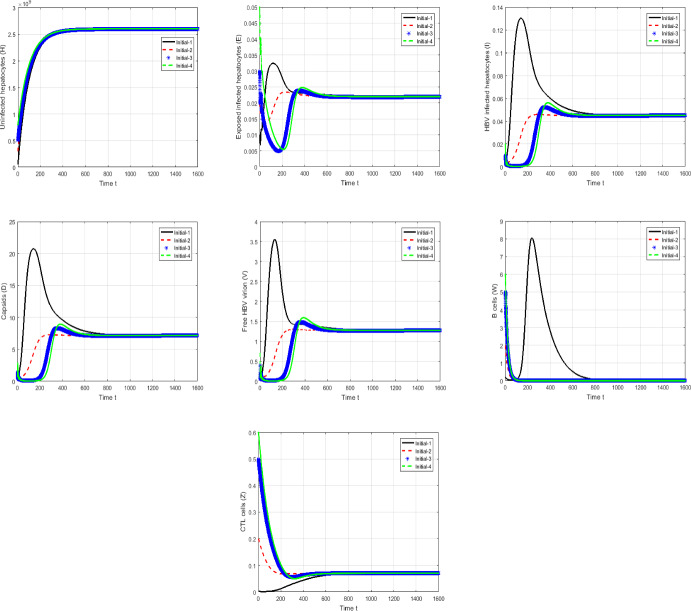
Simulations of model (4.1) using various initial conditions with $\beta _0=3\times 10^{-3},$ $b=0.03,$ $\tau _1=4,$ $\tau _2=2,$ $\tau _3=5,$ $a_0=0.3$ and $q=0.009$ so that ${\mathcal {R}}_0=5.1489>1,$ ${\mathcal {R}}_2=3.2461>1$ and ${\mathcal {R}}_4=0.7669<1$

**Fig. 9:**
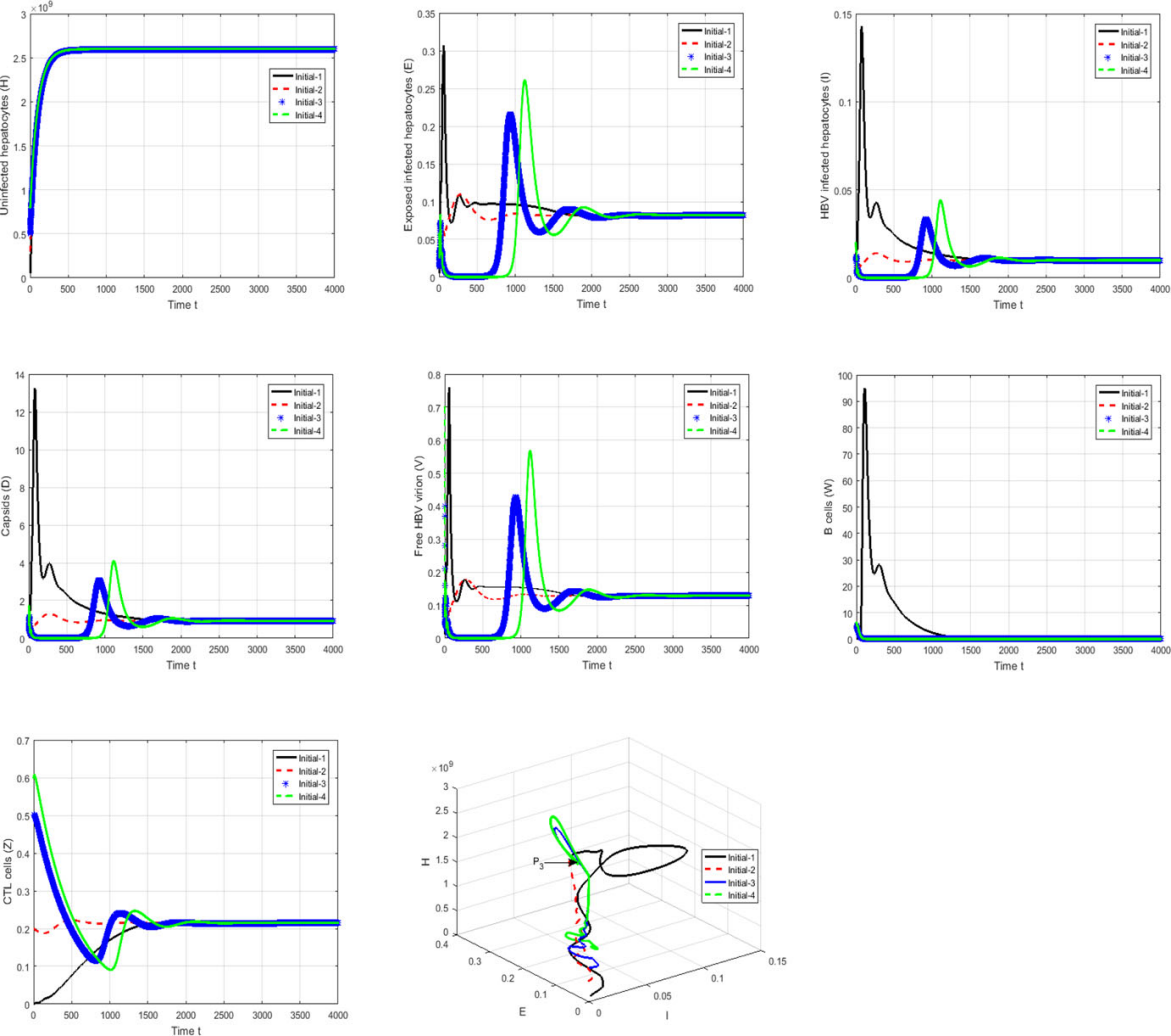
Simulations of model (4.1) using various initial conditions with $\beta _0=3\times 10^{-3},$ $\beta _1=0.2,$ $\beta _2=0.28,$ $\beta _3=0.1,$ $b=0.3,$ $\tau _1=10,$ $\tau _2=2,$ $\tau _3=5,$ $a_0=0.3$ and $q=0.002$ so that ${\mathcal {R}}_0=5.4860>1,$ ${\mathcal {R}}_2=34.8007>1$ and ${\mathcal {R}}_4=0.7734<1$. All other parameters as in Table 1. The infection equilibrium with only CTL immune response $P_3=(2.6\times 10^9,0.0823,0.0100,0.9283,0.1289,0,0.2153)$ is globally asymptotically stable

Initial-4: $$(H(\theta ),E(\theta ),I(\theta ),D(\theta ),V(\theta ),W(\theta ),Z(\theta )) =(5\times 10^8,0.05,0.02,0.7,0.7,6,0.6),$$

for $\theta \in [-5,0].$ This proves that the trajectories eventually converge to the infection equilibrium with only CTL immune response $P_3=(2.6\times 10^9,$ 0.0220,  0.0450,  7.1683,  1.2781,  0,  0.0703),  as can be observed from Fig. 8, which is actually the result exhibited by Theorem 5.6. From this figure, one can observe that the CTL immune response vanishes. This numerical result confirms that the virus persist at a low level in the absence of the cellular immunity. Again, when $\beta _1=0.2,$ $\beta _2=0.28,$ $\beta _3=0.1,$ and the other parameters as for Fig. 8 are fixed, we observe from Fig. 9 that the trajectories converge toward an infection equilibrium with only CTL immune response with a very reduced viral load. Also at this stage, the patient may suffer less from the symptoms.

Finally, when $\beta _0=3\times 10^{-3},$ $b=0.03,$ $\tau _1=15,$ $\tau _2=2,$ $\tau _3=5,$ $a_0=0.3$ and $q=0.02,$ then we get ${\mathcal {R}}_0=4.6126>1,$ ${\mathcal {R}}_1=2.1676>1,$ ${\mathcal {R}}_2=1.2719>1$ ${\mathcal {R}}_3=1.0150>1$ and ${\mathcal {R}}_4=1.7042>1.$ In this last case, the initial conditions are

Initial-1: $$(H(\theta ),E(\theta ),I(\theta ),D(\theta ),V(\theta ),W(\theta ),Z(\theta ))=(5\times 10^7,0.01,0.005,0.1,0.1,0.02,0.0002),$$

Initial-2: $$(H(\theta ),E(\theta ),I(\theta ),D(\theta ),V(\theta ),W(\theta ),Z(\theta ))=(5\times 10^8,0.02,0.006,0.2,0.1,0.02,0.0002),$$

Initial-3: $$(H(\theta ),E(\theta ),I(\theta ),D(\theta ),V(\theta ),W(\theta ),Z(\theta ))=(5\times 10^8,0.003,0.01,0.4,0.4,0.05,0.0005),$$

**Fig. 10:**
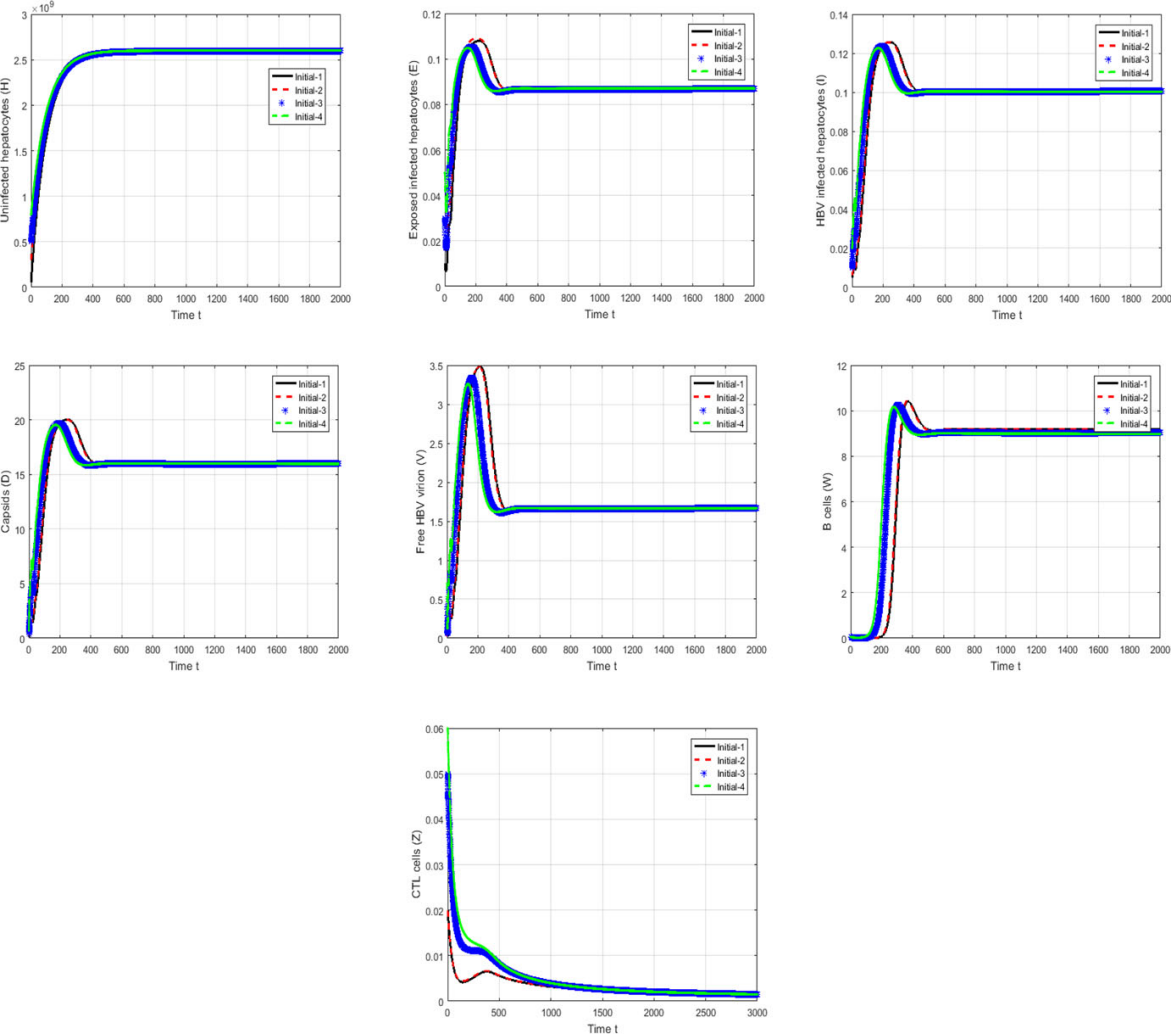
Simulations of model (4.1) using various initial conditions with $\beta _0=3\times 10^{-3},$ $\tau _1=15,$ $\tau _2=2,$ $\tau _3=5,$ $a_0=0.3$ and $q=0.02$ so that ${\mathcal {R}}_0=4.6126>1,$ ${\mathcal {R}}_1=2.1676>1,$ ${\mathcal {R}}_2=1.2719>1$ ${\mathcal {R}}_3=1.0150>1$ and ${\mathcal {R}}_4=1.7042>1$

**Fig. 11:**
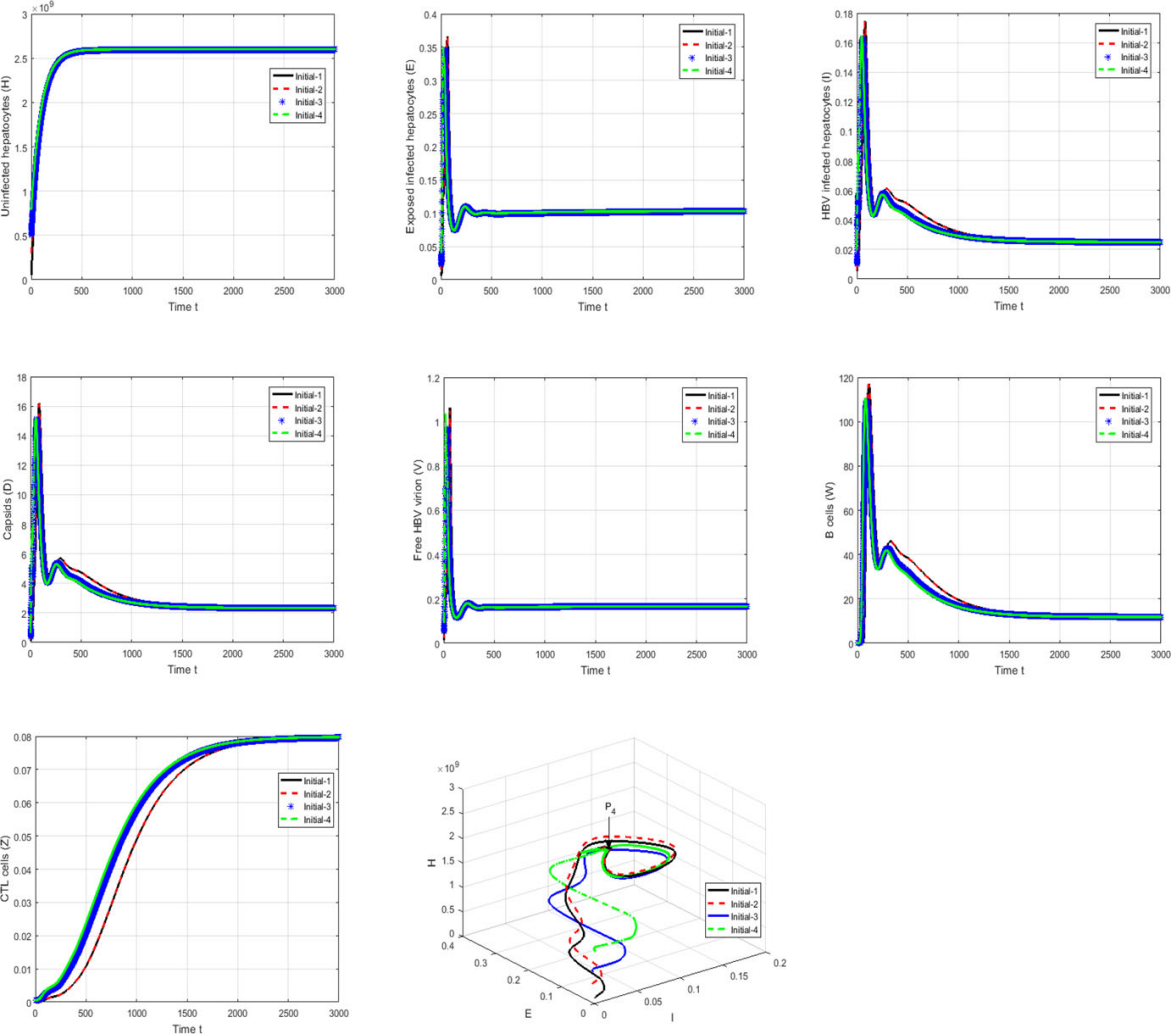
Simulations of model (4.1) using various initial conditions with $\beta _0=3\times 10^{-3},$ $\beta _1=0.2,$ $\beta _2=0.28,$ $\beta _3=0.1,$ $\tau _1=10,$ $\tau _2=2,$ $\tau _3=5,$ $a_0=0.3$ and $q=0.005$ so that ${\mathcal {R}}_0=5.4860>1,$ ${\mathcal {R}}_1=26.9161>1,$ ${\mathcal {R}}_2=13.9203>1$ ${\mathcal {R}}_3=2.4319>1$ and ${\mathcal {R}}_4=1.9336>1$. All other parameters as in Table 1. The interior infection equilibrium with both antibody and CTL immune response $P_4=(2.6\times 10^9,0.1029,0.0250,2.3207,0.1667,11.8255,0.0799)$ is globally asymptotically stable

Initial-4: $$(H(\theta ),E(\theta ),I(\theta ),D(\theta ),V(\theta ),W(\theta ),Z(\theta )) =(5\times 10^8,0.005,0.02,0.7,0.7,0.06,0.0006),$$

for $\theta \in [-15,0].$ This eventually shows that the trajectories asymptotically converge to the infection equilibrium with CTL and antibody immune defense $P_4=(2.6\times 10^9,0.0871,0.1000,15.9296,1.6667,8.9197,8.3603\times 10^{-4}),$ as can be seen from Fig. 10, which is in accord with the result exhibited by Theorem 5.7. Within the parameters of this last figure, we observe that all the infection components do not vanish as function of time. This situation corresponds to the chronic HBV infection and the adaptive immunity represented by antibodies and CTLs should play its essential role during all the period of infection, which is to decrease the HBV infection symptoms and improve the health of the patient. Moreover, we can see from Fig. 11, that when $\beta _1=0.2,$ $\beta _2=0.28,$ $\beta _3=0.1,$ this infection decreases significantly. It follows that the large values of the mortalities during the three time delays may help adaptive immune response to curtail the viral load within a host of an infected patient.

All the numerical simulations show that uninfected hepatocytes reach their maximum level $2.6\times 10^9$ and the viruses stay at an all-lime low in both the absence and the presence of the adaptive immune response which is represented by antibodies and CTLs, for all equilibrium points scenarios. Moreover, it appears that an all-lime low of exposed infected hepatocytes could lead to that of other infected compartments. Accordingly, focusing on the consideration of exposed infected hepatocytes may provide new strategies for determining new status of HBV infection, monitoring progression of hepatitis B and predicting efficiency of antiviral treatment.

**Fig. 12:**
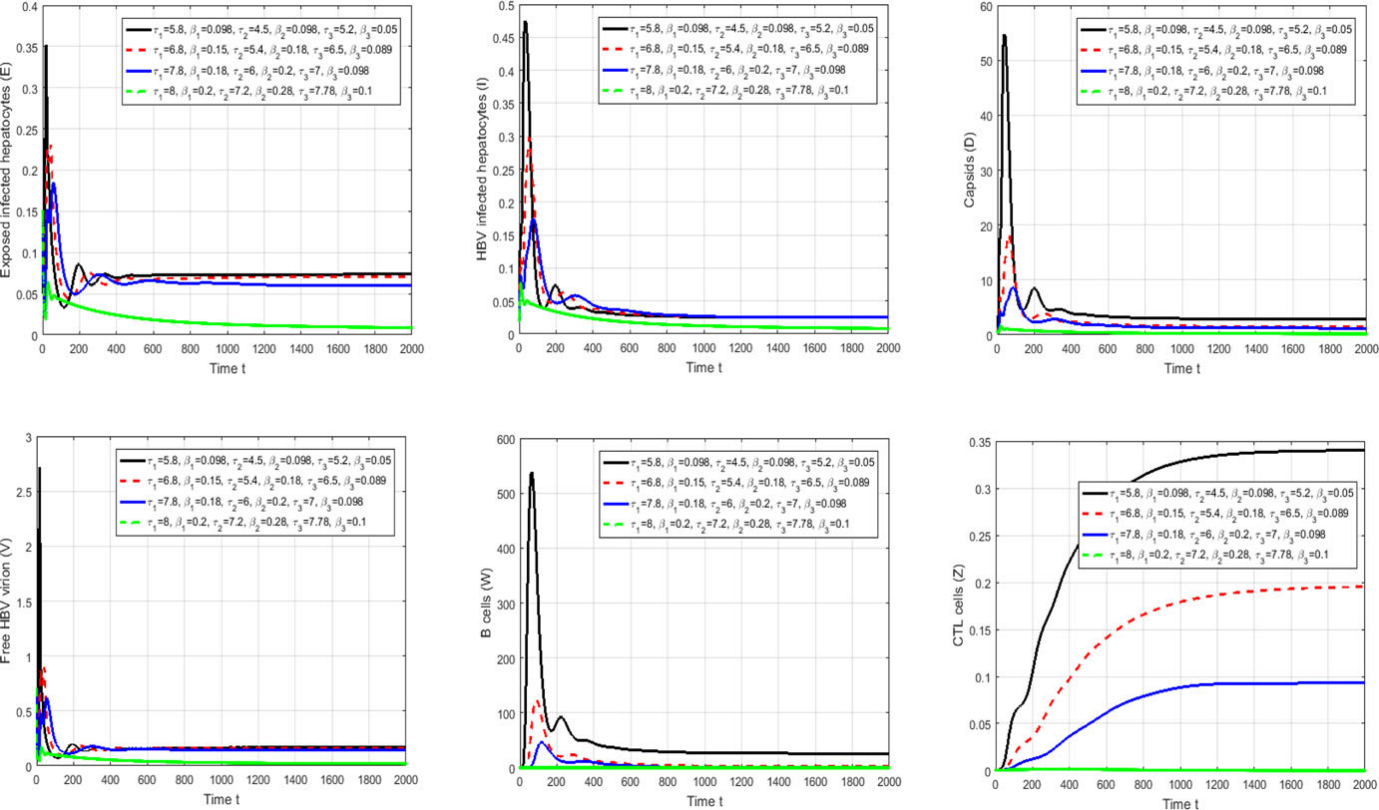
Time plots for model (4.1) with different values of $\tau _1,$ $\tau _2,$ $\tau _3,$ $\beta _1,$ $\beta _2$ and $\beta _3$

Figure 12 displays the values of $\beta _1,$ $\beta _2,$ $\beta _3,$ $\tau _1,$ $\tau _2$ and $\tau _3$ that could lead the virus to the healthy state. From this figure, we can see that by decreasing of these parameters the infection gets out of control in an infected host. Therefore, the control measures which will be established should be aimed at increasing these parameters. Moreover, it can be seen from this figure that adaptive immunity increase with the presence of productively infected hepatocytes and free virions. This means that developing a drug that take into account large values of these mortalities rates and prolongs the time delays could stimulate the immune system to respond promptly and effectively.

### Sensitivity analysis

It is vital to find out numerous aspect that contribute to the infection transmission and prevalence in order to decide the best technique for controlling or minimizing the number of affected individual. Sensitivity analysis (SA) is a method that is employed to determine the relative importance of model parameters to disease transmission and its prevalence (Chitnis et al. 2008). We carry out the analysis by computing the sensitivity indices of the basic reproduction number ${\mathcal {R}}_0$ to the parameters in the delayed model by employing local and global methods. Since there are usually errors or uncertainties in data collection and estimated values, SA is commonly employed to evaluate the model robustness to parameter values. In this section, we are interested in identifying the most influential parameters that significantly affect the basic reproduction number. These are the parameters that should be taken in to consideration when finding treatment strategies to significantly curtail the infection within a host of an infected patient.

#### Local sensitivity analysis of ${\mathcal {R}}_0$

Local sensitivity analysis deals with the sensitivity relative to change of a single parameter value. This method is based on the normalized sensitivity index of ${\mathcal {R}}_0.$ The corresponding variance in the state variable, by the variation of a parameter can be computed through the normalized forward sensitivity indices. Let $\Psi$ be denote the generic parameter of the delay system (4.1). The normalized forward sensitivity index of $\Psi$ denoted ${\mathcal {S}}_\Psi$ is the number of the corresponding normalized changes (Diekmann and Heesterbeek 2000). Since ${\mathcal {R}}_0$ is a differentiable function of the parameters, then according to the literature (Gjorgjieva et al. 2005), the normalized sensitivity index for parameter $\Psi$ can alternately be defined, in the form of partial derivatives as give below

$$\begin{aligned} {\mathcal {S}}_\Psi =\dfrac{\Psi }{{\mathcal {R}}_0}\dfrac{\partial {\mathcal {R}}_0}{\partial \Psi }, \end{aligned}$$

where $\Psi$ represents a parameter in the quantity ${\mathcal {R}}_0$ and the expression ${\mathcal {S}}_\Psi$ indicates how sensitive ${\mathcal {R}}_0$ is to a change in this parameter $\Psi .$

When determining the sensitivity index of each parameter, we use $\beta _1=0.2,$ $\beta _2=0.28,$ $\beta _3=0.1,$ $\tau _1=5.8,$ $\tau _2=6,$ and $\tau _3=4,$ from Guo et al. (2018), $a_0=0.3$ and other parameters from Table 1.

**Table 2:** Sensitivity indices for ${\mathcal {R}}_0$ with respect to parameters for the delayed model (4.1)

Parameters	Sensitivity index	Value	Parameters	Sensitivity index	Value
*k*	${\mathcal {S}}_k$	$+1$	$\delta$	${\mathcal {S}}_\delta$	$-1.0574$
$\beta _0$	${\mathcal {S}}_{\beta _0}$	$+1$	$\beta _1$	${\mathcal {S}}_{\beta _1}$	$-1.1600$
$\mu$	${\mathcal {S}}_\mu$	$-1$	$\beta _2$	${\mathcal {S}}_{\beta _2}$	$-1.6800$
$a_0$	${\mathcal {S}}_{a_0}$	$-1$	$\beta _3$	${\mathcal {S}}_{\beta _3}$	$-0.4000$
*d*	${\mathcal {S}}_d$	$-1.2821\times 10^{-9}$	$\tau _1$	${\mathcal {S}}_{\tau _1}$	$-1.1600$
$s_0$	${\mathcal {S}}_{s_0}$	$+0.0333$	$\tau _2$	${\mathcal {S}}_{\tau _2}$	$-1.6800$
$\alpha$	${\mathcal {S}}_\alpha$	$+0.0574$	$\tau _3$	${\mathcal {S}}_{\tau _3}$	$-0.4000$

**Fig. 13:**
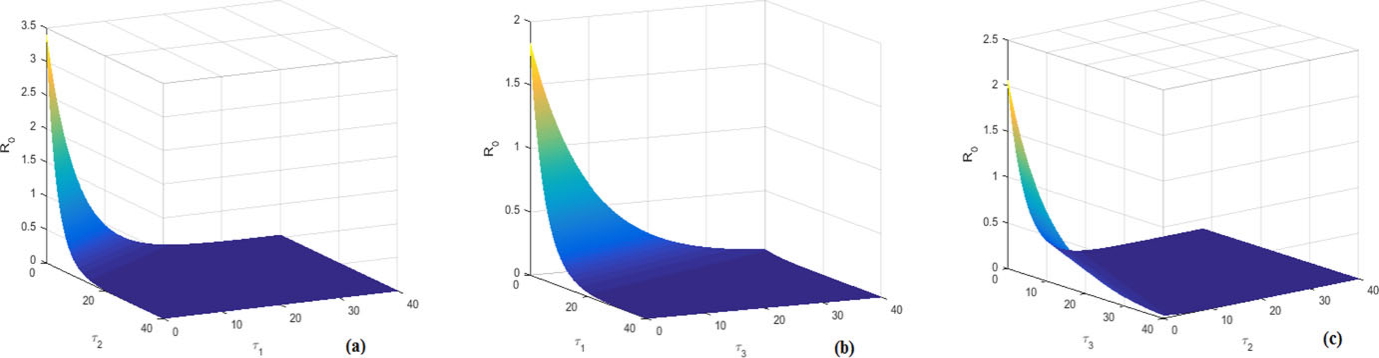
Simulations of the basic reproduction number ${\mathcal {R}}_0$ versus some parameters: (a) ${\mathcal {R}}_0$ versus $\tau _1$ and $\tau _2$, (b) ${\mathcal {R}}_0$ versus $\tau _1$ and $\tau _3$, (c) ${\mathcal {R}}_0$ versus $\tau _2$ and $\tau _3$

**Fig. 14:**
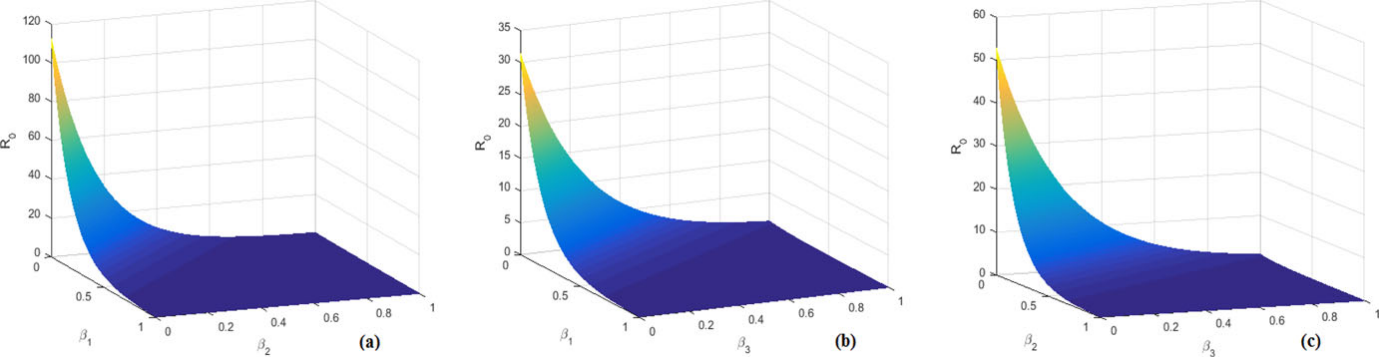
Simulations of the basic reproduction number ${\mathcal {R}}_0$ versus some parameters: (a) ${\mathcal {R}}_0$ versus $\beta _1$ and $\beta _2$, (b) ${\mathcal {R}}_0$ versus $\beta _1$ and $\beta _3$, (c) ${\mathcal {R}}_0$ versus $\beta _2$ and $\beta _3$

It is observed from Table 2 that the parameters *k*,  $\beta _0,$ $\alpha$ and $s_0$ respectively have a positive influence in the value of ${\mathcal {R}}_0.$ This indicates that the increase or the decrease of these parameters say by 10%, then ${\mathcal {R}}_0$ will increase or decrease by 10%, 10%, 0.7%, and 0.3% respectively. Note however that for the choice of the parameter values, $\alpha$ and $s_0$ have a weak positive influence in the value of ${\mathcal {R}}_0.$ The index for parameter $\delta ,$ representing the death rate of productively infected hepatocytes and Capsids, shows that increasing its value by 10% will decrease the value of ${\mathcal {R}}_0$ by 10.6%. In a similar way, the index for parameters $\tau _1,$ $\tau _2,$ $\tau _3,$ $\beta _1,$ $\beta _2$ and $\beta _3$ which denote the time necessary for exposed cells to convert to productively infected hepatocytes, the time for capsids to become mature, the time needed for newly produced HBV DNA-containing capsids to become free virions and the mortalities during the three time delays respectively, show that increasing their values by 10% will decrease ${\mathcal {R}}_0$ almost by 11.6%, 16.8%, 4%, 11.6%, 16.8% and 4% respectively. Clearly, for the choice of fixed parameter values, the percentages of the parameter $\delta ,$ $\tau _1,$ $\tau _2,$ $\beta _1,$ and $\beta _2$ remain higher than any percentage consider. It follows from this analysis that a higher death rate of productively infected hepatocytes and Capsids $\delta ,$ the delays $\tau _1,$ and $\tau _2,$ and the death rates $\beta _1,$ and $\beta _2$ decrease sufficiently ${\mathcal {R}}_0.$ Using $\beta _1=0.2,$ $\beta _2=0.28,$ $\beta _3=0.1,$ $\tau _1=5.8,$ $\tau _2=6,$ and $\tau _3=4,$ from Guo et al. (2018), $a_0=0.3$ and other parameters from Table 1, the plot results displayed in Fig 13 and 14 illustrate the role of $\tau _1,$ $\tau _2,$ $\tau _3$ $\beta _1,$ $\beta _2$ and $\beta _3$ on the basic reproduction number ${\mathcal {R}}_0,$ from which we see that ${\mathcal {R}}_0$ decreases whenever the parameters $\tau _1,$ $\tau _2,$ $\tau _3,$ $\beta _1,$ $\beta _2$ and $\beta _3$ increase. Thus it is clear as argued in Guo et al. (2018) that the neglect of the mortalities during the three time delays must result in increase in size of ${\mathcal {R}}_0.$ Also, from the plot result Fig 14, we observe that the basic reproduction number ${\mathcal {R}}_0$ become large enough whenever the death rates $\beta _1,$ $\beta _2$ and $\beta _3$ tend toward 0. From Theorem 5.3, we have that the infection-free equilibrium $P_0$ is globally asymptotically stable whenever the value of ${\mathcal {R}}_0$ is below one, which means that the viruses are cleared and the infection wipe out. Therefore, in an effort to eliminate the HBV infection, we need to reduce the value of ${\mathcal {R}}_0$ to a level lower than unity by increasing the value of $\tau _1,$ $\tau _2$ and $\tau _3.$ This suggest that, a good strategy to eradicate or to control HBV infection within a host should concentrate on any drugs that may prolong the values of these three delays.

#### Uncertainty and global sensitivity analysis of ${\mathcal {R}}_0$

Local sensitivity analysis focus more on a single input’s behavior while other parts remain the same. In other words, it evaluates the outcomes of individual parameters at particular points in parameter space without taking into account the combined variability resulting from simultaneous consideration of all input parameters. In this section, we carry out a global SA to examine the sensitivity with regard to the entire parameter distribution in a wider range of the parameter space. The baseline values of parameters are given in Table 1, except $\beta _1=0.2,$ $\beta _2=0.28,$ $\beta _3=0.1,$ $\tau _1=5.8,$ $\tau _2=6,$ $\tau _3=4,$ and $a_0=0.3,$ and the range values of these parameters in Table 3.

**Table 3:** Parameter value ranges of system (2.1) used as input for the LHS method

Parameters	Range	Parameters	Range	Parameters	Range
$s_0$	$[5.04\times 10^4,\,2.6\times 10^9]$	*a*	$[25\times 10^{-8},\,1.5]$	$\beta _0$	$[0.001,\,0.2]$
*d*	$[0.0039,\,0.1]$	*q*	$[0.002,\,0.5]$	$\beta _1$	$[0.001,\,1]$
*p*	$[0.2,\,0.98]$	$\tau _1$	$[1,\,15]$	$\beta _2$	$[0.001,\,1]$
*k*	$[50,\,280]$	$\tau _2$	$[1,\,14]$	$\beta _3$	$[0.001,\,1]$
$\alpha$	$[0.58,\,0.99]$	$\tau _3$	$[1,\,15]$	$\delta$	$[0.03,\,0.1]$
*r*	$[0.006,\,0.9]$	$a_0$	$[0.01,\,0.95]$	$\mu$	$[0.693,\,6]$
*b*	$[0.03,\,0.5]$	$b_0$	$[0.01,\,2.25]$	*c*	$[0.01,\,0.3]$

**Fig. 15:**
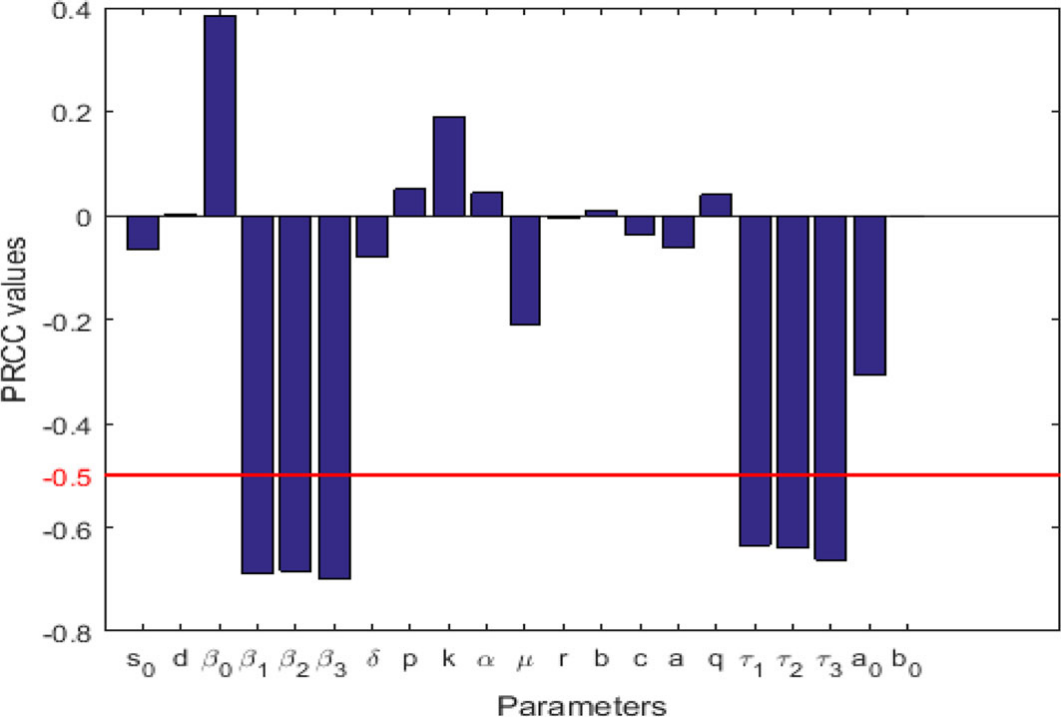
The graphs of PRCC values between ${\mathcal {R}}_0$ and each parameter for 1000 simulations

It is worth noting that, variations of these parameters in our compartmental delay model lead to uncertainty to model predictions since ${\mathcal {R}}_0$ varies with parameters. Following the approach of Marino et al. (2008), we use partial rank correlation coefficients (PRCC) and Latin Hypercube Sampling (LHS) to explore each parameter in the basic reproduction number ${\mathcal {R}}_0$ of model (4.1). To implement the LHS scheme, a uniform distribution is chosen for all parameters. The sets of input parameter values sampled using the LHS method were used to run 1000 simulations. The results of the PRCC computation between ${\mathcal {R}}_0$ and each parameter of model (4.1) are displayed in Table 4. Figure 15 represents the SA plot of ${\mathcal {R}}_0.$

**Table 4:** PRCC between ${\mathcal {R}}_0$ and each parameter

Parameters	PRCCs	*P* values	Parameters	PRCCs	*P* values
$s_0$	$-0.0646$	0.0431	$\beta _0$	0.3844	$7.3533E{-}36$
*d*	0.0011	0.9719	$$\beta _1$$	$$-0.6882^{**}$$	$$1.9814E{-}138$$
*p*	0.0504	0.1148	$$\beta _2$$	$$-0.6832^{**}$$	$$1.0282E{-}135$$
*k*	0.1892	$$2.3710E{-}09$$	$$\beta _3$$	$$-0.7002^{***}$$	$$2.8325E{-}145$$
$\alpha$	0.0427	0.1813	$\delta$	$-0.0792$	0.0132
*r*	$-0.0064$	0.8414	$\mu$	$-0.2094$	$3.5962E{-}11$
*b*	0.0101	0.7533	*c*	$-0.0384$	0.2300
*a*	$-0.0634$	0.0471	*q*	0.0397	0.2145
$$\tau _1$$	$$-0.6347^{**}$$	$$1.3373E{-}111$$	$$\tau _2$$	$$-0.6393^{**}$$	$$1.0397E{-}113$$
$$\tau _3$$	$$-0.6627^{**}$$	$$5.7146E{-}125$$	$$a_0$$	$$-0.3072$$	$$7.2366E{-}23$$
$b_0$	$-0.0007$	0.9828			

The most influential parameters in model (4.1) have a PRCC value greater than 0.5 or less than $-0.5$ and a *P* value less than 0.05 (Gomero 2012). Table 4.3 and Fig. 15 show that the parameter $\beta _0$ have the highest influence on the reproduction number ${\mathcal {R}}_0,$ followed in decreasing order by the parameters $\beta _1,$ $\beta _2,$ $\beta _3$ $\tau _1,$ $\tau _2,$ and $\tau _3.$ The parameters which do not have almost any effect on ${\mathcal {R}}_0$ are $s_0,$ *d*,  *p*,  $\alpha ,$ *r* *b*,  *a*,  $b_0,$ *c*,  and *q*,  where the saturation factor measuring the inhibitory or physiological effect $b_0,$ is the least sensitive of the parameters. It can be seen that parameters $\tau _1,$ $\tau _3,$ and $\tau _3,$ permit us to considerably reduce the reproduction number. Thus, the global SA consistently reinforces our suggestion that the most effective way to reduce the infection within a host is to increase the three time delays. It follows that, the strategies and action presented on these three parameters will be useful in order that the spread of infection enters a downward course.

#### Sensitivity analysis of Infected states of model (4.1)

In this subsection, with 1000 runs of LHS, we calculate the PRCC between infected compartments *E*(*t*),  *I*(*t*),  *D*(*t*) and *V*(*t*) and each parameters of model (4.1). The results is represented in Tables 5, t002, t003 and 8. Here too, the most influential parameters in model (4.1) have a PRCC value greater than 0.5 or less than $-0.5$ and a *P* value less than 0.05.

From Tables 5, t002, t003 and 8, the following facts can be observed.

1) For the value of *E*,  the most important parameters are $\beta _0$ and *k*. The parameters which do not have almost any effects on the variation of *E* are $s_0,$ *d*,  *p*,  $\alpha ,$ *r*,  *a*,  $\tau _1,$ $\tau _2,$ $\tau _3,$ $b_0$ and *q*. The least sensitive parameter is *a*,  the CTL activation rate.

2) For the value of *I*,  the most important parameters are $\beta _0,$ *k* and $\beta _1.$ The least sensitive parameters are *d* and $b_0,$ the natural death rate of healthy hepatocytes and the Crowley-Martin coefficient respectively.

3) For the value of *D*,  the most important parameters are *k* and $\beta _0.$ The least sensitive parameter is *p*,  the rate at which productively infected hepatocytes are removed by CTL cells.

4) For the value of *V*,  the most important parameters are *k* and $\beta _0.$ The least sensitive parameter is *p*,  the CTL effectiveness.

Apart from the time delays parameters whose model is sensitive to their variations, other parameters, such as *k*,  $\beta _0$ and $\beta _1,$ have a considerable impact on the value of the basic reproduction number ${\mathcal {R}}_0$ and the number of infected compartments. Consequently, it is crucial to take into account other favorable and adequate strategies in the elimination of HBV infection.

**Table 5:** PRCC between exposed infected hepatocytes E and each parameter

Parameters	PRCCs	*P* values	Parameters	PRCCs	*P* values
$$s_0$$	0.0508	0.6460	$$\beta _0$$	$$0.6688^{**}$$	$$4.4041E{-}128$$
*d*	0.0053	0.2597	$\beta _1$	$-0.4603$	$1.5276E{-}52$
*p*	0.0066	0.8372	$\beta _2$	$-0.1260$	$7.6946E{-}05$
*k*	$0.5089^{*}$	$1.1211E{-}65$	$\beta _3$	$-0.1270$	$6.6923E{-}05$
$\alpha$	$-0.0403$	0.2077	$\delta$	$-0.1333$	$2.8383E{-}05$
*r*	$-0.0123$	0.7009	$\mu$	$-0.2130$	$1.6293E{-}11$
*b*	$-0.2588$	$1.8235E{-}16$	*c*	0.2340	$1.1733E{-}13$
*a*	0.0030	0.9242	*q*	0.0432	0.1767
$\tau _1$	$-0.0947$	0.0030	$\tau _2$	$-0.0755$	0.0181
$\tau _3$	$-0.0748$	0.0191	$a_0$	$-0.3866$	$2.7971E{-}36$
$b_0$	0.0116	0.7158

**Table 6:** PRCC between productively infected hepatocytes I and each parameter

Parameters	PRCCs	*P* values	Parameters	PRCCs	*P* values
$s_0$	$-0.0580$	0.0693	$\beta _0$	$0.5808^{*}$	$1.8429E{-}89$
*d*	0.0017	0.9588	$\beta _1$	$-0.5076^*$	$2.6384E{-}65$
*p*	$-0.0641$	0.0447	$\beta _2$	$-0.1200$	$1.6330E{-}04$
*k*	$0.5190^*$	$1.1258E{-}68$	$\beta _3$	$-0.1050$	$9.9187E{-}04$
$\alpha$	0.0094	0.7685	$\delta$	$-0.1251$	$8.5621E{-}05$
*r*	$-0.0122$	0.7021	$\mu$	$-0.1739$	$4.3203E{-}08$
*b*	$-0.1825$	$8.6958E{-}09$	*c*	0.1148	$3.1568E{-}04$
*a*	$-0.0655$	0.0404	*q*	0.1740	$4.1890E{-}08$
$\tau _1$	$-0.3148$	$5.4938E{-}24$	$\tau _2$	$-0.1160$	$2.7266E{-}04$
$\tau _3$	$-0.1484$	$3.0965E{-}06$	$a_0$	$-0.3030$	$2.9267E{-}22$
$b_0$	0.0017	0.9580

**Table 7:** PRCC between HBV DNA-containing capsids D and each parameter

Parameters	PRCCs	*P* values	Parameters	PRCCs	*P* values
$$s_0$$	0.0312	0.3291	$$\beta _0$$	$$0.6161^{**}$$	$$1.6853E{-}103$$
*d*	0.0455	0.1549	$\beta _1$	$-0.4727$	$1.0647E{-}55$
*p*	$-0.0099$	0.7559	$\beta _2$	$-0.3075$	$6.6309E{-}23$
*k*	$$0.6236^{**}$$	$$1.0529E{-}106$$	$$\beta _3$$	$$-0.1567$$	$$8.2828E{-}07$$
$\alpha$	$-0.0412$	0.1977	$\delta$	$-0.1948$	$7.7552E{-}10$
*r*	0.0178	0.5770	$\mu$	$-0.1811$	$1.1335E{-}08$
*b*	$-0.2390$	$3.3903E{-}14$	*c*	0.2139	$1.3299E{-}11$
*a*	$-0.0971$	0.0023	*q*	0.1499	$2.4371E{-}06$
$\tau _1$	$-0.2927$	$8.2531E{-}21$	$\tau _2$	$-0.1043$	0.0011
$\tau _3$	$-0.1053$	$9.6041E{-}04$	$a_0$	$-0.3426$	$2.2447E{-}28$
$b_0$	0.0399	0.2117

**Table 8:** PRCC between free viruses V and each parameter

Parameters	PRCCs	*P* values	Parameters	PRCCs	*P* values
$s_0$	0.0108	0.7350	$\beta _0$	0.5271	$3.7123E{-}71$
*d*	$-0.0099$	0.7569	$\beta _1$	$-0.3713$	$2.1433E{-}33$
*p*	$-0.0061$	0.8491	$\beta _2$	$-0.2151$	$1.0197E{-}11$
*k*	0.5221	$1.2645E{-}69$	$\beta _3$	$-0.2097$	$3.3483E{-}11$
$\alpha$	$-0476$	0.1368	$\delta$	$-0.1541$	$1.2526E{-}06$
*r*	0.0975	0.0023	$\mu$	$-0.2293$	$3.7120E{-}13$
*b*	$-0.3328$	$9.0226E{-}27$	*c*	0.2510	$1.5044E{-}15$
*a*	0.0241	0.4516	*q*	0.0436	0.1723
$\tau _1$	$-0.1809$	$1.1675E{-}08$	$\tau _2$	$-0.0869$	0.0065
$\tau _3$	$-0.1086$	$6.5700E{-}04$	$a_0$	$-0.1570$	$7.7872E{-}07$
$b_0$	0.0131	0.6819			

## Conclusion and discussion

In this paper we have formulated and analyzed a new model for HBV infection process in vivo. The obtained model includes intracellular HBV DNA-containing capsids, adaptive immunity, exposed infected hepatocytes, three-time delays and general incidence functional. For the proposed model, we have established five threshold parameters, namely, the basic reproduction number ${\mathcal {R}}_0,$ the antibody immune response activation reproduction number ${\mathcal {R}}_1,$ the CTL immune defense activation reproduction number ${\mathcal {R}}_2,$ the competitive CTL immune response reproduction number ${\mathcal {R}}_3,$ and the competitive antibody immune response reproduction number ${\mathcal {R}}_4,$ and proved the existence of five equilibrium points, namely, the infection-free equilibrium $P_0,$ the immune-free equilibrium $P_1,$ the infection equilibrium with only antibody immune defense $P_2,$ the infection equilibrium with only CTL immune response $P_3$ and the interior infection equilibrium with both antibody and CTL immune response $P_4.$ Under assumptions $(B_1)$-$(B_3)$ and $(B_4),$ the stability properties of the five equilibria were investigated by constructing five suitable Lyapunov functionals and using LaSalle’s invariance principle, as well as the linearization method. More precisely, we have proved that $P_0$ is globally asymptotically stable whenever ${\mathcal {R}}_0\le 1.$ This implies that all solutions trajectories converge towards $P_0$ and the disease ultimately dies out. When ${\mathcal {R}}_0>1,$ $P_0$ becomes unstable and the four other aforementioned equilibrium points appear. Concretely, we have proved that $P_1$ is globally asymptotically stable whenever ${\mathcal {R}}_1\le 1$ and ${\mathcal {R}}_2\le 1$ and becomes unstable when ${\mathcal {R}}_1>1$ or ${\mathcal {R}}_2>1.$ Next, we have shown that when ${\mathcal {R}}_1>1,$ $P_2$ exists, and it is globally asymptotically stable if ${\mathcal {R}}_3\le 1$ and becomes unstable when ${\mathcal {R}}_3>1.$ The existence of $P_3$ is obtained when ${\mathcal {R}}_2>1,$ and it is globally asymptotically stable if ${\mathcal {R}}_4\le 1$ and becomes unstable if ${\mathcal {R}}_4>1.$ Finally, we have shown that the last equilibrium point $P_4$ is globally asymptotically stable if it exists and the five threshold indices are strictly greater than unity. This indicates that both CTL and antibody immune defense will be activated only when ${\mathcal {R}}_1>1,$ ${\mathcal {R}}_2>1,$ ${\mathcal {R}}_3>1$ and ${\mathcal {R}}_4>1.$ In this case, all solutions trajectories tend to $P_4$ and the disease will be persistent in the host. This argument and some previous one indicate that the activation of one or both CTL and antibody immune response is either able to diminish the viral load by blocking the HBV infection process or unable to eradicate the free viruses from the infected human liver. The numerical simulations implemented in Sect. 6 exhibit the behavior of solutions in time. Furthermore, all the seven state variables influenced by multi-time delays can better impact the viral infection progression. Therefore, the investigations in this paper can be seen as an improvement for a better understanding of HBV infection. On the other hand, the condition given in Theorem 5.3 which indicates that the infection-free equilibrium $P_0$ is globally asymptotically stable whenever ${\mathcal {R}}_0=\frac{k\alpha s_0\beta _0}{\delta \mu (d+a_0s_0)(\alpha +\delta )} e^{-\beta _1\tau _1-\beta _2\tau _2 -\beta _3\tau _3}<1,$ gives the analytical conditions under which the solution trajectories asymptotically approach towards the infection-free steady state. Thus, this can help to find treatment strategies to significantly curtail the infection within a host of an infected patient. Moreover, from the sensitivity analysis, it follows that the expression of ${\mathcal {R}}_0$ is relies on six key parameters, namely $\beta _0,$ $\beta _1,$ *k*,  $\tau _1,$ $\tau _2,$ and $\tau _3.$ This to mean that the variation of these six parameters can play a crucial role in hepatitis B treatment. In this case, the medical staff have to adapt these parameters thanks to the above expression of ${\mathcal {R}}_0$ in order to eradicate HBV infection within a host. It follows that the parameters $\beta _0,$ $\beta _1,$ *k*,  $\tau _1,$ $\tau _2,$ and $\tau _3$ might be varied to adjust hepatitis B treatment. Note that the analysis shows that ${\mathcal {R}}_0$ is a proportionally increasing function of parameter $\beta _0$ and a proportionally decreasing function of parameters $\tau _1,$ $\tau _2,$ and $\tau _3.$ Therefore, in an effort to eliminate the HBV infection, we need to reduce the value of ${\mathcal {R}}_0$ to a level lower than unity by increasing the value of $\beta _1,$ $\tau _1,$ $\tau _2,$ or $\tau _3.$ Decreased infection rate $\beta _0$ leads to disappearance of immune-free equilibrium, infection equilibrium with only antibody immune defense, infection equilibrium with only CTL immune response and interior infection equilibrium with both antibody and CTL immune response and infection-free equilibrium will become stable. This indicates the HBV eradication and that the patient is healed. Note that the discussion around these parameters shows that they play an important role insofar as if $\beta _0$ is very small, the swiftness of virus infection progression could be slowed enough. Also, if the multi-time delays and the mortalities during these time delays are very large, the density of each compartment involved (i.e., *E*,  *I*,  *D*,  *V*) could be significantly reduced over time.

Another important challenge from a therapeutic point of view is to study the factors which cause growth of free virions. From Fig. 12, we saw that by decreasing the value of the parameters $\beta _1,$ $\beta _2,$ $\beta _3,$ $\tau _1,$ $\tau _2$ and $\tau _3,$ the infection gets out of control in an infected host. Therefore, the control measures which will be established should be aimed at increasing these parameters. Moreover, it appears in all numerical simulations that an all-lime low of exposed infected hepatocytes could lead to that of other infected compartments compared to models in the literature that do not include the compartment of exposed infected hepatocytes. Thus, focusing on the consideration of a delayed model of viral infection taking in to account the exposed cells may provide new strategies for developing new antiviral drugs and design optimal combination of therapies for patients.

However, as argued in Xie et al. (2016) that time delays cannot be ignored in models for adaptive immune response, in a forthcoming work, we shall study a model (2.3) with intracellular delay, intracellular HBV DNA-containing capsids delay, virus replication delay and two adaptive immune response delays to explore five delays on how they impact the dynamical behavior of viral infection model.

## Data Availability

The codes written to run most of the simulations presented in this work can be available upon simple request to the author.
